# Degenerative xanthate transfer to olefins under visible-light photocatalysis

**DOI:** 10.3762/bjoc.14.283

**Published:** 2018-12-13

**Authors:** Atsushi Kaga, Xiangyang Wu, Joel Yi Jie Lim, Hirohito Hayashi, Yunpeng Lu, Edwin K L Yeow, Shunsuke Chiba

**Affiliations:** 1 Division of Chemistry and Biological Chemistry, School of Physical and Mathematical Sciences, Nanyang Technological University, Singapore 637371, Singapore

**Keywords:** energy transfer, olefin, photocatalysis, radical, xanthate

## Abstract

The degenerative transfer of xanthates to olefins is enabled by the iridium-based photocatalyst [Ir{dF(CF_3_)ppy}_2_(dtbbpy)](PF_6_) under blue LED light irradiation. Detailed mechanistic investigations through kinetics and photophysical studies revealed that the process operates under a radical chain mechanism, which is initiated through triplet-sensitization of xanthates by the long-lived triplet state of the iridium-based photocatalyst.

## Introduction

**Scheme 1:**
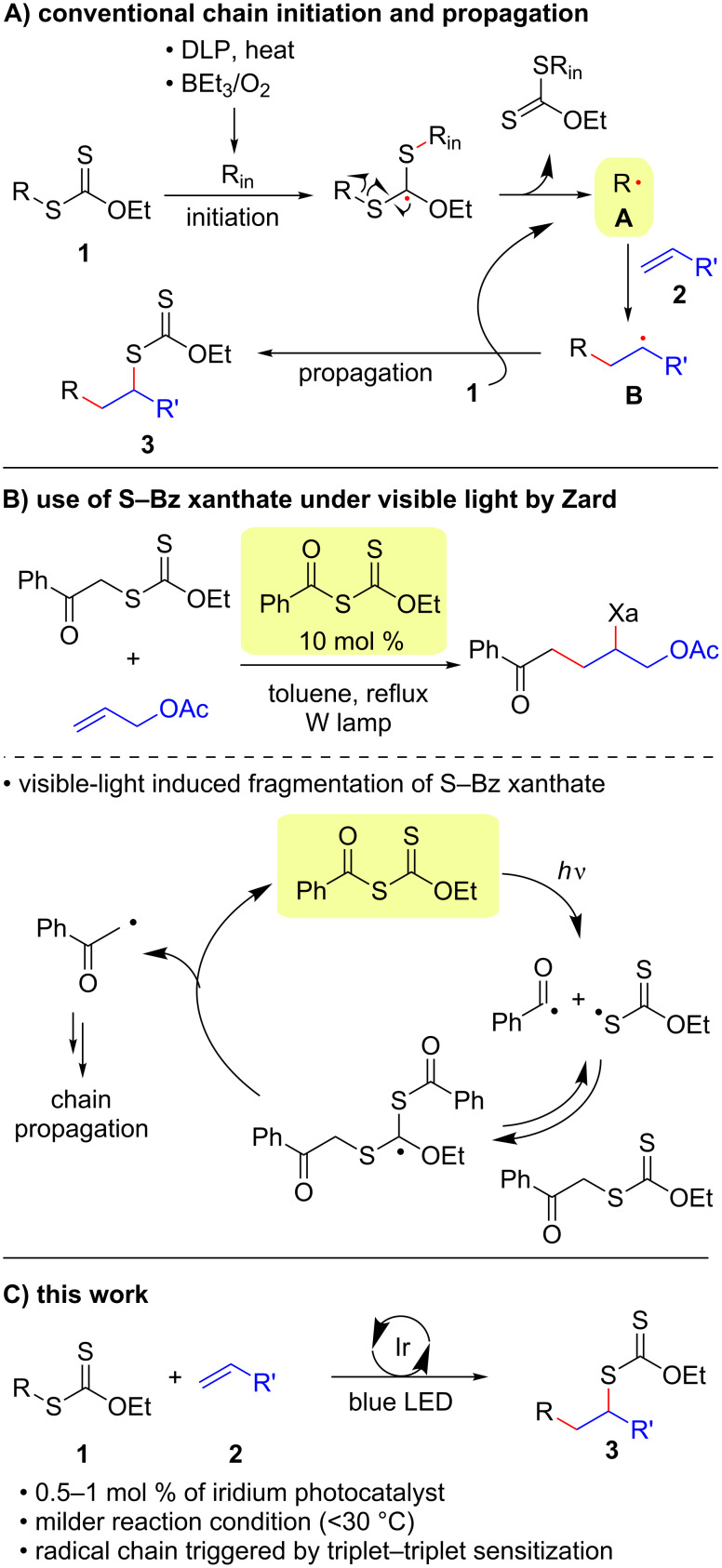
Degenerative radical transfer of xanthates to olefins.

A degenerative radical transfer of xanthates to olefins has been developed as a robust synthetic tool to create new C–C and C–S bonds in a single operation [1–13]. The method is featured by not only its capability of introducing a wide range of carbon substituents but also the ability of the installed xanthyl group in being transformed into a variety of functionalities [1–14]. This concept has also been of particular importance in the field of polymer science, known as reversible addition–fragmentation chain transfer (RAFT) polymerization [15–16]. Mechanistically, the degenerative transfer of xanthates **1** to olefins **2** proceeds through a radical chain mechanism, and thus requires an initial formation of carbon radicals **A** that add onto olefins **2**. The subsequent reaction of the resulting alkyl radicals **B** with xanthates **1** provides xanthate adducts **3** with generation of carbon radicals **A** that maintain the radical chain (Scheme 1A). Peroxide initiators such as dilauroyl peroxide (DLP) are commonly utilized [1–14], while decomposition of DLP needs a high reaction temperature and inevitably generates considerable amounts of byproducts derived from DLP that sometimes require tedious purification of the desired products. A combination of triethylborane (Et_3_B) and molecular oxygen can also initiate the reaction at lower temperature (e.g., room temperature), while the employment of Et_3_B is hampered due to its pyrophoric nature under aerobic conditions as well as undesired Et_3_B-mediated dexanthylation of α-xanthyl ketones [17–21]. As an alternative strategy, a light-driven approach has been developed [22–26], since the first degenerative transfer of xanthates using *S*-benzoyl *O*-ethyl xanthate as a photo-cleavable initiator under tungsten lamp irradiation was reported by Zard [25–26] (Scheme 1B). However, these protocols have thus far adopted energy intensive light sources. Therefore, there is still ample room for establishing new protocols to realize the degenerative transfer of xanthates onto olefins under user-friendly and milder reaction conditions. Herein, we report a photocatalytic degenerative radical transfer of xanthates to olefins using an iridium-based photocatalyst under blue LED irradiation (Scheme 1C). A series of mechanistic investigations identified that the process involves a triplet-sensitization of the xanthates by the long-lived triplet state of the iridium-based photocatalyst that triggers the radical chain process [27].

## Results and Discussion

**Scheme 2:**
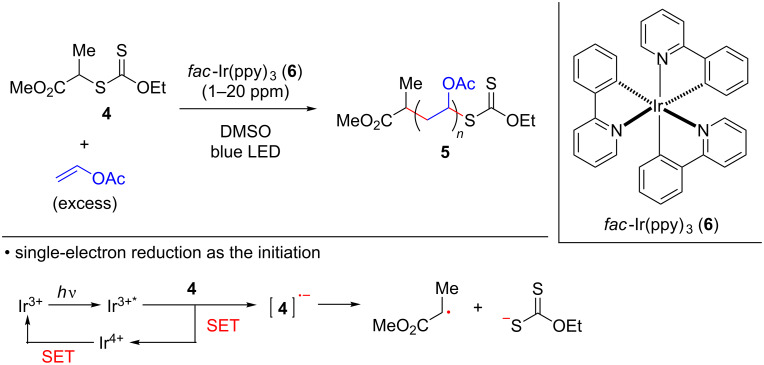
Photocatalytic RAFT polymerization of xanthate **4**.

Over the last decade, there has been a remarkable advance in synthetic chemistry that takes advantage of various chromophores (either metallic or organic) having visible-light charge transfer absorption [28–37]. In the area of polymer synthesis, visible-light-induced RAFT polymerization of xanthates with vinyl monomers under blue LED (light-emitting diode) irradiation has been reported [38–41]. Visible-light-induced single unit monomer insertion of the thiocarbonylthio compounds has also been developed for the synthesis of the sequence-controlled oligomers [41–45]. For example, the group of Boyer and Xu developed *fac*-Ir(ppy)_3_ (**6**)-catalyzed polymerization of xanthate **4** with various vinyl monomers such as vinyl acetate, providing polymers of type **5** having a high molecular weight with a narrow molecular weight distribution. It was proposed that the polymerization is initiated by single-electron reduction of xanthate **4** by the highly reducing photo-excited state of *fac*-Ir(ppy)_3_ (**6**) [46], although the details were not elucidated (Scheme 2) [39–40].

Based on these backgrounds, we wondered if the degenerative transfer of xanthates onto olefins could be facilitated by visible-light photocatalysis under milder reaction conditions. We therefore commenced our investigation with the reaction of ethyl ethoxycarbonylmethyl xanthate (**1a**) and 1-octene (**2a**) using *fac*-Ir(ppy)_3_ (**6**) in DMSO under blue LED irradiation (λ_max_ = 469 nm, Table 1, entry 1). As expected, the desired xanthate transfer was observed, while the process efficiency was not very high, forming **3aa** in only 58% yield with incomplete conversion even after stirring for 20 h. Interestingly, we found that the employment of the less reducing Ir catalysts **7** [46] and **8** [47] also worked for the process (Table 1, entries 2 and 3). Especially, the rather oxidizing [Ir{dF(CF_3_)ppy}_2_(dtbpy)](PF_6_) (**8**) resulted in full conversion of **1a**, affording **3aa** in 89% yield (Table 1, entry 3). Other photocatalysts, such as Ru(bpy)_3_Cl_2_ (**9**) [46], fluorescein (**10**) [48], and phenoxazine **11** [49], were not optimal for the present transformation (Table 1, entries 4–6). It should be noted that the reaction without the photocatalyst under visible light- and halogen lamp irradiation resulted in poorer conversion with formation of **3aa** in only 10% and 30% yield, respectively, suggesting that the photocatalyst was important for the degenerative transfer of xanthate **1a** (Table 1, entries 7 and 8). On the other hand, the employment of a 365 nm UV lamp in place of the blue LED gave **3aa** in 75% yield, although a slower reaction rate was observed compared to the optimal reaction conditions (Table 1, entry 9).

**Table 1:** Optimization of reaction conditions.^a^

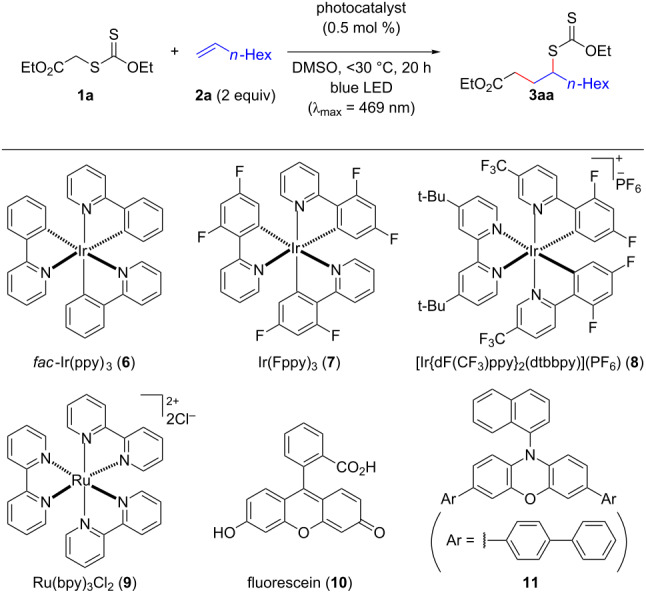					

Entry	Photocat.	*E*_1/2(M+/M*)_ [V vs SCE]^b^	*E*_T_ [kcal/mol]^b^	Conv. [%]^c^	Yield [%]^c^

1	**6**	–1.73	55.2	64	58
2	**7**	–1.28	60.1	38	34
3	**8**	–0.89	60.1	>99	90 (89)^d^
4	**9**	–0.81	46.5	13	13
5	**10**	–1.42	44.7	34	31
6	**11**	–1.80	56.5	9	9
7	none	–	–	10	10
8^e,f^	none	–	–	39	30
9^g^	none	–	–	84	75

^a^The reactions were conducted using 0.3–0.5 mmol of xanthate **1a**, 1-octene (**2a**, 2 equiv) and a photocatalyst in DMSO (1 M) at <30 °C with irradiation of a blue LED strip (λ_max_ = 469 nm, 15 W/m, 1.5 m) under an argon atmosphere. ^b^The values were obtained from references [46–50]. ^c^NMR yields using 1,1,2,2-tetrachloroethane as an internal standard. ^d^Isolated yield is stated in parentheses. ^e^Halogen lamp (300 W) was used in place of blue LED. ^f^Reaction was conducted at 40 °C. ^g^365 nm UV lamp (100 W) was used in place of blue LED.

In principle, visible-light-mediated photocatalysis can serve for electron transfer (for either oxidation or reduction) and/or for energy transfer. We found that the reduction potential *E*_p/2_ of xanthate **1a** is −1.78 V vs SCE, which is not sufficient to be reduced by the photoexcited states of Ir catalysts **6**–**8** (*E*_1/2_ of 6*, 7* and 8* = −1.73, −1.28, and −0.89 V vs SCE, respectively [46–47]). Apparently, photoinduced single-electron reduction of xanthate **1a** by the photoexcited state of the optimal catalyst **8** is not feasible. In contrast, the triplet energy *E*_T_ of xanthate **1a** was estimated as 57.5 kcal/mol by DFT calculation, that is close to those of photocatalysts **7** and **8** (*E*_T_ = 60.1 kcal/mol [50]), indicating that the process could be initiated by the triplet sensitization pathway [51–52]. This assumption is in agreement with the lower process efficiency (Table 1, entry 1) observed in the reaction with *fac*-Ir(ppy)_3_ (**6**) that possesses a lower triplet energy (*E*_T_ = 55.2 kcal/mol [50]). The optimal photocatalyst **8** [47] has a longer excited state lifetime than **7** does [46], suggesting that the lifetime of the excited state of the photocatalyst is a key factor for the energy transfer mechanism.

**Figure 1:**
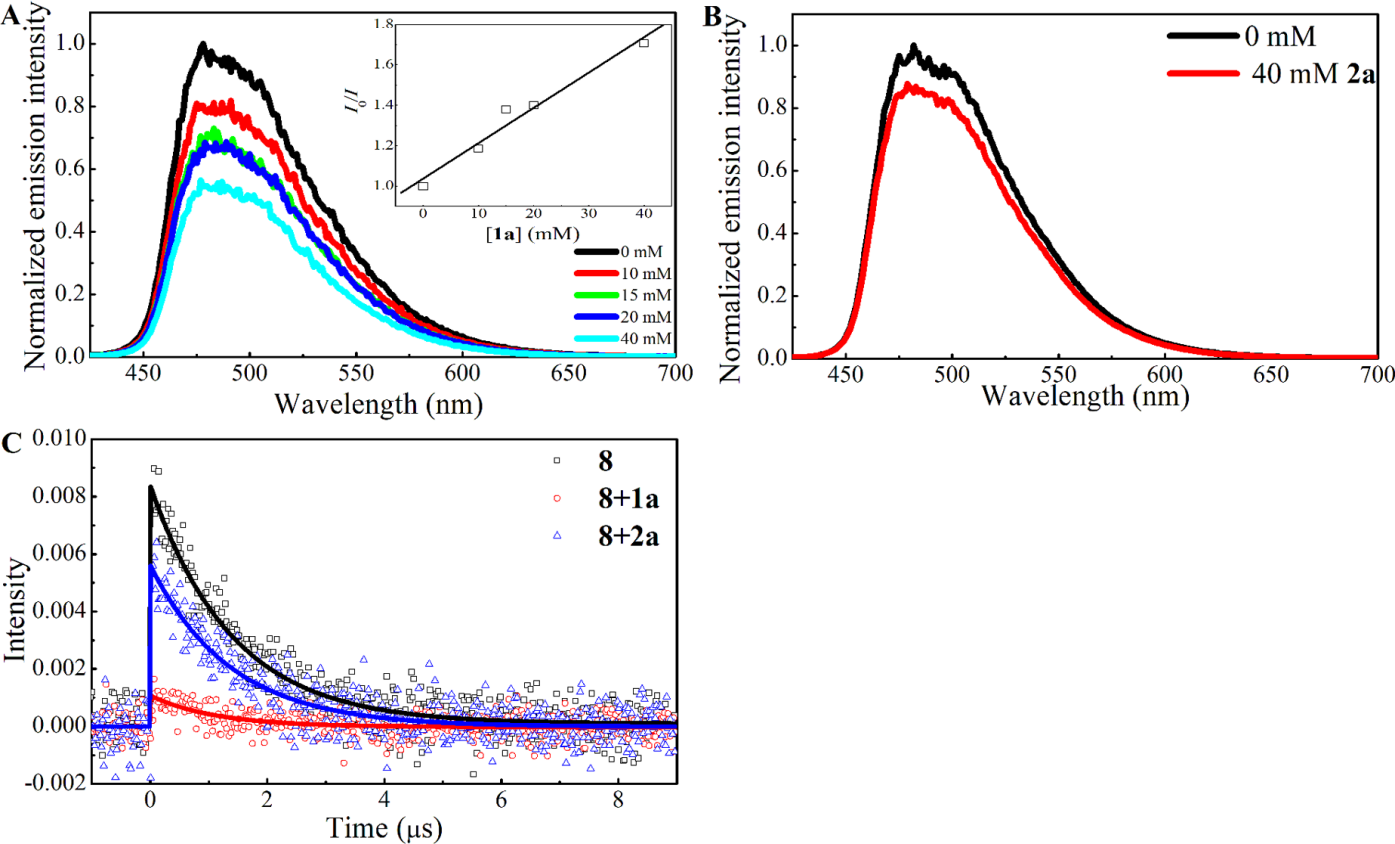
Photoluminescence (PL) spectra of the ^3^MLCT state of **8** in degassed DMSO solvent with (A) various concentrations of xanthate **1a** added and (B) 40 mM of 1-octene (**2a**) added. The inset of (A) gives the Stern–Volmer plot of the corrected PL quenching at 480 nm. (C) Time-resolved PL lifetime decay profiles of photocatalyst **8** in degassed DMSO in the absence of quencher (square), presence of xanthate **1a** (circle) and presence of 1-octene (**2a**, triangle). The mono-exponential decay fits are provided.

To obtain a detailed mechanistic insight, steady-state photoluminescence (PL) quenching of photocatalyst **8** was examined using xanthate **1a** and 1-octene (**2a**) as potential quenchers (Figure 1). The intensity of the PL peak of photocatalyst **8** (concentration of **8** was fixed as 25 μM solution in degassed DMSO for all the samples; for details see Supporting Information File 1) at 480 nm, arising from the radiative emission of the ^3^MLCT state of the photocatalyst, was measured using 410 nm light excitation. When the concentration of xanthate **1a** was gradually increased, a reduction in the PL intensity (*I*) of photocatalyst **8** was observed (Figure 1A). The Stern–Volmer plot of the ratio *I*_0_/*I*, where *I*_0_ is the initial PL intensity in the absence of quencher, versus concentration of **1a** showed a linear relationship with a quenching rate *k*_q_ = 1.25 × 10^7^ M^−1^s^−1^ (see Supporting Information File 1). On the other hand, the addition of 1-octene (**2a**, 40 mM), in place of xanthate **1a**, resulted in only a small PL quenching of photocatalyst **8** (<8%, Figure 1B).

The time-resolved PL lifetime decay profiles of photocatalyst **8** (25 μM solution in degassed DMSO, 410 nm pulse excitation and monitoring emission at 480 nm) were recorded in the absence of a quencher, and in the presence of xanthate **1a** and 1-octene (**2a**, 40 mM, Figure 1C). The lifetime profiles were described using a mono-exponential decay function with a lifetime of 1.40 μs in the absence of a quencher, and 1.03 and 1.37 μs in the presence of xanthate **1a** and 1-octene (**2a**), respectively. The decrease in the PL lifetime of photocatalyst **8** in the presence of xanthate **1a** suggests that they are interacting with each other. On the other hand, only a very weak interaction exists between 1-octene (**2a**) and the photocatalyst **8** as demonstrated by the insignificant PL quenching of the photocatalyst [53].

**Figure 2:**
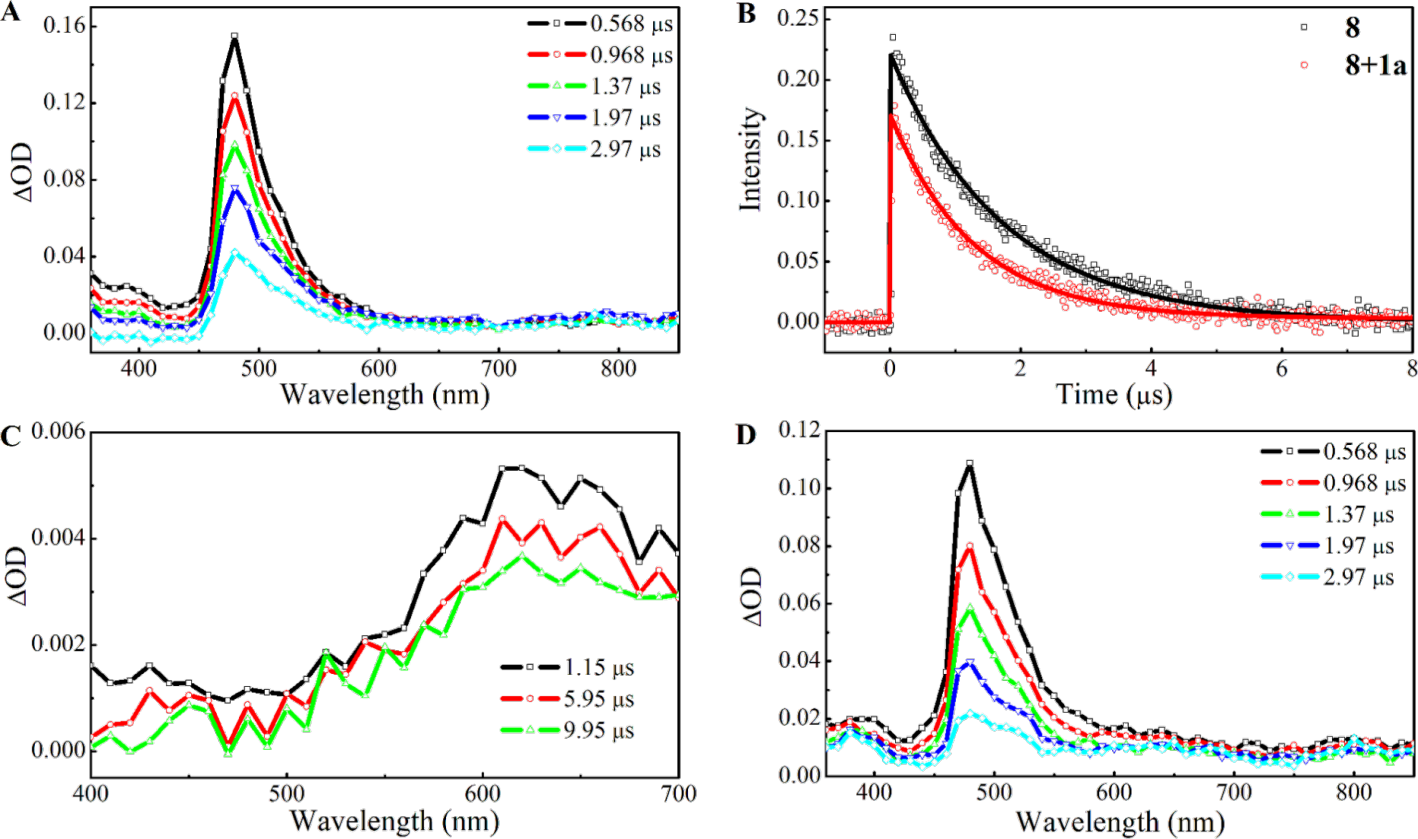
(A) ns-Transient absorption spectra of photocatalyst **8** in degassed DMSO recorded at different delay times (excitation wavelength = 355 nm). (B) ns-TA kinetic profile probed at 480 nm for photocatalyst **8** in the absence and presence of xanthate **1a**. (C) and (D) ns-Transient absorption spectra of xanthate **1a** and **8** in the presence of **1a** in degassed DMSO recorded at different delay times, respectively (excitation wavelength = 355 nm).

The ns-transient absorption (TA) spectra of photocatalyst **8** (25 μM solution in degassed DMSO) obtained using 355 nm pulse excitation and recorded at different delay times are shown in Figure 2A. The band between 450 nm and 600 nm is attributed to the excited ^3^MLCT state of the photocatalyst [53–55]. The positive ΔOD feature in the UV region (<400 nm) is also ascribed to the excited ^3^MLCT state [55]. The transient kinetic profile probed at 480 nm decays mono-exponentially with a lifetime of 1.73 μs (Figure 2B); close to the lifetime of the excited ^3^MLCT state of photocatalyst **8** measured in Figure 1C. The ns-TA spectra of xanthate **1a** in degassed DMSO, measured using 355 nm pulse excitation, at various delay times show a broad band centered at ≈620 nm (Figure 2C). This band has previously been ascribed to the absorption of the xanthic acid radical formed plausibly from homolytic C–S bond cleavage of the short-lived triplet state of xanthate **1a** [22].

The ns-TA spectra of photocatalyst **8** (25 μM) in the presence of xanthate **1a** (40 mM) in degassed DMSO at various delay times are shown in Figure 2D. The kinetic profile at 480 nm is described using a mono-exponential decay function with a quenched lifetime of 1.27 μs. The ca. 37% decrease in the lifetime of the excited ^3^MLCT state of photocatalyst **8** observed in the PL lifetime decay measurement (Figure 1C) and ns-TA kinetic measurement (Figure 2B) can be rationalized using either an energy-transfer or an electron-transfer mechanism. For the same delay times and in the presence of **1a**, the ΔOD values in Figure 2D are smaller than those of photocatalyst **8** in the absence of the xanthate (Figure 2A). This is due to the quenching of the ^3^MLCT state of the photocatalyst. If an electron-transfer process occurs from photocatalyst **8** to xanthate **1a**, the ΔOD values in the UV region should be noticeably higher due to the TA band contribution from bpy^−^ connected to an Ir metal center of the +4 oxidation state (i.e., absorption due to [Ir^IV^{dF(CF_3_)ppy}_2_](dtbpy^−^)] species) [55]. However, this was not observed in Figure 2D; suggesting that quenching is due to energy transfer rather than electron transfer, and in agreement with the thermodynamic consideration where single-electron reduction of xanthate **1a** likely does not proceed with the excited photocatalyst **8**. We therefore propose that the observed PL quenching is due to energy transfer from the excited ^3^MLCT state of photocatalyst **8** to the triplet state of xanthate **1a**. When comparing the normalized TA spectra of photocatalyst **8** in the absence and presence of xanthate **1a** (see Supporting Information File 1, Figure S4), an additional contribution from a broad ΔOD band that stretches from 500 nm to 800 nm is seen for the latter which is attributed to the absorption of the xanthic acid radical. In this case, the xanthic acid radical is formed from the homolytic bond cleavage of the excited triplet state of **1a** formed by direct 355 nm laser light excitation and triplet–triplet energy transfer involving the excited photocatalyst **8**.

**Figure 3:**
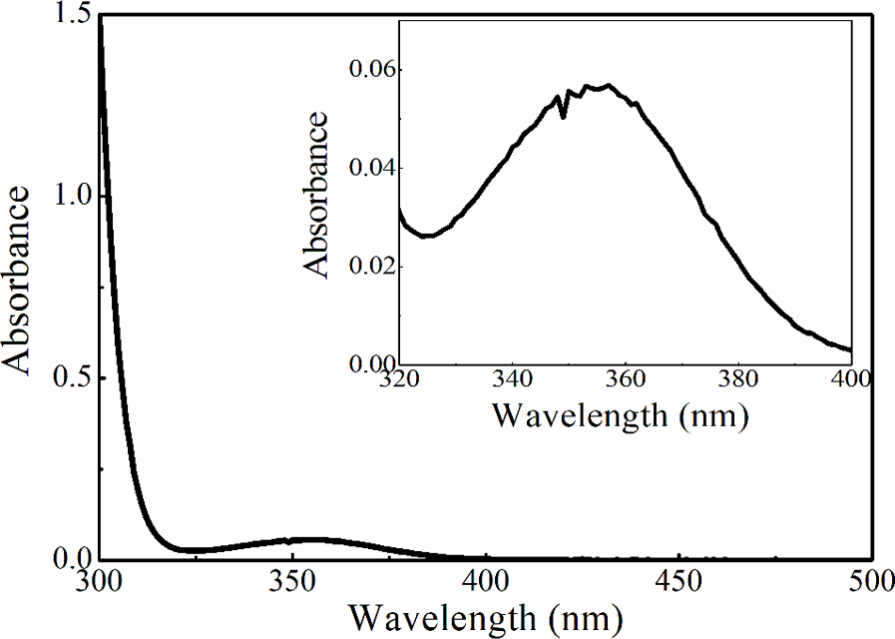
UV–vis absorption spectrum of **1a** (1 mM solution in DMSO).

To confirm the possibility of a direct photoexcitation of xanthate **1** using blue LED light irradiation as an alternative mechanism, a steady-state UV–vis absorption spectroscopy study of xanthate **1a** was conducted (Figure 3). The UV–vis absorption spectrum of **1a** (1 mM in DMSO) showed absorption bands at 340–390 nm assigned to the n→π* electronic transition of the C=S bond as a characteristic peak of thiocarbonyl containing compounds [56]. In fact, the reaction of **1a** and **2a** under 365 nm UV lamp irradiation without a photocatalyst delivered product **3aa** in 75% yield (Table 1, entry 9). This indicates the excitation of xanthate **1a** through an n→π* electronic transition of the C=S bond is in operation in the UV irradiation process.

**Scheme 3:**
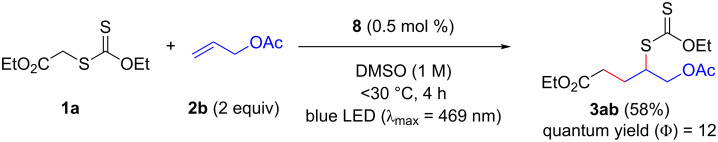
Determination of quantum yield.

The involvement of a radical chain mechanism was further confirmed by calculating the quantum yield (Φ) because a chain process provides multiple equivalents of product per photon absorbed by the photocatalyst (Φ > 1). The photon flux of blue LED (λ_max_ = 469 nm) was determined using the potassium ferrioxalate actinometer [57–58]. After irradiation of the mixture of xanthate **1a** and olefin **2b** under optimal reaction conditions with blue LED light irradiation for 4 h (Scheme 3), product **3ab** was obtained in 58% yield. This is consistent with 12 equivalents of xanthate adduct **3ab** produced per photon absorbed by the photocatalyst **8** (Φ = 12).

**Scheme 4:**
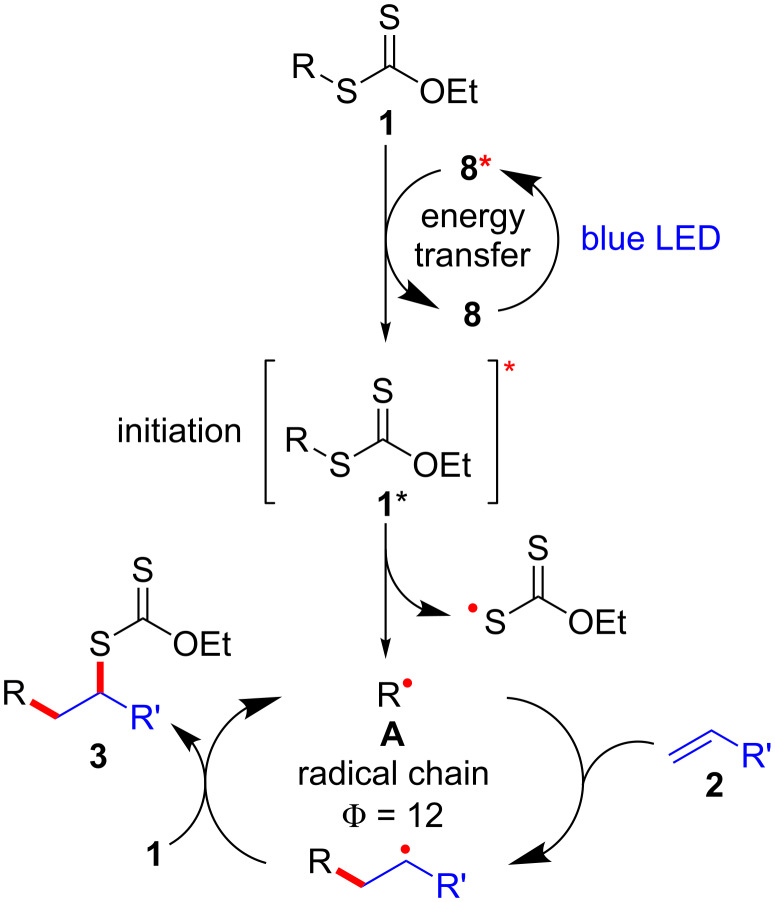
Proposed reaction mechanism.

On the basis of these observations, a proposed triplet sensitization mechanism is illustrated in Scheme 4. In this reaction, photocatalyst **8** serves as a catalyst of an initiation step through energy transfer from photoexcited 8* to xanthate **1** to form excited xanthate 1* and regeneration of **8** in the ground state [59–61]. The resulting excited xanthate 1* induces homolytic scission of the C–S bond to generate the stabilized S-radical and C-radical **A**, which then enters the innate radical chain-propagation mechanism to provide xanthate adduct **3**. It is worth noting that at the wavelength of the light source used (469 nm), xanthate **1a** absorbs a negligible amount of light (Figure 3) and the majority of triplet **1a** formed is due to energy transfer from excited catalyst 8*.

Having optimized the reaction conditions on the photocatalytic degenerative transfer of xanthates, we next explored the scope of olefins **2** using xanthate **1a** (Table 2). The present method tolerated a variety of functionalities such as acetyl, cyano, silyl, ethoxy, *N*-Boc amino, boryl, hydroxy, and halogen groups, affording xanthate adducts **3ab**–**ai** in good yields (Table 2, entries 1–8). We found strained 1,1-disubstituted olefins such as methylenecyclopropane **2j** and methylenecyclobutane (**2k**) are amenable to the current protocol (Table 2, entries 9 and 10), whereas the reaction of methylenecyclopentane (**2l**) afforded not only the desired xanthate adduct **3al** in 60% yield but also the substituted cyclopentene **3al’** in 18% yield (Table 2, entry 11), implicating that the redox process is partially operating along with the main radical chain process. Norbornene (**2m**) was found reactive for degenerative transfer of xanthate **1a** (Table 2, entry 12). The reaction was also applied to dienes **2n** and **2o**, which led the formation of functionalized cyclopentane **3an** and pyrrolidine **3ao**, respectively, via 5-*exo*-trig radical cyclization (Table 2, entries 13 and 14). The present method was capable in functionalizing olefins **2p** and **2q** installed on steroid scaffolds with high efficiency (Table 2, entries 15 and 16).

**Table 2:** Scope of olefins.^a^

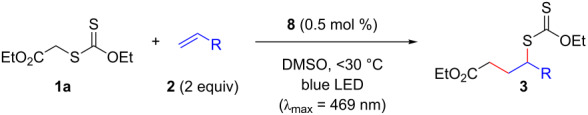				

Entry	Olefins **2**		Products **3**	Yield^b^ (time)

	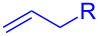 **2**	R	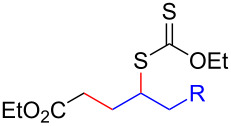	

1	**2b**	OAc	**3ab**	88% (18 h)
2	**2c**	CN	**3ac**	73% (45 h)
3	**2d**	SiMe_3_	**3ad**	85% (46 h)
4^c^	**2e**	OEt	**3ae**	70% (41 h)
5^c^	**2f**	NHBoc	**3af**	80% (61 h)
6^d^	**2g**	Bpin	**3ag**	72% (30 h)

7	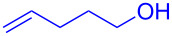 **2h**		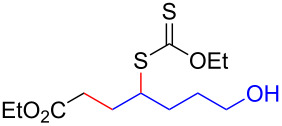 **3ah**	84% (37 h)

8	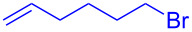 **2i**		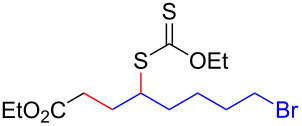 **3ai**	73% (48 h)

9	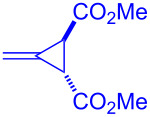 **2j**		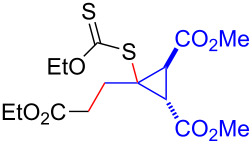 **3aj**	64% (48 h)

10	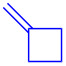 **2k**		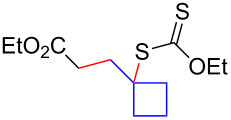 **3ak**	90% (26 h)

11^d^	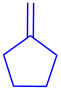 **2l**		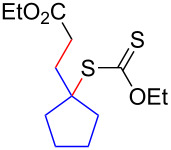 **3al**	60% (20 h)
			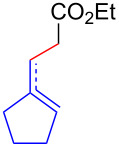 **3al’**	18%^e^ (20 h) *endo*/*exo* = 80:20

12	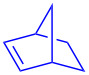 **2m**		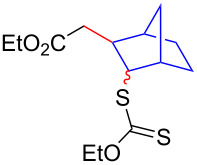 **3am**	73% (10 h) (dr = 78:22)

13	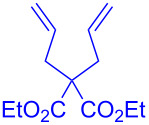 **2n**		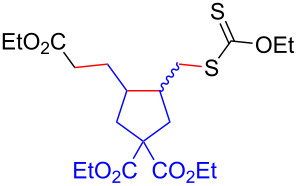 **3an**	68% (24 h) (dr = 88:12)

14	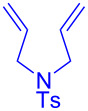 **2o**		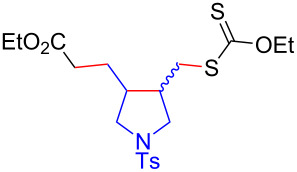 **3ao**	74% (13 h) (dr = 71:29)

15^f^	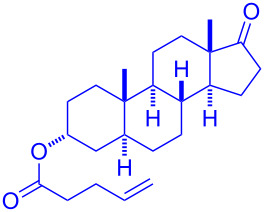 **2p**		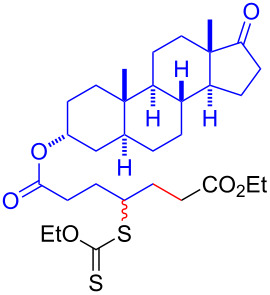 **3ap**	94% (26 h) (dr = 50:50)

16^g^	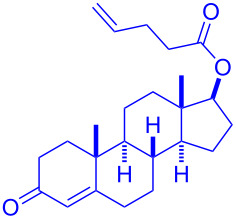 **2q**		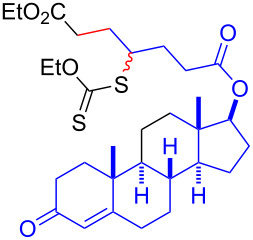 **3aq**	80% (50 h) (dr = 50:50)

^a^The reactions were conducted using xanthate **1a** (0.3–0.5 mmol), olefin **2** (2 equiv) and **8** (0.5 mol %) in DMSO (1 M) at <30 °C with irradiation of a blue LED strip (λ_max_ = 469 nm) under an argon atmosphere. ^b^Isolated yields are stated. ^c^1 mol % of **8** was used. ^d^4 equiv of olefin **2** were used. ^e^NMR yield using 1,1,2,2-tetrachloroethane as an internal standard. ^f^The reaction was conducted in DMSO/DCE 1:1 (0.5 M). ^g^The reaction was conducted in DMSO/DCE 3:5 (0.4 M).

We next examined the reactions of various xanthates **1** with allyl acetate (**2b**, Table 3). The reactions of ketonyl xanthates having phenyl, *para*-bromophenyl, methyl, cyclopropyl, *N*,*O*-dimethyl acetylhydroxamate, and chloromethyl moieties proceeded smoothly, producing xanthate adducts **3bb**–**gb** in good yields (Table 3, entries 1–6). Notably, the photocatalytically cleavable aryl bromide (Table 3, entry 2) and the α-chlorocarbonyl moiety (Table 3, entry 6) were also stable under the current reaction conditions [62–63]. Furthermore, double addition of bisxanthate **1h** was successfully achieved in the presence of 5 equiv of olefin **2b**, giving **3hb** in 69% yield (Table 3, entry 7). This method is also suitable for generating α-aminoalkyl radicals from phthalimidomethyl and succinimidomethyl xanthates [64], as well as α-trifluoromethylamino xanthate **1k** [65] to afford desired products **3ib**–**kb** in good to moderate yields (Table 3, entries 8–10).

**Table 3:** Scope of xanthates.^a^

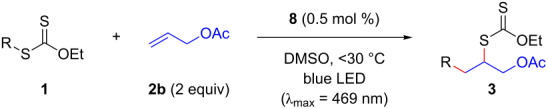			

Entry	Xanthates **1**	Products **3**	Yield^b^ (time)

1	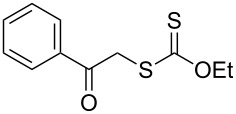 **1b**	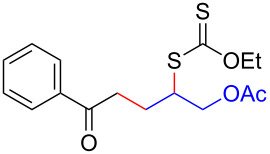 **3bb**	78% (44 h)
2	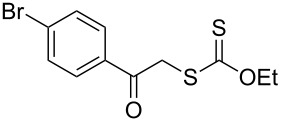 **1c**	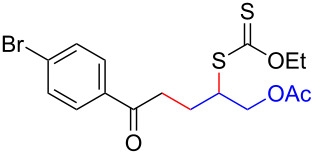 **3cb**	69% (47 h)
3	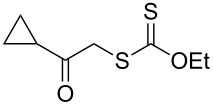 **1d**	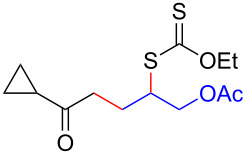 **3db**	59% (51 h)
4^c^	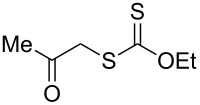 **1e**	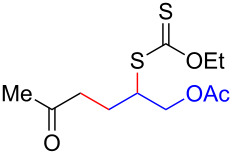 **3eb**	82% (71 h)
5	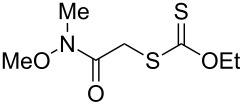 **1f**	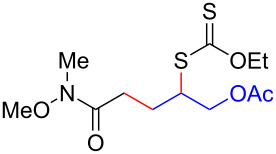 **3fb**	81% (27 h)
6	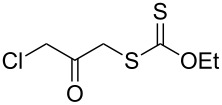 **1g**	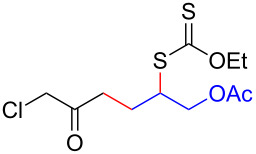 **3gb**	75% (17 h)
7^d^	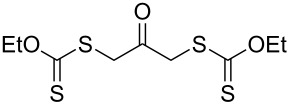 **1h**	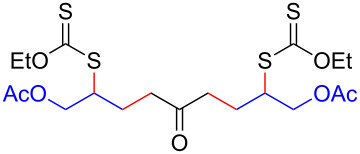 **3hb**	69% (52 h) (dr = 52:48)
8	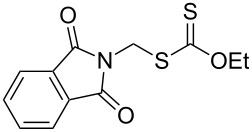 **1i**	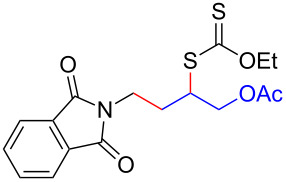 **3ib**	80% (41 h)
9	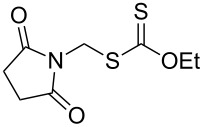 **1j**	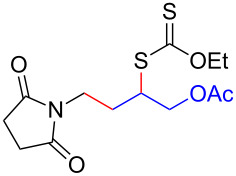 **3jb**	56% (24 h)
10	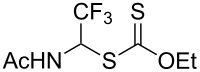 **1k**	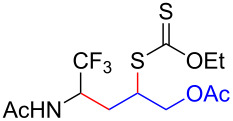 **3kb**	74% (24 h) (dr = 63:37)

^a^The reactions were conducted using xanthate **1** (0.3 mmol), olefin **2b** (2 equiv) and **8** (0.5 mol %) in DMSO (1 M) at <30 °C with irradiation of a blue LED strip (λ_max_ = 469 nm) under an argon atmosphere. ^b^Isolated yields are stated. ^c^1 mol % of **8** was used. ^d^Five equivalents of olefin **2b** were used.

## Conclusion

We have established a protocol for a photoinduced radical addition of xanthates to olefins using an iridium-based photocatalyst under blue LED irradiation, leading to diverse xanthate adducts. This reaction proceeds through a radical-chain propagation mechanism via an initiation involving a triplet-sensitization process of xanthates by an excited iridium-based photocatalyst.

## Supporting Information

File 1 Full experimental details and analytical data.

## References

[1] Zard S Z (2018). Acc Chem Res.

[2] Quiclet-Sire B, Zard S Z (2017). Synlett.

[3] Quiclet-Sire B, Zard S Z (2017). Isr J Chem.

[4] Zard S Z (2016). Org Biomol Chem.

[5] Quiclet-Sire B, Zard S Z (2016). Synlett.

[6] Debien L, Quiclet-Sire B, Zard S Z (2015). Acc Chem Res.

[7] Zard S Z (2012). J Phys Org Chem.

[8] Quiclet-Sire B, Zard S Z (2012). Chimia.

[9] Quiclet-Sire B, Zard S Z (2011). Pure Appl Chem.

[10] Quiclet-Sire B, Zard S Z (2006). Top Curr Chem.

[11] Quiclet-Sire B, Zard S Z (2006). Chem – Eur J.

[12] Zard S Z (2006). Aust J Chem.

[13] Zard S Z (1997). Angew Chem, Int Ed Engl.

[14] Czaplyski W L, Na C G, Alexanian E J (2016). J Am Chem Soc.

[15] Perrier S (2017). Macromolecules.

[16] Chiefari J, Chong Y K, Ercole F, Krstina J, Jeffery J, Le T P T, Mayadunne R T A, Meijs G F, Moad C L, Moad G (1998). Macromolecules.

[17] García-Merinos J P, Hernández-Pérez J P, Martínez-García L, Rojas-Lima S, López-Ruiz H (2007). J Mex Chem Soc.

[18] Boivin J, Nguyen V T (2007). Beilstein J Org Chem.

[19] Charrier N, Gravestock D, Zard S Z (2006). Angew Chem, Int Ed.

[20] Jean-Baptiste L, Yemets S, Legay R, Lequeux T (2006). J Org Chem.

[21] Briggs M E, Zard S Z (2005). Synlett.

[22] Tazhe Veetil A, Šolomek T, Ngoy B P, Pavlíková N, Heger D, Klán P (2011). J Org Chem.

[23] Ferjančić Z, Čeković Ž, Saičić R N (2000). Tetrahedron Lett.

[24] Maslak V, Čeković Ž, Saičić R N (1998). Synlett.

[25] Mestre F, Tailham C, Zard S Z (1989). Heterocycles.

[26] Delduc P, Tailhan C, Zard S Z (1988). J Chem Soc, Chem Commun.

[27] López-Mendoza P, Díaz J E, Loaiza A E, Miranda L D (2018). Tetrahedron.

[28] Twilton J, Le C, Zhang P, Shaw M H, Evans R W, MacMillan D W C (2017). Nat Rev Chem.

[29] Cambié D, Bottecchia C, Straathof N J W, Hessel V, Noël T (2016). Chem Rev.

[30] Romero N A, Nicewicz D A (2016). Chem Rev.

[31] Skubi K L, Blum T R, Yoon T P (2016). Chem Rev.

[32] Ravelli D, Protti S, Fagnoni M (2016). Chem Rev.

[33] Kärkäs M D, Porco J A, Stephenson C R J (2016). Chem Rev.

[34] Shaw M H, Twilton J, MacMillan D W C (2016). J Org Chem.

[35] Corrigan N, Shanmugam S, Xu J, Boyer C (2016). Chem Soc Rev.

[36] Prier C K, Rankic D A, MacMillan D W C (2013). Chem Rev.

[37] Narayanam J M R, Stephenson C R J (2011). Chem Soc Rev.

[38] Ding C, Fan C, Jiang G, Pan X, Zhang Z, Zhu J, Zhu X (2015). Macromol Rapid Commun.

[39] Shanmugam S, Xu J, Boyer C (2014). Macromolecules.

[40] Xu J, Jung K, Atme A, Shanmugam S, Boyer C (2014). J Am Chem Soc.

[41] Phommalysack-Lovan J, Chu Y, Boyer C, Xu J (2018). Chem Commun.

[42] Huang Z, Noble B B, Corrigan N, Chu Y, Satoh K, Thomas D S, Hawker C J, Moad G, Kamigaito M, Coote M L (2018). J Am Chem Soc.

[43] Aerts A, Lewis R W, Zhou Y, Malic N, Moad G, Postma A (2018). Macromol Rapid Commun.

[44] Fu C, Huang Z, Hawker C J, Moad G, Xu J, Boyer C (2017). Polym Chem.

[45] Xu J, Fu C, Shanmugam S, Hawker C J, Moad G, Boyer C (2017). Angew Chem, Int Ed.

[46] Teegardin K, Day J I, Chan J, Weaver J (2016). Org Process Res Dev.

[47] Lowry M S, Goldsmith J I, Slinker J D, Rohl R, Pascal R A, Malliaras G G, Bernhard S (2005). Chem Mater.

[48] Shen T, Zhao Z-G, Yu Q, Xu H-J (1989). J Photochem Photobiol, A.

[49] Du Y, Pearson R M, Lim C-H, Sartor S M, Ryan M D, Yang H, Damrauer N H, Miyake G M (2017). Chem – Eur J.

[50] Singh A, Teegardin K, Kelly M, Prasad K S, Krishnan S, Weaver J D (2015). J Organomet Chem.

[51] Strieth-Kalthoff F, James M J, Teders M, Pitzer L, Glorius F (2018). Chem Soc Rev.

[52] Xiao W-J, Zhou Q-Q, Zou Y-Q, Lu L-Q (2018). Angew Chem, Int Ed.

[53] Teders M, Henkel C, Anhäuser L, Strieth-Kalthoff F, Gómez-Suárez A, Kleinmans R, Kahnt A, Rentmeister A, Guldi D, Glorius F (2018). Nat Chem.

[54] van As D J, Connell T U, Brzozowski M, Scully A D, Polyzos A (2018). Org Lett.

[55] Ichimura K, Kobayashi T, King K A, Watts R J (1987). J Phys Chem.

[56] Coyle J D (1985). Tetrahedron.

[57] Cismesia M A, Yoon T P (2015). Chem Sci.

[58] Hatchard C G, Parker C A (1956). Proc R Soc London, Ser A.

[59] Studer A, Curran D P (2016). Angew Chem, Int Ed.

[60] Christmann J, Ibrahim A, Charlot V, Croutxé-Barghorn C, Ley C, Allonas X (2016). ChemPhysChem.

[61] Arceo E, Montroni E, Melchiorre P (2014). Angew Chem, Int Ed.

[62] Devery J J, Nguyen J D, Dai C, Stephenson C R J (2016). ACS Catal.

[63] Kim H, Lee C (2012). Angew Chem, Int Ed.

[64] Quiclet-Sire B, Zard S Z (2008). Org Lett.

[65] Gagosz F, Zard S Z (2003). Org Lett.

