# Supplementary material for: Degenerative xanthate transfer to olefins under visible-light photocatalysis
Source: Beilstein J Org Chem. 2018 Dec 13;14:3047–58. doi: 10.3762/bjoc.14.283 (PMC6296426; doi:10.3762/bjoc.14.283)

BEILSTEIN JOURNAL OF ORGANIC CHEMISTRY

# Supporting Information

for

# Degenerative xanthate transfer to olefins under visible-light photocatalysis

Atsushi Kaga, Xiangyang Wu, Joel Yi Jie Lim, Hirohito Hayashi, Yunpeng Lu, Edwin K. L. Yeow and Shunsuke Chiba

*Beilstein J. Org. Chem.* **2018,** *14,* 3047–3058. doi:10.3762/bjoc.14.283

# Full experimental details and analytical data

License and Terms: This is a supporting information file under the terms of the Creative Commons Attribution License (http://creativecommons.org/licenses/by/4.0). Please note that the reuse, redistribution and reproduction in particular requires that the authors and source are credited.
The license is subject to the *Beilstein Journal of Organic Chemistry* terms and conditions: (https://www.beilstein-journals.org/bjoc)

**Table of Contents**


1. **General** **S3**
2. **Emission spectrum of employed blue LED** **S3**
3. **Cyclic voltammetry** **S4**
4. **Stern-Volmer emission quenching** **S5**
5. **Time-resolved photoluminescence lifetime decay profile of 8** **S6**
6. **Transient absorption spectroscopy** **S7**
7. **Determination of quantum yield** **S8**
8. **Synthesis of starting materials** **S12**
9. **Photocatalytic degenerative xanthate transfer to olefins** **S15**
10. **$^{1}$H and $^{13}$C NMR spectra for new compounds** **S33**

## 1. General

$^{1}H$ NMR spectra were recorded on a Bruker Avance 400, 500, or JEOL ECA400SL spectrometer in $CDCl_3$ [using TMS (for $^{1}H$, $\delta$ = 0.00) as internal standard] or $C_6D_6$ [using $C_6D_6$ (for $^{1}H$, $\delta$ = 7.16) as internal standard]. $^{13}C$ NMR spectra were recorded on a Bruker Avance 400, 500, or JEOL ECA400SL spectrometer in $CDCl_3$ [using $CDCl_3$ (for $^{13}C$, $\delta$ = 77.00) as internal standard] or $C_6D_6$ [using $C_6D_6$ (for $^{13}C$, $\delta$ = 128.06) as internal standard]. The following abbreviations were used to explain the multiplicities: s = singlet, d = doublet, t = triplet, q = quartet, br = broad. High-resolution mass spectra were obtained with a Waters Q-Tof Premier mass spectrometer. Melting points are uncorrected and were recorded on a MPA 100 OptiMelt Automated Melting Point System. Flash chromatography was performed using Merck silica gel 60 with distilled solvents. The blue LED strip (1.5 m, 15 W/m, $\lambda_{max}$ = 469 nm) was purchased from Leomay Technology Co., Ltd (http://leomay.com/upload/file/LM5730-60D-20161119093733.pdf) and attached on a crystallizing dish (diameter 115 mm). The Analytic Jena high intensity UV lamp B-100AP/R ($\lambda$ = 365 nm, 100 W) was employed for the control experiment (Table 1, entry 8). Theoretical calculation on xanthate **1a** was performed with Gaussian 09 (Rev. E.01) programs. Geometry optimization was conducted by B3LYP functional. The Def2-SVP basis set was employed for the elements H, C, N, O and Def2-TZVP basis set was used for S. To calculate the excitation energy, the first triplet excited state geometry was optimized with the same method. $[Ir\{dF(CF_3)ppy\}_2(dtbbpy)](PF_6)$ (**8**) (CAS: 870987-63-6) was purchased from Sigma-Aldrich and used as received.

## 2. Emission spectrum of employed blue LED

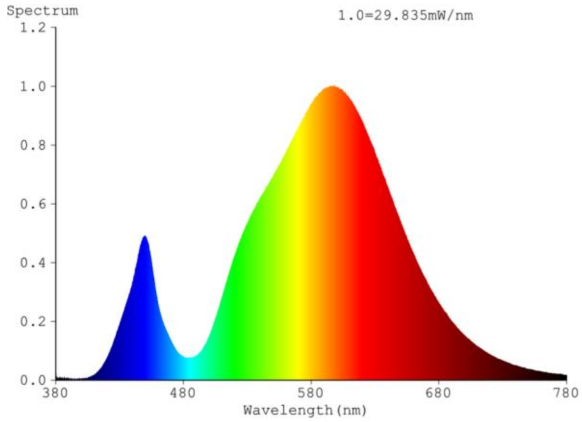


**Figure S1:** Emission spectrum of blue LED obtained from Leomay Technology Co., Ltd (http://leomay.com/upload/file/LM5730-60D-20161119093733.pdf).

## 3. Cyclic voltammetry

The cyclic voltammetry experiments were conducted with a computer-controlled Eco Chemie Autolab PGSTAT302N potentiostat in a three-electrode cell. All electrochemical measurements were carried out under an argon atmosphere and the samples were bubbled with argon before measurement. Cyclic voltammograms of 1.2 mM xanthate **1a** with 0.1 M $n$-$Bu_4NPF_6$ as the supporting electrolyte were recorded at a scan rate of 100 mV/s with a planar glassy carbon disc (1 mm diameter) as working electrode, a platinum wire as counter electrode and an Ag wire (in 0.5 M $n$-$Bu_4NPF_6$ in MeCN) as a pseudo-reference electrode at 298 ± 2 K. Ferrocene was added to the sample solution as an internal reference (the ferrocene/ferrocenium couple appeared at $E_{1/2}$ (Fc/Fc$^+$) = 0.583 V vs. Ag wire). Using this ferrocene redox couple, the potential $E$ vs. SCE (saturated calomel electrode) was calculated by the following equation, based on $E_{1/2}$ (Fc/Fc$^+$) = 0.400 V vs. SCE [1]:

$$E\ (vs. SCE) = E\ (vs. Ag\ wire) - 0.183\ V$$

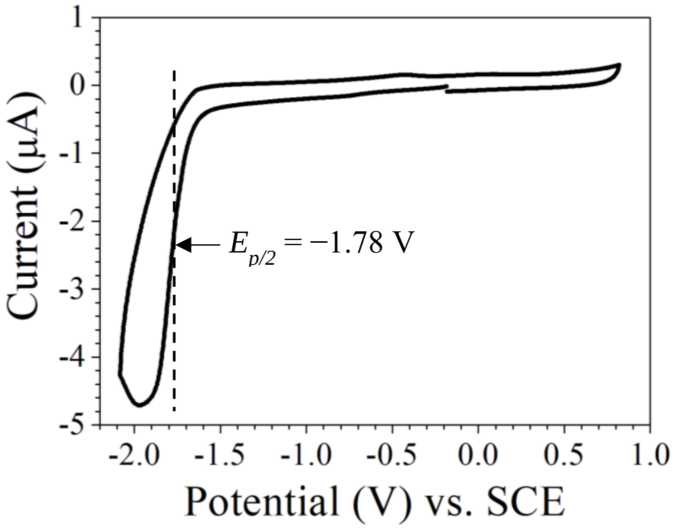


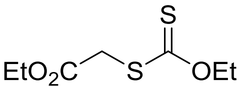


**1a**

**Figure S2:** Cyclic voltammogram of 1.2 mM xanthate **1a** in MeCN.

Peak potential of xanthate **1a**: $E_p$ = –1.97 V vs. SCE

Half-peak potential of xanthate **1a**: $E_{p/2}$ = –1.78 V vs. SCE

1. Dong, T.-Y.; Hwang, M.-Y.; Hsu, T.-L.; Schei, C.-C.; Yeh, S.-K. *Inorg. Chem.* **1990**, *29*, 80−84.

## 4. Stern–Volmer emission quenching

Emission intensities were measured using a fluorescence spectrometer (Eclipse, Varian). All samples were excited at 410 nm and the emission intensities were collected at 480 nm. In a typical experiment, to a 25 μM solution of [Ir{dF($CF_3$)ppy}$_2$(dtbbpy)]($PF_6$) (**8**) in DMSO was added the appropriate amount of a quencher in a screw-top cuvette to obtain a total volume of 2.0 mL. After degassing the sample with bubbling of argon for 15 minutes, the emission of the sample was collected.

Because both **1a** and **2a** can absorb light at 410 nm, it is necessary to do the correction so that the photocatalyst **8** in the presence of **1a** or **2a** and **8** alone absorb equal incident light intensity [2]. The fraction of intensity of the light absorbed by the photocatalyst **8** ($F_{\mathbf{8}}$) in the systems of **8**/**1a** and **8**/**2a** is obtained using the following equation:

$$F_{\mathbf{8}} = \frac{A_{\mathbf{8}}}{A_{\mathbf{8}} + A_{\mathbf{1a/2a}}}\left(1 - 10^{-(A_{\mathbf{8}} + A_{\mathbf{1a/2a}})}\right)$$

where $A_{\mathbf{8}}$ and $A_{\mathbf{1a/2a}}$ are the absorbances of **8** and **1a** (or **2a**) at 410 nm.

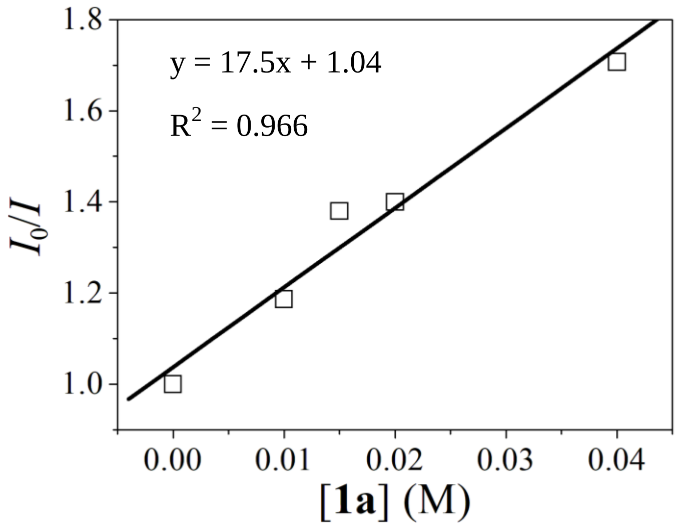


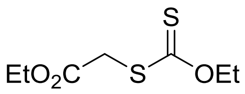


**1a**

**Figure S3:** Stern–Volmer quenching experiment of [Ir{dF($CF_3$)ppy}$_2$(dtbbpy)]($PF_6$) (**8**) (25 μM in DMSO) with xanthate **1a**. Average of two runs.

2. Yeow, E. K. L.; Ziolek, M.; Karolczak, J.; Shevyakov, S. V.; Asato, A. E.; Maciejewski, A.; Steer, R. P. *J. Phys. Chem. A* **2004**, *108*, 10980–10988.

The quenching rate ($k_q$) was determined using Stern–Volmer kinetics.

$$\frac{I_0}{I} = k_q \cdot \tau \cdot [\text{quencher}]$$

Where $I_0$ is the luminescence intensity without the quencher, $I$ is the intensity with the quencher, and τ is the lifetime of [Ir{dF($CF_3$)ppy}$_2$(dtbbpy)]($PF_6$) (**8**) at 410 nm (1.40 μs, see section 5). The quenching rate ($k_q$) of xanthate **1a** is determined to be $1.25 \times 10^7$ $M^{-1}s^{-1}$.

$$k_q = \frac{slope}{\tau} = \frac{17.5\ M^{-1}}{1.40 \cdot 10^{-6}\ s} = 1.25 \cdot 10^7\ M^{-1}s^{-1}$$

## 5. Time-resolved photoluminescence lifetime decay profile of 8

The excited-state lifetimes of sole [Ir{dF($CF_3$)ppy}$_2$(dtbbpy)]($PF_6$) (**8**), and **8** with xanthate **1a** or 1-octene (**2a**) were measured using a laser flash photolysis spectrometer (LKS.60, Applied Photophysics), equipped with a Q-Switched Nd:YAG laser (Brilliant B, Quantel) and a R928 photomultiplier. Samples were excited at 410 nm (for Figure 1C) or 355 nm (for Figure 2B) and each time-resolved emission decay curves were acquired at 480 nm. In a typical experiment, to a 25 μM solution of [Ir{dF($CF_3$)ppy}$_2$(dtbbpy)]($PF_6$) (**8**) in DMSO was added a quencher (40 mM) in a screw-top cuvette to obtain a total volume of 2.0 mL. After degassing the sample with bubbling of argon for 15 min, the photoluminescence lifetime of the sample was collected.

***Photoluminescence lifetime in DMSO (Excitation at 410 nm)*** **[for Figure 1C]**

**8** (25 μM): 1.40 μs

**8** (25 μM) + xanthate **1a** (40 mM): 1.03 μs

**8** (25 μM) + 1-octene (**2a**) (40 mM): 1.37 μs

***Photoluminescence lifetime in DMSO (Excitation at 355 nm)*** **[for Figure 2B]**

**8** (25 μM): 1.73 μs

**8** (25 μM) + xanthate **1a** (40 mM): 1.27 μs

## 6. Transient absorption spectroscopy

ns-Transient absorption spectroscopy measurements were performed using a laser flash photolysis spectrometer (LKS.60, Applied Photophysics), equipped with a Q-Switched Nd:YAG laser (Brilliant B, Quantel), a 150 W Xe lamp and a R928 photomultiplier, to record ns-difference absorption (ΔOD) spectra. Samples were excited at 355 nm and each time-resolved trace was acquired by averaging 20 laser shots at a repetition rate of 1 Hz. In a typical experiment, to a 25 μM solution of [Ir{dF($CF_3$)ppy}$_2$(dtbbpy)]($PF_6$) (**8**) in DMSO was added a quencher (40 mM) in a screw-top cuvette to obtain a total volume of 2.0 mL. After degassing the sample with bubbling of argon for 15 min, a transient absorption spectrum was measured. For the transient absorption spectroscopy of **1a** alone (for Figure 2C), a 10 mM solution of **1a** (total volume: 2.0 mL) was prepared.

As shown in Figure S4, when comparing the normalized TA spectra of photocatalyst **8** in the absence (Figure 2A) and presence of xanthate **1a** (Figure 2D), an additional contribution from a broad ΔOD band that stretches from 500 nm to 800 nm is seen for the latter which is attribute to absorption of the xanthic acid radical. In this case, the xanthic acid radical is formed from the homolytic bond cleavage of the excited triplet state of **1a** formed by direct 355 nm laser light excitation and triplet-triplet energy transfer involving the excited photocatalyst **8**.

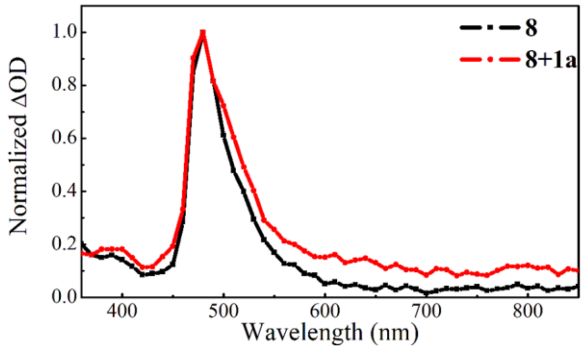


**Figure S4:** Normalized ns-TA spectra of photocatalyst **8** in the absence and presence of xanthate **1a** in degassed DMSO.

## 7. Determination of quantum yield

We utilized the protocol reported by Yoon and co-workers to determine the photon flux of the blue LED [3]. All solutions were stored in the dark when not in use. Measurements were performed with the lights off to protect the samples from ambient light as much as possible. Potassium ferrioxalate trihydrate, $[K_3Fe^{III}(C_2O_4)_3]•3H_2O$ (CAS: 5936-11-8) was recrystallized three times from $H_2O$. 1,10-Phenanthroline (CAS: 5144-89-8) was recrystallized once from benzene.

***Preparation of stock solutions***

A 0.15 M solution of ferrioxalate was obtained by dissolving potassium ferrioxalate trihydrate ($[K_3Fe^{III}(C_2O_4)_3]•3H_2O$; 1.11 g, 2.26 mmol) in 0.05 M $H_2SO_4$ (prepared by fresh deionized water) (15 mL total volume).

A buffered phenanthroline solution was obtained by dissolving 1,10-phenanthroline (10.0 mg) and sodium acetate (2.25 g) in 0.5 M $H_2SO_4$ (prepared by fresh deionized water) (10 mL total volume).

***Determination of background $Fe^{2+}$ concentration***

3 mL of the ferrioxalate solution was added to a 4 mL vial. Next, 0.525 mL of the phenanthroline solution was added and the mixture was stored in the dark for 1 hour. Then the solution was transferred to a cuvette and a UV–vis spectrum was measured using UV–vis absorption spectrometer (Cary 100, Varian). The absorbance value at 510 nm was recorded. This process was repeated twice.

Average value: 0.523766855

***Determination of photon flux***

Three mL of the ferrioxalate solution was added to a 4 mL vial. The vial was immediately irradiated with blue LED ($\lambda_{max}$ = 469 nm) for 90 seconds and removed from the blue LED. Then, 0.525 mL of the phenanthroline solution was added to the ferrioxalate solution, and the resulting mixture was stored in the dark for 1 hour. Then the solution was transferred to a cuvette and the UV–vis spectrum was measured. The absorbance value at 510 nm was recorded. This process was repeated twice.

Average value: 2.045735439

---

3. Cismesia, M. A.; Yoon, T. P. *Chem. Sci.* **2015**, *6*, 5426–5434.

***Calculations***

The amount of $Fe^{2+}$ formed was calculated according to the following equation:

$$\text{mol Fe}^{2+} = \frac{V \cdot \Delta A}{l \cdot \varepsilon}$$

where *V* is the volume of the sample analyzed (3.53 mL), Δ*A* is the difference in average absorbances (between irradiated and unirradiated ferrioxalate solutions) at 510 nm, *l* is the path length, and ε is the molar absorptivity at 510 nm [4].

$$\text{mol Fe}^{2+} = \frac{V \cdot \Delta A}{l \cdot \varepsilon} = \frac{(0.00353\ L) \cdot (1.521968583)}{(1.00\ cm) \cdot (11100\ {}^{L}/_{mol \cdot cm})} = 4.83 \cdot 10^{-7}\ mol$$

The fraction of light absorbed by the ferrioxalate actinometer was calculated by the following equation:

$$\text{f} = 1 - 10^{-A}$$

where *A* is the absorbance at 468 nm of the ferrioxalate actinometer solution prior to irradiation and addition of phenanthroline (vide infra).

$$\text{f} = 1 - 10^{-A} = 1 - 10^{-0.4890742302} = 0.676$$

The photon flux was calculated using the following equation:

$$\text{photon flux} = \frac{mol\ Fe^{2+}}{\Phi \cdot t \cdot f}$$

where Φ is the quantum yield for the ferrioxalate actinometer at 468 nm [4], *t* is the time and f is the fraction of light absorbed by the ferrioxalate actinometer solution.

$$\text{photon flux} = \frac{mol\ Fe^{2+}}{\Phi \cdot t \cdot f} = \frac{4.83 \cdot 10^{-7}\ mol}{(0.92) \cdot (90\ s) \cdot (0.676)} = 8.64 \cdot 10^{-9}\ {}^{einstein}/_{s}$$

4. Hatchard, C. G.; Parker, C. A. *Proc. R. Soc. London Ser. A* **1956**, *235*, 518−536.

***Determination of fraction of light absorbed at 468 nm for the ferrioxalate solution***

The absorbance at 468 nm of the ferrioxalate actinometer solution prior to irradiation and addition of phenanthroline was measured to be 0.4890742302 (Figure S5).

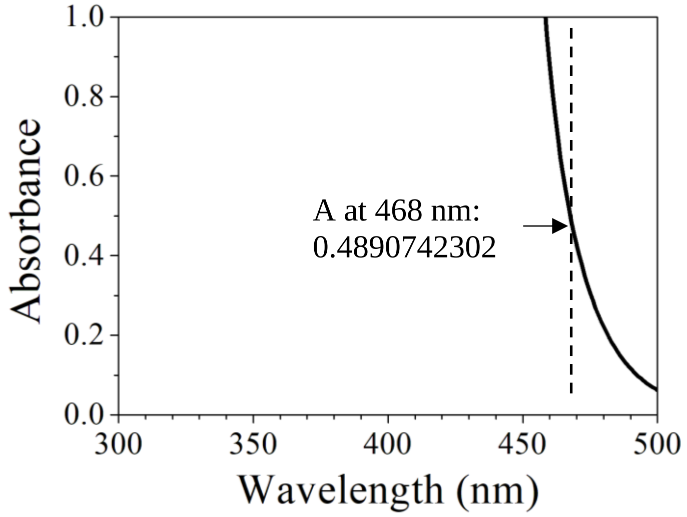


**Figure S5:** UV–vis absorption spectrum of ferrioxalate actinometer solution prior to irradiation and addition of phenanthroline.

***Determination of quantum yield***

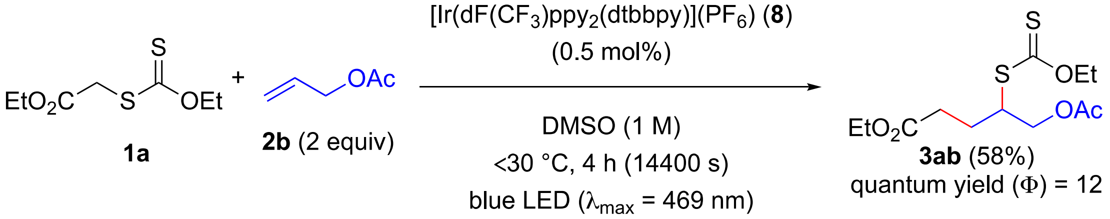


A 4 mL vial was charged with xanthate **1a** (458 mg, 2.20 mmol), alkene **2b** (480 µL, 4.45 mmol), **8** (12.2 mg, 0.0109 mmol), and degassed anhydrous DMSO (2.2 mL). The vial was fitted with a cap and sealed with parafilm. The mixture was stirred and irradiated with blue LED ($\lambda_{max}$ = 469 nm) for 4 hours (14400 s). After irradiation, the vial was removed from the blue LED and the reaction mixture was was purified as detailed below (section 9.1, page S14) to provide the desired product (391 mg, 1.27 mmol) in 58% yield.

The quantum yield (Φ) was calculated using the following equation:

$$\Phi = \frac{mol\,product}{flux \cdot t \cdot f}$$

where $t$ is the reaction time and $f$ is the fraction of light absorbed by **8** that was calculated using the following equation:

$$f = 1 - 10^{-A} = 1 - 10^{-0.811556876} = 0.846$$

where *A* is the absorbance at 468 nm of the **8** solution (5 mM in DMSO) (vide infra).

$$\Phi = \frac{mol\ product}{flux \cdot t \cdot f} = \frac{0.00127\ mol}{(8.64 \cdot 10^{-9}\ ^{einstein}/_{s}) \cdot (14400\ s) \cdot (0.846)} = 12$$

***Absorbance of photocatalyst 8***

The absorbance of **8** in DMSO was measured at the reaction concentration of 5 mM or a dilute concentration of 25 μM (Figure S6). The absorbance at 468 nm for a 5 mM is 0.811556876.

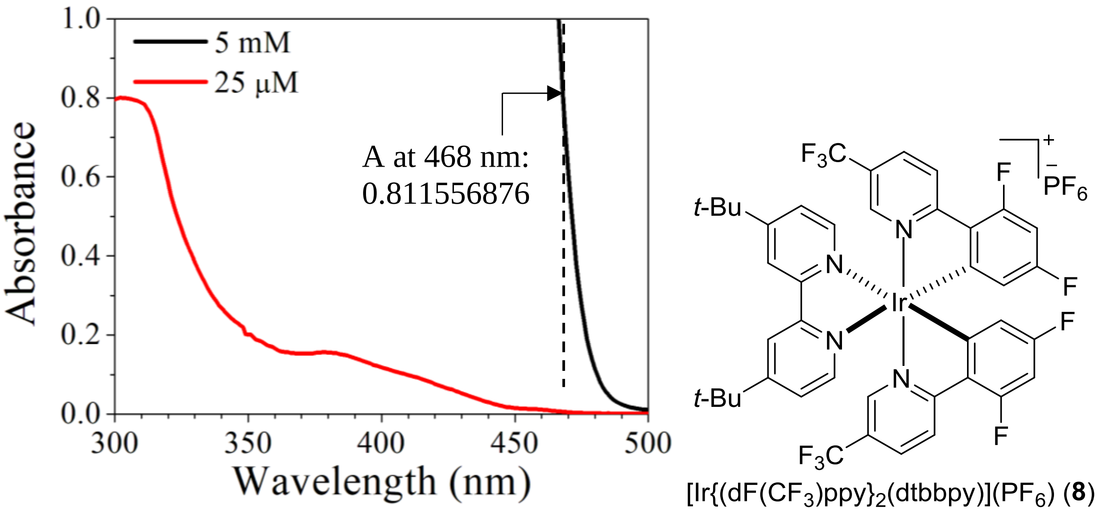


[Ir{(dF(CF$_3$)ppy}$_2$(dtbbpy)](PF$_6$) (**8**)

**Figure S6:** UV–vis absorption spectra of [Ir{dF(CF$_3$)ppy}$_2$(dtbbpy)](PF$_6$) (**8**) (black line: 5 mM in DMSO, red line: 25 μM in DMSO).

## 8. Synthesis of starting materials

Xanthates **1a**,**b** [5], **1c** [6], **1d** [7], **1e** [5], **1f** [8], **1g** [9], **1h** [10], **1i**-**1j** [11], and **1k** [12] were synthesized based on the corresponding literature procedures. Their spectra data are identical to those reported.

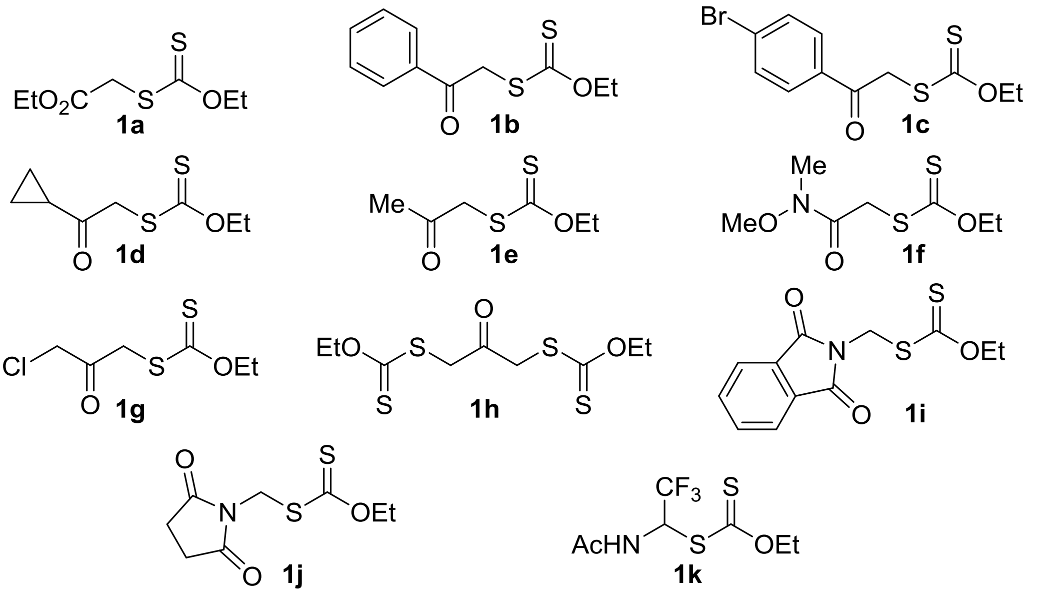


Alkenes **2a** [CAS: 111-66-0], **2b** [CAS: 591-87-7], **2c** [CAS: 109-75-1], **2d** [CAS: 762-72-1], **2e** [CAS: 557-31-3], **2g** [CAS: 72824-04-5], **2h** [CAS: 821-41-0], **2i** [CAS: 2695-47-8], **2k** [CAS: 1120-56-5], **2l** [CAS: 1528-30-9], and **2m** [CAS: 498-66-8] were commercially available and used as received. Alkenes **2f** [13], **2j** [14], **2n** [15], and **2o** [16] were synthesized based on the corresponding literature procedures. Their spectra data are identical to those reported.

5. Kakaei, S.; Chen, N.; Xu, J. X. *Tetrahedron* **2013**, *69*, 302−309.
6. Chalikidi, P. N.; Nevolina, T. A.; Uchuskin, M. G.; Abaev, V. T.; Butin. A. V. *Chem. Heterocycl. Compd.* **2015**, *51*, 621−629.
7. Hayashi, H.; Kaga, A.; Wang, B.; Gagosz, F.; Chiba, S. *Chem. Commun.* **2018**, *54*, 7535−7538.
8. Liautard, V.; Robert, F.; Landais, Y. *Org. Lett.* **2011**, *13*, 2658−2661.
9. de Greef, M.; Zard, S. Z. *Org. Lett.* **2007**, *9*, 1773−1776.
10. Heo, G.; Lee, D.; Kim, C. G.; Do, J. Y. *Spectrochimica Acta Part A: Molecular and Biomolecular Spectroscopy* **2018**, *188*, 285−290.
11. Quiclet-Sire, B.; Zard, S. Z. *Org. Lett.* **2008**, *10*, 3279−3282.
12. Gagosz, F.; Zard, S. Z. *Org. Lett.* **2003**, *5*, 2655−2657.
13. Chit Tsui, G.; Menard, F.;Lautens, M. *Org. Lett.* **2010**, *12*, 2456−2459.
14. Al-Majid, A.; Al-Othman, Z. A.; Islam, M. S. *Helv. Chim. Acta* **2012**, *95*, 268−277.
15. Nečas, D.; Turský, M.; Kotora, M. *J. Am. Chem. Soc.* **2004**, *126*, 10222−10223.
16. Terada, Y.; Arisawa, M.; Nishida, A. *Angew. Chem., Int. Ed.* **2004**, *43*, 4063−4067.

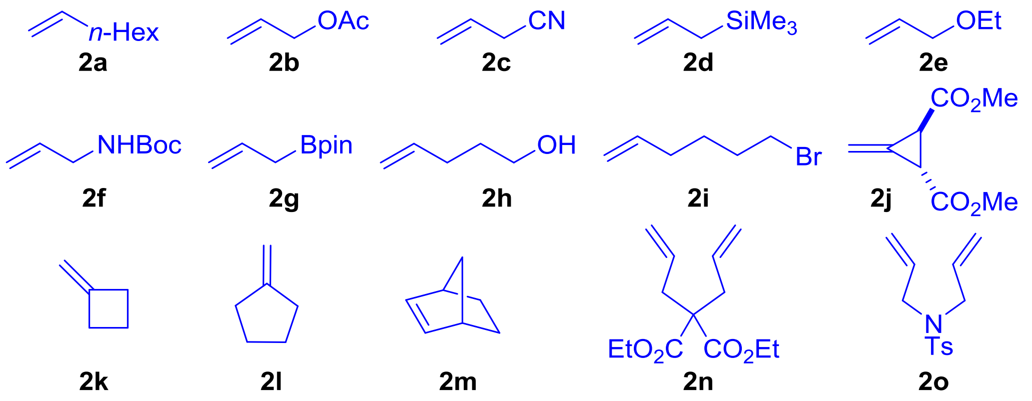


**8.1. Synthesis of (3*R*,5*S*,8*R*,9*S*,10*S*,13*S*,14*S*)-10,13-dimethyl-17-oxohexadecahydro-1*H*-cyclopenta[*a*]phenanthren-3-yl pent-4-enoate (2p)**

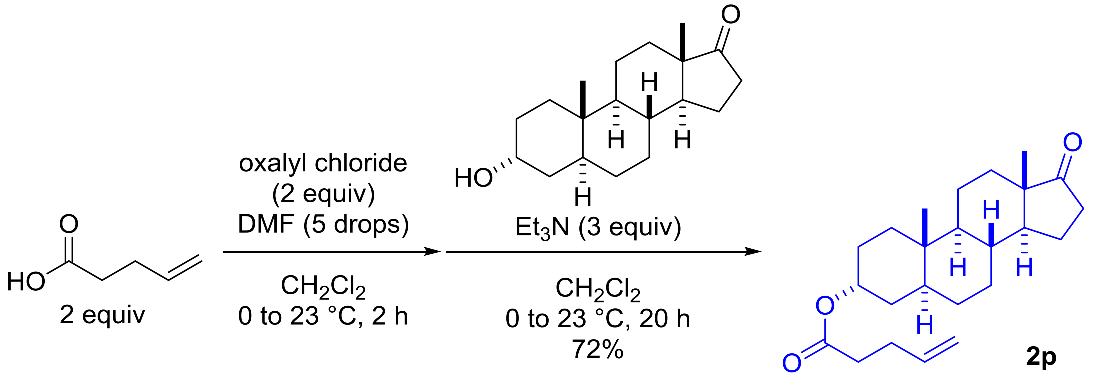


To a solution of 4-pentenoic acid (702 μL, 6.88 mmol) in $CH_2Cl_2$ (20 mL) was added oxalyl chloride (590 μL, 6.90 mmol) and 5 drops of DMF slowly at 0 °C under a $N_2$ atmosphere. After stirring at 23 °C for 2 h, a solution of androsterone (CAS: 53-41-8, (1.00 g, 3.44 mmol) in $CH_2Cl_2$ (20 mL) and $Et_3N$ (1.44 mL, 10.3 mmol) were added slowly at 0 °C under a $N_2$ atmosphere. The reaction mixture was stirred at 23 °C for 20 h and quenched with saturated aqueous $NH_4Cl$. The organic materials were extracted thrice with $CH_2Cl_2$ and the combined organic extracts were washed with water and brine, dried over $MgSO_4$, filtered and concentrated in vacuo. The resulting crude material was purified by flash column chromatography (silica gel: hexane/EtOAc 95:5) to yield alkene **2p** (924 mg, 2.48 mmol) in 72% yield based on androsterone as a white solid.

**mp:** 91-92 °C.

**$^1$H NMR (400 MHz, $CDCl_3$):** δ 5.88-5.79 (1H, m), 5.09-4.99 (3H, m), 2.48-2.36 (5H, m), 2.11-2.02 (1H, m), 1.98-1.91 (1H, m), 1.82-1.80 (2H, m), 1.74-1.45 (9H, m), 1.35-1.17 (6H, m), 1.07-0.97 (1H, m), 0.87 (3H, s), 0.83 (3H, s), 0.81-0.78 (1H, m).

**$^{13}$C NMR (100 MHz, CDCl$_3$):** δ 221.1, 172.4, 136.7, 115.3, 69.7, 54.3, 51.4, 47.7, 40.0, 35.9, 35.7, 34.9, 33.9, 32.83, 32.77, 31.5, 30.7, 29.0, 28.0, 26.0, 21.7, 20.0, 13.7, 11.3.

**ESIHRMS:** Found: m/z 373.2747; Calcd for $C_{24}H_{37}O_3$ $(M+H)^+$ 373.2743.

**8.2. Synthesis of (8*R*,9*S*,10*R*,13*S*,14*S*,17*S*)-10,13-dimethyl-3-oxo-2,3,6,7,8,9,10,11,12,13,14,15,16,17-tetradecahydro-1*H*-cyclopenta[*a*]phenanthren-17-yl pent-4-enoate (2q)**

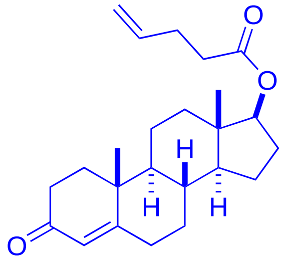


Prepared using testosterone (CAS: 58-22-0) (496 mg, 1.72 mmol) by the procedure described in section 8.1.

Purification: Hexane/EtOAc 95:5-90:10

Yield: 475 mg (1.28 mmol, 75% yield) as a white solid.

**mp:** 131-132 °C.

**$^{1}$H NMR (400 MHz, CDCl$_3$):** δ 5.83 (1H, ddt, *J* = 17.2, 10.4, 6.0 Hz), 5.73 (1H, s), 5.06 (1H, d, *J* = 17.2 Hz), 5.01 (1H, d, *J* = 10.4 Hz), 4.62 (1H, t, *J* = 8.0 Hz), 2.47-2.27 (8H, m), 2.23-2.13 (1H, m), 2.06-2.00 (1H, m), 1.87-1.30 (9H, m), 1.22-1.15 (3H, s + 1H, m), 1.10-1.02 (2H, m), 0.99-0.92 (1H, m), 0.84 (3H, s).

**$^{13}$C NMR (100 MHz, CDCl$_3$):** δ 199.4, 173.0, 170.9, 136.7, 123.9, 115.4, 82.4, 53.7, 50.2, 42.5, 38.6, 36.6, 35.7, 35.4, 33.9, 33.7, 32.7, 31.5, 29.0, 27.5, 23.5, 20.5, 17.4, 12.0.

**ESIHRMS:** Found: m/z 371.2582; Calcd for $C_{24}H_{35}O_3$ $(M+H)^+$ 371.2586.

## 9. Photocatalytic degenerative xanthate transfer to olefins

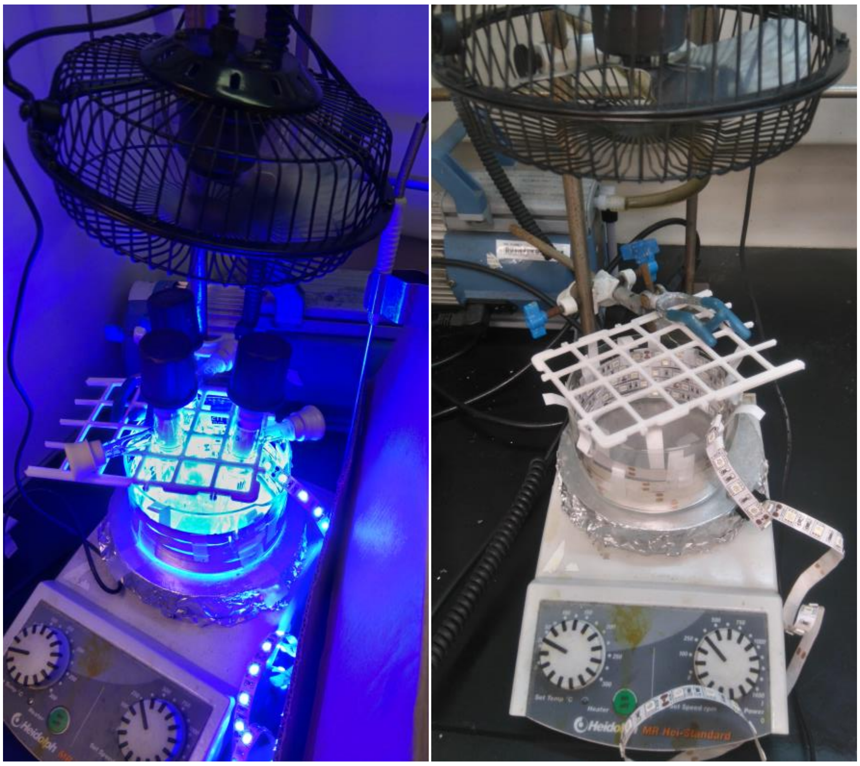

### 9.1. Synthesis of ethyl 4-((ethoxycarbonothioyl)thio)decanoate (3aa) (Table 1, entry 3) (a typical procedure)

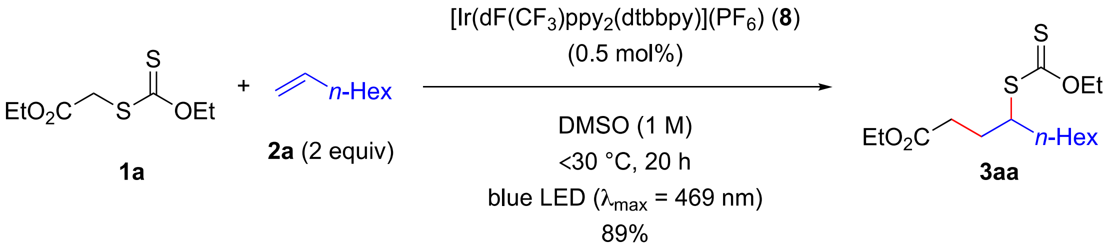


To a 10 mL sealed tube was added ethyl 2-((ethoxycarbonothioyl)thio)acetate (**1a**, 61.3 mg, 0.294 mmol), [Ir{dF($CF_3$)ppy}$_2$(dtbbpy)]($PF_6$) (**8**, 1.6 mg, 0.00143 mmol), and 1-octene (**2a**) (90 μL, 0.573 mmol). Degassed anhydrous DMSO (290 μL) was added and the reaction was sparged with Ar. The tube was sealed and surrounded by blue LEDs for 20 h. The reaction mixture was diluted with EtOAc and water. The aqueous layer was separated and extracted twice with EtOAc. The combined organic extracts were washed with brine, dried over $MgSO_4$, filtered and concentrated in vacuo. The resulting crude material was purified by flash column chromatography (silica gel; hexane/EtOAc 200:1) to give **3aa** (84.3 mg, 0.263 mmol) in 89% yield as a colorless oil.

**$^1$H NMR (400 MHz, $CDCl_3$):** δ 4.64 (2H, q, *J* = 7.2 Hz), 4.13 (2H, q, *J* = 7.2 Hz), 3.80-3.73 (1H, m), 2.51-2.39 (2H, m), 2.14-2.05 (1H, m), 1.96-1.86 (1H, m), 1.69-1.64 (2H, m), 1.47-1.40 (3H, t, *J* = 7.2 Hz + 2H, m), 1.30-1.24 (3H, t, *J* = 7.2 Hz + 6H, m), 0.88 (3H, t, *J* = 6.8 Hz).

**$^{13}$C NMR (100 MHz, $CDCl_3$):** δ 214.5, 173.0, 69.8, 60.4, 50.8, 34.3, 31.6 (overlapped), 29.5, 29.0, 26.7, 22.5, 14.2, 14.0, 13.8.

**ESIHRMS:** Found: m/z 321.1553; Calcd for $C_{15}H_{29}O_3S_2$: $(M+H)^+$ 321.1558.

**9.2. Synthesis of ethyl 5-acetoxy-4-((ethoxycarbonothioyl)thio)pentanoate (3ab)** [17] **(Table 2, entry 1)**

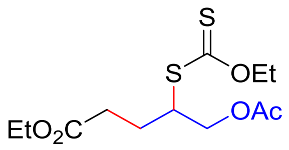


Prepared using xanthate **1a** (61.8 mg, 0.297 mmol) and alkene **2b** (70 μL, 0.649 mmol) with **8** (1.6 mg, 0.00143 mmol) for 18 h.

Purification: Hexane:EtOAc = 10:1

Yield: 80.8 mg (0.262 mmol, 88% yield) as a colorless oil.

**$^1$H NMR (400 MHz, $CDCl_3$):** δ 4.65 (2H, q, *J* = 7.2 Hz), 4.31 (1H, dd, *J* = 11.4, 5.0 Hz), 4.24 (1H, dd, *J* = 11.4, 6.0 Hz), 4.14 (2H, q, *J* = 7.2 Hz), 4.05-3.99 (1H, m), 2.57-2.42 (2H, m), 2.20-2.12 (1H, m), 2.08 (3H, s), 1.98-1.89 (1H, m), 1.43 (3H, t, *J* = 7.2 Hz), 1.26 (3H, t, *J* = 7.2 Hz).

**$^{13}$C NMR (100 MHz, $CDCl_3$):** δ 212.8, 172.5, 170.6, 70.3, 65.5, 60.6, 48.8, 31.5, 26.0, 20.7, 14.2, 13.7.

**9.3. Synthesis of ethyl 5-cyano-4-((ethoxycarbonothioyl)thio)pentanoate (3ac) (Table 2, entry 2)**

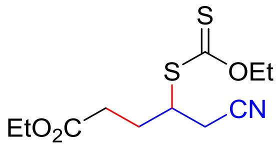


Prepared using xanthate **1a** (63.1 mg, 0.303 mmol) and alkene **2c** (50 μL, 0.622 mmol) with **8** (1.7 mg, 0.00152 mmol) for 45 h.

Purification: Hexane:EtOAc = 7:1

17. Ollivier, C.; Renaud, P. *J. Am. Chem. Soc.* **2000**, *122*, 6496−6497.

Yield: 61.1 mg (0.222 mmol, 73% yield) as a pale yellow oil.

**$^{1}$H NMR (400 MHz, CDCl$_3$):** δ 4.66 (2H, q, *J* = 7.2 Hz), 4.16 (2H, q, *J* = 7.2 Hz), 4.00-3.93 (1H, m), 2.97-2.85 (2H, m), 2.59-2.46 (2H, m), 2.27-2.19 (1H, m), 2.11-2.02 (1H, m), 1.44 (3H, t, *J* = 7.2 Hz), 1.27 (3H, t, *J* = 7.2 Hz).

**$^{13}$C NMR (100 MHz, CDCl$_3$):** δ 212.0, 172.0, 116.8, 70.6, 60.8, 45.9, 31.4, 27.6, 24.1, 14.2, 13.7.

**ESIHRMS:** Found: m/z 276.0723; Calcd for $C_{11}H_{18}NO_3S_2$: $(M+H)^+$ 276.0728.

**9.4. Synthesis of ethyl 4-((ethoxycarbonothioyl)thio)-5-(trimethylsilyl)pentanoate (3ad) (Table 2, entry 3)**

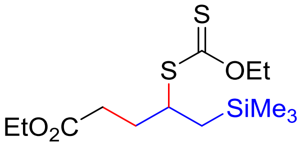


Prepared using xanthate **1a** (65.1 mg, 0.313 mmol) and alkene **2d** (100 μL, 0.629 mmol) with **8** (1.7 mg, 0.00152 mmol) for 46 h.

Purification: Hexane:EtOAc = 140:1

Yield: 85.8 mg (0.266 mmol, 85% yield) as a pale yellow oil.

**$^{1}$H NMR (400 MHz, CDCl$_3$):** δ 4.69-4.57 (2H, m), 4.12 (2H, q, *J* = 7.2 Hz), 3.93-3.86 (1H, m), 2.44 (2H, t, *J* = 7.8 Hz), 2.17-2.08 (1H, m), 1.95-1.86 (1H, m), 1.41 (3H, t, *J* = 7.2 Hz), 1.25 (3H, t, *J* = 7.2 Hz), 1.11 (1H, dd, *J* = 15.2, 7.2 Hz), 0.99 (1H, dd, *J* = 15.2, 8.0 Hz), 0.075 (9H, s).

**$^{13}$C NMR (100 MHz, CDCl$_3$):** δ 214.2, 173.0, 69.6, 60.4, 48.0, 32.1, 31.5, 23.2, 14.2, 13.8, -0.79.

**ESIHRMS:** Found: m/z 323.1174; Calcd for $C_{13}H_{27}O_3SiS_2$: $(M+H)^+$ 323.1171.

**9.5. Synthesis of ethyl 5-ethoxy-4-((ethoxycarbonothioyl)thio)pentanoate (3ae)**[17] **(Table 2, entry 4)**

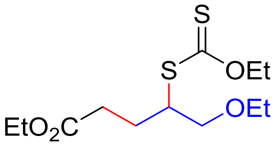


Prepared using xanthate **1a** (63.4 mg, 0.304 mmol) and alkene **2e** (70 μL, 0.618 mmol) with **8** (3.4 mg, 0.00303 mmol) for 41 h.

Purification: Hexane:EtOAc = 80:1

Yield: 63.1 mg (0.214 mmol, 70% yield) as a pale yellow oil.

**$^{1}$H NMR (400 MHz, $CDCl_3$):** δ 4.64 (2H, q, *J* = 7.2 Hz), 4.13 (2H, q, *J* = 7.2 Hz), 3.99-3.93 (1H, m), 3.68 (1H, dd, *J* = 10.0, 4.4 Hz), 3.59-3.46 (2H, q, *J* = 7.2 Hz + 1H, m), 2.56-2.41 (2H, m), 2.27-2.20 (1H, m), 1.99-1.89 (1H, m), 1.42 (3H, t, *J* = 7.2 Hz), 1.26 (3H, t, *J* = 7.2 Hz), 1.20 (3H, t, *J* = 7.2 Hz).

**$^{13}$C NMR (100 MHz, $CDCl_3$):** δ 214.0, 172.9, 72.0, 70.0, 66.6, 60.4, 49.9, 31.7, 26.4, 15.0, 14.2, 13.7.

### 9.6. Synthesis of ethyl 5-((*tert*-butoxycarbonyl)amino)-4-((ethoxycarbonothioyl)thio)pentanoate (3af) (Table 2, entry 5)

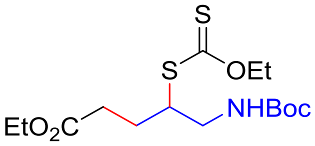


Prepared using xanthate **1a** (103 mg, 0.495 mmol) and alkene **2f** (159 mg, 1.01 mmol) with **8** (5.6 mg, 0.00499 mmol) for 61 h.

Purification: Hexane:EtOAc = 92:8

Yield: 146 mg (0.398 mmol, 80% yield) as a colorless oil.

**$^{1}$H NMR (400 MHz, $CDCl_3$):** δ 4.87 (1H, s br), 4.65 (2H, q, *J* = 7.2 Hz), 4.13 (2H, q, *J* = 7.2 Hz), 3.90-3.84 (1H, m), 3.49 (1H, dd, *J* = 13.6, 6.0 Hz), 3.35 (1H, dd, *J* = 13.6, 6.4 Hz), 2.57-2.43 (2H, m), 2.16-2.07 (1H, m), 1.94-1.84 (1H, m), 1.44-1.41 (9H, s + 3H, t, *J* = 7.2 Hz), 1.25 (3H, t, *J* = 7.2 Hz).

**$^{13}$C NMR (100 MHz, $CDCl_3$):** δ 213.2, 172.7, 155.8, 79.5, 70.2, 60.5, 51.2, 43.7, 31.5, 28.3, 26.5, 14.2, 13.7.

**ESIHRMS:** Found: m/z 366.1408; Calcd for $C_{15}H_{28}NO_5S_2$: $(M+H)^+$ 366.1409.

**9.7. Synthesis of ethyl 4-((ethoxycarbonothioyl)thio)-5-(4,4,5,5-tetramethyl-1,3,2-dioxaborolan-2-yl)pentanoate (3ag) (Table 2, entry 6)**

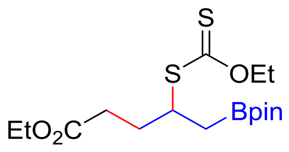


Prepared using xanthate **1a** (63.8 mg, 0.306 mmol) and alkene **2g** (230 μL, 1.23 mmol) with **8** (1.8 mg, 0.00160 mmol) for 30 h.

Purification: Hexane:EtOAc = 20:1

Yield: 82.8 mg (0.220 mmol, 72% yield) as a pale yellow oil.

**$^1$H NMR (400 MHz, $CDCl_3$):** δ 4.64 (2H, q, *J* = 7.2 Hz), 4.12 (2H, q, *J* = 7.2 Hz), 4.00-3.93 (1H, m), 2.46-2.42 (2H, m), 2.15-1.98 (2H, m), 1.42 (3H, t, *J* = 7.2 Hz), 1.31 (2H, t, *J* = 6.4 Hz), 1.27-1.24 (3H, t, *J* = 7.2 Hz + 12H, m).

**$^{13}$C NMR (100 MHz, $CDCl_3$):** δ 214.1, 173.0, 83.5, 69.6, 60.4, 47.0, 31.8, 31.3, 24.8, 24.7, 14.2, 13.7.

**ESIHRMS:** Found: m/z 377.1629; Calcd for $C_{16}H_{30}O_5S_2B$: $(M+H)^+$ 377.1628.

**9.8. Synthesis of ethyl 4-((ethoxycarbonothioyl)thio)-7-hydroxyheptanoate (3ah) (Table 2, entry 7)**

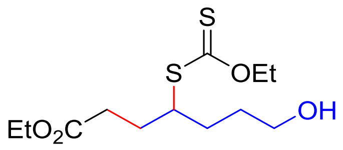


Prepared using xanthate **1a** (63.0 mg, 0.302 mmol) and alkene **2h** (60 μL, 0.581 mmol) with **8** (1.7 mg, 0.00152 mmol) for 37 h.

Purification: Hexane:EtOAc = 2:1

Yield: 75.1 mg (0.255 mmol, 84% yield) as a pale yellow oil.

**$^1$H NMR (400 MHz, $CDCl_3$):** δ 4.64 (2H, q, *J* = 7.2 Hz), 4.13 (2H, q, *J* = 7.2 Hz), 3.84-3.77 (1H, m), 3.67 (2H, t, *J* = 6.0 Hz), 2.53-2.41 (2H, m), 2.14-2.08 (1H, m), 1.97-1.65 (5H, m), 1.57 (1H, s br), 1.42 (3H, t, *J* = 7.2 Hz), 1.26 (3H, t, *J* = 7.2 Hz).

**$^{13}$C NMR (100 MHz, $CDCl_3$):** δ 214.3, 173.0, 70.0, 62.3, 60.5, 50.5, 31.6, 30.7, 29.7, 29.4, 14.2, 13.7.

**ESIHRMS:** Found: m/z 295.1042; Calcd for $C_{12}H_{23}O_4S_2$: $(M+H)^+$ 295.1038.

## 9.9. Synthesis of ethyl 8-bromo-4-((ethoxycarbonothioyl)thio)octanoate (3ai) (Table 2, entry 8)

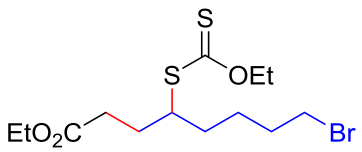


Prepared using xanthate **1a** (64.0 mg, 0.307 mmol) and alkene **2i** (85 μL, 0.636 mmol) with **8** (1.7 mg, 0.00152 mmol) for 48 h.

Purification: Hexane:$Et_2O$ = 60:1

Yield: 83.0 mg (0.224 mmol, 73% yield) as a colorless oil.

**$^1$H NMR (400 MHz, $CDCl_3$):** δ 4.64 (2H, q, *J* = 7.2 Hz), 4.13 (2H, q, *J* = 7.2 Hz), 3.81-3.75 (1H, m), 3.41 (2H, t, *J* = 6.8 Hz), 2.52-2.40 (2H, m), 2.13-2.07 (1H, m), 1.95-1.87 (3H, m), 1.74-1.56 (4H, m), 1.43 (3H, t, *J* = 7.2 Hz), 1.26 (3H, t, *J* = 7.2 Hz).

**$^{13}$C NMR (100 MHz, $CDCl_3$):** δ 214.2, 172.9, 70.0, 60.5, 50.5, 33.6, 33.3, 32.4, 31.6, 29.3, 25.4, 14.2, 13.8.

**ESIHRMS:** Found: m/z 371.0349; Calcd for $C_{13}H_{24}O_3S_2Br$: $(M+H)^+$ 371.0350.

## 9.10. Synthesis of dimethyl (1*S**,2*S**)-3-(3-ethoxy-3-oxopropyl)-3-((ethoxy-carbonothioyl)thio)cyclopropane-1,2-dicarboxylate (3aj) (Table 2, entry 9)

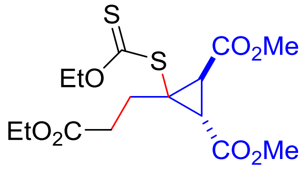


Prepared using xanthate **1a** (104 mg, 0.501 mmol) and alkene **2j** (170 mg, 0.999 mmol) with **8** (2.8 mg, 0.00250 mmol) for 48 h.

Purification: Hexane:EtOAc = 88:12 – 85:15

Yield: 122 mg (0.322 mmol, 64% yield) as an yellow oil.

**$^1$H NMR (400 MHz, $CDCl_3$):** δ 4.70-4.53 (2H, m), 4.12 (2H, q, *J* = 7.2 Hz), 3.77 (3H, s), 3.70 (3H, s), 2.74 (1H, d, *J* = 6.8 Hz), 2.67 (1H, d, *J* = 6.8 Hz), 2.61-2.38 (3H, m), 2.24-2.17 (1H, m), 1.44 (3H, t, *J* = 7.2 Hz), 1.25 (3H, t, *J* = 7.2 Hz).

**$^{13}$C NMR (100 MHz, $CDCl_3$):** δ 210.1, 172.3, 168.8, 167.9, 70.1, 60.6, 52.6, 52.5, 41.6, 34.2, 34.0, 32.0, 28.1, 14.1, 13.6.

**ESIHRMS:** Found: m/z 379.0889; Calcd for $C_{15}H_{23}O_7S_2$: $(M+H)^+$ 379.0885.

**9.11. Synthesis of ethyl 3-(1-((ethoxycarbonothioyl)thio)cyclobutyl)propanoate (3ak) (Table 2, entry 10)**

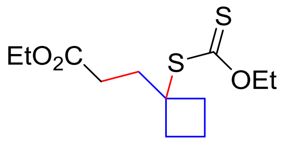


Prepared using xanthate **1a** (62.0 mg, 0.298 mmol) and alkene **2k** (60 μL, 0.648 mmol) with **8** (1.6 mg, 0.00143 mmol) for 26 h.

Purification: Hexane:EtOAc = 120:1

Yield: 74.1 mg (0.268 mmol, 90% yield) as a pale yellow oil.

**$^{1}$H NMR (400 MHz, $CDCl_3$):** δ 4.63 (2H, q, *J* = 7.2 Hz), 4.13 (2H, q, *J* = 7.2 Hz), 2.40-2.28 (6H, m), 2.23-2.16 (3H, m), 2.04-1.93 (1H, m), 1.43 (3H, t, *J* = 7.2 Hz), 1.26 (3H, t, *J* = 7.2 Hz).

**$^{13}$C NMR (100 MHz, $CDCl_3$):** δ 213.0, 173.3, 69.2, 60.5, 55.9, 34.1, 33.9, 30.4, 16.6, 14.2, 13.7.

**ESIHRMS:** Found: m/z 277.0929; Calcd for $C_{12}H_{21}O_3S_2$: $(M+H)^+$ 277.0932.

**9.12. Synthesis of ethyl 3-(1-((ethoxycarbonothioyl)thio)cyclobutyl)propanoate (3al) and ethyl 3-(cyclopent-1-en-1-yl)propanoate (3al’) (Table 2, entry 11)**

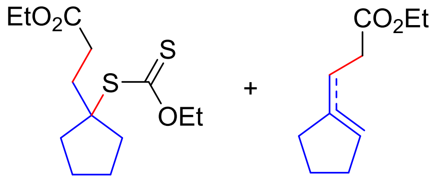


Prepared using xanthate **1a** (62.5 mg, 0.300 mmol) and alkene **2l** (130 μL, 1.24 mmol) with **8** (1.8 mg, 0.00160 mmol) for 20 h.

Purification: Hexane:$Et_2O$ = 30:1

Yield of **3al**: 52.1 mg (0.179 mmol, 60% yield) as an yellow oil.

**$^{1}$H NMR (400 MHz, $CDCl_3$):** δ 4.67 (2H, q, *J* = 7.2 Hz), 4.13 (2H, q, *J* = 7.2 Hz), 2.48-2.44 (2H, m), 2.31-2.27 (2H, m), 2.06-1.98 (2H, m), 1.80-1.70 (6H, m), 1.46 (3H, t, *J* = 7.2 Hz), 1.26 (3H, t, *J* = 7.2 Hz).

**$^{13}$C NMR (100 MHz, $CDCl_3$):** δ 213.9, 173.4, 69.4, 64.3, 60.5, 38.8, 34.6, 31.5, 24.4, 14.2, 13.7.

**ESIHRMS:** Found: m/z 291.1093; Calcd for $C_{13}H_{23}O_3S_2$: $(M+H)^+$ 291.1089.

Yield of **3al'** [18] (18 % yield, *endo*/*exo* = 80:20) was determined by crude $^{1}$H-NMR using 1,1,2,2-tetrachloroethane (49.3 mg, 0.294 mmol) as an internal standard. The formation of **3al'** presumably occurs through the single-electron-oxidation of resulting tertiary alkyl radical species by photo-excited **8***, generating carbocation intermediate followed by deprotonation.

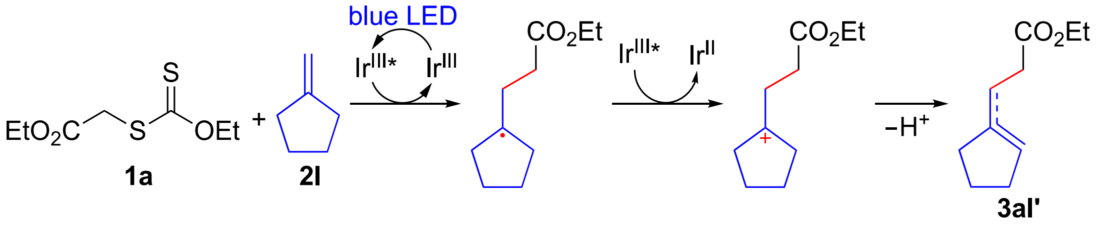


## 9.13. Synthesis of ethyl 2-((1*R**,4*S**)-3-((ethoxycarbonothioyl)thio)bicyclo[2.2.1]heptan-2-yl)acetate (3am) (Table 2, entry 12)

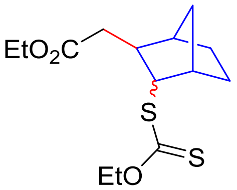


Prepared using xanthate **1a** (62.4 mg, 0.300 mmol) and alkene **2m** (56.9 mg, 0.604 mmol) with **8** (1.7 mg, 0.00152 mmol) for 10 h.

Purification: Hexane:EtOAc = 98:2 to 97:3

Yield: 66.6 mg (0.220 mmol, 73% yield) as a pale-yellow oil (dr = 78:22, determined by $^{1}$H NMR analysis as an inseparable mixture of diastereoisomers).

**$^{1}$H NMR (400 MHz, $CDCl_3$):** *for the mixture of diastereoisomers* δ 4.69-4.57 (2H + 2H×0.28, m), 4.14-4.09 (2H + 2H×0.28, m), 3.85 (1H×0.28, dd, *J* = 8.4, 2.0 Hz), 3.56-3.53 (1H, m), 2.63 (1H, m), 2.53-2.49 (1H×0.28, m), 2.45 (1H, dd, *J* = 14.8, 5.6 Hz), 2.40-2.39 (1H×0.28, m), 2.32 (1H, dd, *J* = 14.8, 9.6 Hz), 2.22 (1H×0.28, dd, *J* = 15.6, 10.8 Hz), 2.10-2.09 (1H + 1H×0.28, m), 1.76-1.18 (13H + 13H×0.28, m).

**$^{13}$C NMR (100 MHz, $CDCl_3$):** *for major-**3am*** δ 215.1, 172.1, 69.7, 60.4, 56.5, 44.8, 41.4, 40.9, 39.8, 36.0, 29.5, 23.2, 14.2, 13.8.

**ESIHRMS:** *for the mixture of diastereoisomers* Found: m/z 303.1091; Calcd for $C_{14}H_{23}O_3S_2$: $(M+H)^+$ 303.1089.

18. Meyer, D.; Vin, E.; Wyler, B.; Lapointe, G.; Renaud, P. *Synlett* **2016**, *27*, 745−748.

**9.14. Synthesis of diethyl 3-(3-ethoxy-3-oxopropyl)-4-(((ethoxycarbonothioyl)thio)methyl)cyclopentane-1,1-dicarboxylate (3an)** [19] **(Table 2, entry 13)**

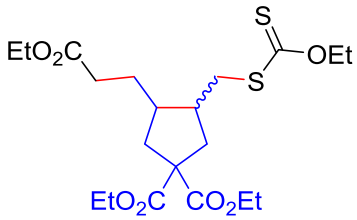


Prepared using xanthate **1a** (102 mg, 0.488 mmol) and alkene **2n** (236 mg, 0.983 mmol) with **8** (2.6 mg, 0.00232 mmol) for 24 h.

Purification: Hexane:EtOAc = 15:1

Yield: 149 mg (0.331 mmol, 68% yield) as a pale-yellow oil (dr = 88:12, determined by $^1$H NMR analysis as an inseparable mixture of diastereoisomers).

**$^1$H NMR (400 MHz, $CDCl_3$):** *for the mixture of diastereoisomers* δ 4.64 (2H + 2H×0.14, q, $J$ = 7.2 Hz), 4.20-4.11 (6H + 6H×0.14, m), 3.48 (1H×0.14, dd, $J$ = 13.6, 4.0 Hz), 3.31 (1H, dd, $J$ = 13.6, 5.6 Hz), 2.97-2.94 (1H×0.14, m), 2.89 (1H, dd, $J$ = 13.6, 9.6 Hz), 2.55-2.30 (5H + 5H×0.14, m), 2.25-2.19 (1H + 1H×0.14, m), 2.13-2.00 (2H + 2H×0.14, m), 1.86-1.77 (1H + 1H×0.14, m), 1.63-1.52 (1H + 1H×0.14, m), 1.42 (3H + 3H×0.14, t, $J$ = 7.2 Hz), 1.28-1.22 (9H + 9H×0.14, m).

**$^{13}$C NMR (100 MHz, $CDCl_3$):** *for major-**3an*** δ 214.6, 173.2, 172.5, 172.3, 69.9, 61.6, 61.5, 60.4, 58.5, 41.9, 40.7, 38.5, 38.1, 36.1, 32.9, 24.5, 14.2, 14.0 (overlapped), 13.8.

**9.15. Synthesis of ethyl 3-(4-(((ethoxycarbonothioyl)thio)methyl)-1-tosylpyrrolidin-3-yl)propanoate (3ao) (Table 2, entry 14)**

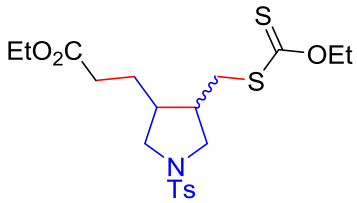


Prepared using xanthate **1a** (105 mg, 0.502 mmol) and alkene **2o** (252 mg, 1.00 mmol) with **8** (2.8 mg, 0.00250 mmol) for 13 h.

Purification: Hexane:EtOAc = 4:1

Yield: 170 mg (0.370 mmol, 74% yield) as a colorless oil (dr = 71:29, determined by $^1$H NMR analysis as an inseparable mixture of diastereoisomers).

---

19. Beniazza, R.; Liautard, V.; Poittevin, C.; Ovadia, B.; Mohammed, S.; Robert, F.; Landais, Y. *Chem. Eur. J.* **2017**, *23*, 2439−2447.

**$^{1}$H NMR (400 MHz, $CDCl_3$):** *for the mixture of diastereoisomers* δ 7.72-7.68 (2H, d, *J* = 8.0 Hz + 2H×0.40, m), 7.33 (2H + 2H×0.40, d, *J* = 8.0 Hz), 4.66-4.60 (2H + 2H×0.40, m), 4.12 (2H + 2H×0.40, q, *J* = 7.2 Hz), 3.51 (1H×0.40, dd, *J* = 9.6, 7.2 Hz), 3.43 (1H, dd, *J* = 10.0, 7.6 Hz), 3.37-3.27 (2H + 1H×0.40, m), 3.18 (1H, dd, *J* = 13.2, 4.0 Hz), 3.05-3.02 (1H×0.40, m), 2.98 (1H, dd, *J* = 10.0, 8.8 Hz), 2.87-2.81 (2H×0.40, m), 2.48-1.98 (8H + 8H×0.40, m), 1.91-1.81 (2H×0.40, m), 1.79-1.70 (1H, m), 1.55-1.50 (1H, m), 1.41 (3H + 3H×0.40, t, *J* = 7.2 Hz), 1.25 (3H + 3H×0.40, t, *J* = 7.2 Hz).

**$^{13}$C NMR (100 MHz, $CDCl_3$):** *for major-**3ao*** δ 213.8, 172.6, 143.6, 133.6, 129.8, 127.3, 70.2, 60.5, 51.8, 50.9, 41.0, 39.9, 33.8, 32.4, 22.8, 21.5, 14.2, 13.7.

**ESIHRMS:** *for the mixture of diastereoisomers* Found: m/z 460.1291; Calcd for $C_{20}H_{30}NO_5S_3$: $(M+H)^+$ 460.1286.

### 9.16. Synthesis of 1-((3*R*,5*S*,8*R*,9*S*,10*S*,13*S*,14*S*)-10,13-dimethyl-17-oxohexadecahydro-1*H*-cyclopenta[a]phenanthren-3-yl) 7-ethyl 4-((ethoxycarbonothioyl)thio)heptanedioate (3ap) (Table 2, entry 15)

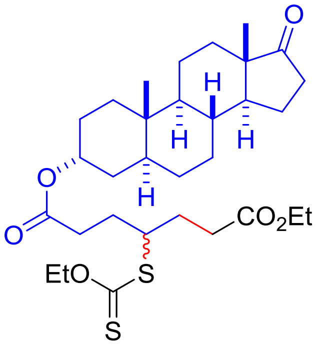


Prepared using xanthate **1a** (61.4 mg, 0.295 mmol) and alkene **2p** (220 mg, 0.591 mmol) with **8** (1.7 mg, 0.00152 mmol) in DMSO/DCE = 1:1 (0.5 M) for 26 h.

Purification: Hexane:EtOAc = 95:5-90:10

Yield: 161 mg (0.277 mmol, 94% yield) as a colorless oil (dr = 50:50, determined by $^{13}$C NMR analysis as an inseparable mixture of diastereoisomers).

**$^{1}$H NMR (400 MHz, $C_6D_6$):** *for the mixture of diastereoisomers* δ 5.13 (1H, m), 4.28-4.23 (2H, m), 3.95-3.88 (3H, m), 2.44-2.39 (2H, m), 2.33-2.29 (2H, m), 2.16-2.10 (1H, m), 2.07-1.94 (2H, m), 1.87-1.72 (5H, m), 1.57-1.37 (6H, m), 1.32-1.17 (7H, m), 1.08-0.73 (11H, m), 0.61 (3H, s), 0.56 (3H, s).

**$^{13}$C NMR (100 MHz, $C_6D_6$):** *for the mixture of diastereoisomers* δ 218.1, 214.4, 214.3, 172.2, 171.70, 171.66, 70.13, 70.12, 70.09, 60.3, 54.52, 54.50, 51.4, 51.13,

51.09, 47.6, 40.5, 40.4, 36.1, 35.7, 35.1, 33.27, 33.25, 33.23, 32.16, 32.1, 31.70, 31.69, 30.99, 30.97, 30.13, 30.09, 30.0, 29.9, 28.48, 28.47, 26.40, 26.36, 21.8, 20.4, 14.3, 13.8, 13.5, 11.3.

**ESIHRMS:** *for the mixture of diastereoisomers* Found: m/z 603.2791; Calcd for $C_{31}H_{48}O_6S_2Na$ $(M+Na)^+$ 603.2790.

**9.17. Synthesis of 1-((8*R*,9*S*,10*R*,13*S*,14*S*,17*S*)-10,13-dimethyl-3-oxo-2,3,6,7,8,9,10,11,12,13,14,15,16,17-tetradecahydro-1*H*-cyclopenta[*a*]phenanthren-17-yl) 7-ethyl 4-((ethoxycarbonothioyl)thio)heptanedioate (3aq) (Table 2, entry 16)**

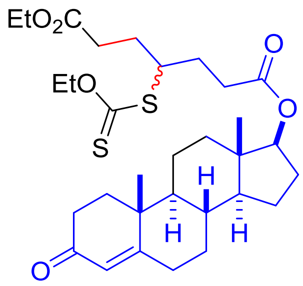


Prepared using xanthate **1a** (66.6 mg, 0.320 mmol) and alkene **2q** (237 mg, 0.639 mmol) with **8** (1.8 mg, 0.00160 mmol) in DMSO/DCE = 3:5 (0.4 M) for 50 h.

Purification: Hexane:EtOAc = 95:5-80:20

Yield: 149 mg (0.257 mmol, 80% yield) as a colorless oil (dr = 50:50, determined by $^{13}C$ NMR analysis as an inseparable mixture of diastereoisomers).

**$^1H$ NMR (400 MHz, $CDCl_3$):** *for the mixture of diastereoisomers* δ 5.73 (1H, s), 4.67-4.59 (3H, m), 4.13 (2H, q, *J* = 7.2 Hz), 3.85-3.78 (1H, m), 2.50-2.35 (7H, m), 2.31-1.47 (15H, m), 1.42 (3H, t, *J* = 7.2 Hz), 1.39-1.33 (2H, m), 1.26 (3H, t, *J* = 7.2 Hz), 1.19 (3H, s), 1.10-1.01 (2H, m), 0.98-0.91 (1H, m), 0.83 (3H, s).

**$^{13}C$ NMR (100 MHz, $CDCl_3$):** *for the mixture of diastereoisomers* δ 213.6, 199.2, 172.7, 172.6, 170.7, 123.8, 82.5, 77.2, 70.0, 60.4, 53.6, 50.3, 50.1, 42.4, 38.5, 36.6, 35.6, 35.3, 33.8, 32.6, 31.62, 31.58, 31.44, 31.39, 29.61, 29.59, 29.5, 27.4, 23.4, 20.4, 17.3, 14.1, 13.7, 12.0.

**ESIHRMS:** *for the mixture of diastereoisomers* Found: m/z 601.2631; Calcd for $C_{31}H_{46}O_6S_2Na$ $(M+Na)^+$ 601.2634.

**9.18. Synthesis of 2-((ethoxycarbonothioyl)thio)-5-oxo-5-phenylpentyl acetate (3bb) (Table 3, entry 1)**

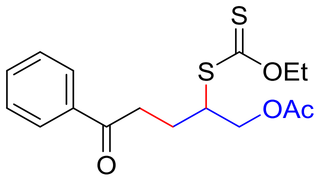


Prepared using xanthate **1b** (72.5 mg, 0.302 mmol) and alkene **2b** (70 µL, 0.649 mmol) with **8** (1.6 mg, 0.00143 mmol) for 44 h.

Purification: Hexane:EtOAc = 8:1

Yield: 80.0 mg (0.235 mmol, 78% yield) as a colorless oil.

**$^1$H NMR (500 MHz, $CDCl_3$):** δ 7.96 (2H, d, $J$ = 7.5 Hz), 7.57 (1H, t, $J$ = 7.5 Hz), 7.47 (2H, dd, $J$ = 7.5, 7.5 Hz), 4.68-4.58 (2H, m), 4.37 (1H, dd, $J$ = 11.5, 5.0 Hz), 4.29 (1H, dd, $J$ = 11.5, 6.5 Hz), 4.11-4.07 (1H, m), 3.24-3.13 (2H, m), 2.36-2.29 (1H, m), 2.08-2.00 (3H, s + 1H, m), 1.41 (3H, t, $J$ = 7.0 Hz).

**$^{13}$C NMR (125 MHz, $CDCl_3$):** δ 212.8, 198.7, 170.7, 136.6, 133.2, 128.6, 128.0, 70.3, 65.7, 49.0, 35.6, 25.0, 20.8, 13.7.

**ESIHRMS:** Found: m/z 341.0884; Calcd for $C_{16}H_{21}O_4S_2$: $(M+H)^+$ 341.0881.

**9.19. Synthesis of 5-(4-bromophenyl)-2-((ethoxycarbonothioyl)thio)-5-oxopentyl acetate (3cb) (Table 3, entry 2)**

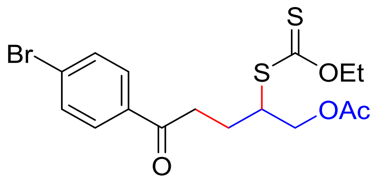


Prepared using xanthate **3c** (97.5 mg, 0.305 mmol) and alkene **1b** (65.9 µL, 0.611 mmol) with **8** (1.7 mg, 0.00152 mmol) for 47 h.

Purification: Hexane:EtOAc = 95:5-93:7

Yield: 87.8 mg (0.209 mmol, 69% yield) as a colorless oil.

**$^1$H NMR (400 MHz, $CDCl_3$):** δ 7.82 (2H, d, $J$ = 8.4 Hz), 7.61 (2H, d, $J$ = 8.4 Hz), 4.69-4.57 (2H, m), 4.36 (1H, dd, $J$ = 11.2, 4.8 Hz), 4.28 (1H, dd, $J$ = 11.2, 6.0 Hz), 4.11-4.04 (1H, m), 3.21-3.07 (2H, m), 2.36-2.27 (1H, m), 2.08 (3H, s), 2.06-1.98 (1H, m), 1.41 (3H, t, $J$ = 7.2 Hz).

**$^{13}$C NMR (100 MHz, $CDCl_3$):** δ 212.8, 197.7, 170.6, 135.4, 132.0, 129.5, 128.4, 70.4, 65.7, 49.0, 35.6, 25.0, 20.8, 13.7.

**ESIHRMS:** Found: m/z 440.9805; Calcd for $C_{16}H_{19}O_4S_2BrNa$ $(M+Na)^+$ 440.9806.

**9.20. Synthesis of 5-cyclopropyl-2-((ethoxycarbonothioyl)thio)-5-oxopentyl acetate (3db) (Table 3, entry 3)**

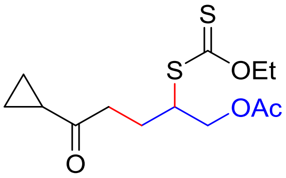


Prepared using xanthate **1d** (62.5 mg, 0.306 mmol) and alkene **2b** (70 μL, 0.649 mmol) with **8** (1.8 mg, 0.00160 mmol) for 51 h.

Purification: Hexane:EtOAc = 10:1

Yield: 55.0 mg (0.181 mmol, 59% yield) as a colorless oil.

**$^1$H NMR (500 MHz, CDCl$_3$):** δ 4.65 (2H, q, *J* = 7.0 Hz), 4.30 (1H, dd, *J* = 11.5, 5.0 Hz), 4.24 (1H, dd, *J* = 11.5, 6.0 Hz), 4.01-3.96 (1H, m), 2.83-2.71 (2H, m), 2.20-2.12 (1H, m), 2.08 (3H, s), 1.94-1.84 (2H, m), 1.43 (3H, t, *J* = 7.0 Hz), 1.03 (2H, dt, *J* = 7.5, 4.0 Hz), 0.88 (2H, dt, *J* = 7.5, 4.0 Hz).

**$^{13}$C NMR (125 MHz, CDCl$_3$):** δ 213.0, 209.3, 170.6, 70.3, 65.6, 48.9, 40.2, 24.6, 20.8, 20.6, 13.7, 10.9.

**ESIHRMS:** Found: m/z 305.0880; Calcd for $C_{13}H_{21}O_4S_2$: $(M+H)^+$ 305.0881.

**9.21. Synthesis of 2-((ethoxycarbonothioyl)thio)-5-oxohexyl acetate (3eb) (Table 3, entry 4)**

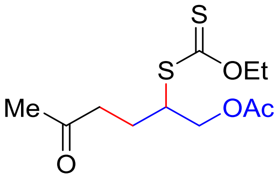


Prepared using xanthate **1e** (53.4 mg, 0.300 mmol) and alkene **2b** (70 μL, 0.649 mmol) with **8** (3.5 mg, 0.00312 mmol) for 71 h.

Purification: Hexane:EtOAc = 8:1

Yield: 68.3 mg (0.245 mmol, 82% yield) as a pale yellow oil.

**$^1$H NMR (500 MHz, CDCl$_3$):** δ 4.65 (2H, q, *J* = 7.0 Hz), 4.30 (1H, dd, *J* = 11.5, 5.0 Hz), 4.23 (1H, dd, *J* = 11.5, 6.0 Hz), 4.00-3.94 (1H, m), 2.70-2.58 (2H, m), 2.16-2.06 (3H, s), 2.15-2.09 (1H, m), 2.08 (3H, s), 1.89-1.81 (1H, m), 1.43 (3H, t, *J* = 7.0 Hz).

**$^{13}$C NMR (125 MHz, CDCl$_3$):** δ 212.9, 207.2, 170.6, 70.3, 65.6, 48.9, 40.5, 30.0,

24.4, 20.7, 13.7.

**ESIHRMS:** Found: m/z 279.0729; Calcd for $C_{11}H_{19}O_4S_2$: $(M+H)^+$ 279.0725.

**9.22. Synthesis of 2-((ethoxycarbonothioyl)thio)-5-(methoxy(methyl)amino)-5-oxo-pentyl acetate (3fb) (Table 3, entry 5)**

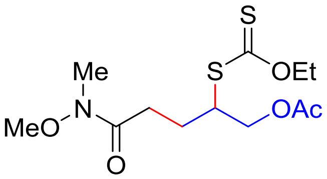


Prepared using xanthate **1f** (66.0 mg, 0.296 mmol) and alkene **2b** (70 μL, 0.649 mmol) with **8** (1.7 mg, 0.00152 mmol) for 27 h.

Purification: Hexane:EtOAc = 7:3

Yield: 77.6 mg (0.240 mmol, 81% yield) as a colorless oil.

**$^1$H NMR (400 MHz, CDCl$_3$):** δ 4.65 (2H, q, *J* = 7.2 Hz), 4.33 (1H, dd, *J* = 11.6, 4.8 Hz), 4.26 (1H, dd, *J* = 11.6, 6.0 Hz), 4.06-3.99 (1H, m), 3.68 (3H, s), 3.18 (3H, s), 2.68-2.55 (2H, m), 2.24-2.15 (1H, m), 2.08 (3H, s), 1.98-1.88 (1H, m), 1.43 (3H, t, *J* = 7.2 Hz).

**$^{13}$C NMR (100 MHz, CDCl$_3$):** δ 213.0, 170.7 (overlapped), 70.2, 65.6, 61.2, 49.0, 32.2, 29.1, 25.5, 20.8, 13.7.

**ESIHRMS:** Found: m/z 324.0935; Calcd for $C_{12}H_{22}NO_5S_2$: $(M+H)^+$ 324.0939.

**9.23. Synthesis of 6-chloro-2-((ethoxycarbonothioyl)thio)-5-oxohexyl acetate (3gb) (Table 3, entry 6)**

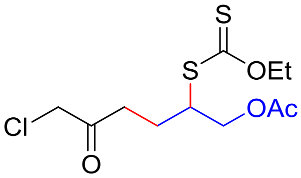


Prepared using xanthate **1g** (63.9 mg, 0.300 mmol) and alkene **2b** (70 μL, 0.649 mmol) with **8** (1.7 mg, 0.00152 mmol) for 17 h.

Purification: Hexane:EtOAc = 6:1

Yield: 70.7 mg (0.226 mmol, 75% yield) as a pale yellow oil.

**$^1$H NMR (400 MHz, CDCl$_3$):** δ 4.65 (2H, q, *J* = 7.2 Hz), 4.32 (1H, dd, *J* = 11.6, 4.8 Hz), 4.24 (1H, dd, *J* = 11.6, 6.4 Hz), 4.08 (2H, s), 4.02-3.96 (1H, m), 2.81 (2H, t, *J* = 6.8 Hz), 2.24-2.15 (1H, m), 2.08 (3H, s), 1.96-1.86 (1H, m), 1.44 (3H, t, *J* = 7.2 Hz).

**$^{13}$C NMR (100 MHz, $CDCl_3$):** δ 212.8, 201.5, 170.6, 70.5, 65.6, 48.8, 48.0, 36.7, 24.5, 20.7, 13.7.

**ESIHRMS:** Found: m/z 313.0333; Calcd for $C_{11}H_{18}O_4S_2Cl$: $(M+H)^+$ 313.0335.

**9.24. Synthesis of 2,8-bis((ethoxycarbonothioyl)thio)-5-oxononane-1,9-diyl diacetate (3hb) (Table 3, entry 7)**

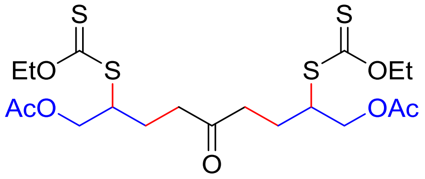


Prepared using xanthate **1h** (89.8 mg, 0.301 mmol) and alkene **2b** (160 μL, 1.48 mmol) with **8** (1.6 mg, 0.00143 mmol) for 52 h.

Purification: Hexane:EtOAc = 4:1

Yield: 103 mg (0.207 mmol, 69% yield) as a yellow oil (dr = 52:48, determined by $^{13}$C NMR analysis as an inseparable mixture of diastereoisomers).

**$^{1}$H NMR (400 MHz, $CDCl_3$):** *for the mixture of diastereoisomers* δ 4.65 (4H, q, $J$ = 7.2 Hz), 4.29 (2H, dd, $J$ = 11.6, 5.2 Hz), 4.23 (2H, dd, $J$ = 11.6, 6.4 Hz), 3.99-3.93 (2H, m), 2.68-2.54 (4H, m), 2.19-2.10 (2H, m), 2.08 (6H, s), 1.92-1.80 (2H, m), 1.43 (6H, t, $J$ = 7.2 Hz).

**$^{13}$C NMR (100 MHz, $CDCl_3$):** *for the mixture of diastereoisomers* δ 212.97, 212.95, 207.9, 170.6, 70.4, 65.6, 49.02, 48.99, 39.7, 24.5, 20.8, 13.7.

**ESIHRMS:** *for the mixture of diastereoisomers* Found: m/z 499.0954; Calcd for $C_{19}H_{31}O_7S_4$: $(M+H)^+$ 499.0953.

**9.25. Synthesis of 4-(1,3-dioxoisoindolin-2-yl)-2-((ethoxycarbonothioyl)thio)butyl acetate (3ib)** [11] **(Table 3, entry 8)**

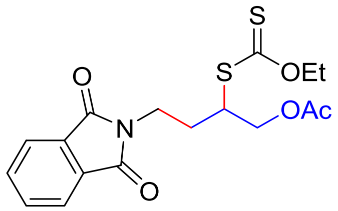


Prepared using xanthate **1i** (97.0 mg, 0.300 mmol) and alkene **2b** (70 μL, 0.649 mmol) with **8** (1.8 mg, 0.00160 mmol) for 41 h.

Purification: Hexane:EtOAc = 5:1

Yield: 91.2 mg (0.239 mmol, 80% yield) as a pale yellow oil.

**$^{1}$H NMR (400 MHz, $CDCl_3$):** δ 7.85 (2H, dd, *J* = 5.6, 3.2 Hz), 7.73 (2H, dd, *J* = 5.6, 3.2 Hz), 4.62 (2H, q, *J* = 7.2 Hz), 4.42 (1H, dd, *J* = 11.6, 4.8 Hz), 4.28 (1H, dd, *J* = 11.6, 6.0 Hz), 4.02-3.96 (1H, m), 3.92-3.80 (2H, m), 2.23-2.15 (1H, m), 2.12-2.01 (3H, s + 1H, m), 1.40 (3H, t, *J* = 7.2 Hz).

**$^{13}$C NMR (100 MHz, $CDCl_3$):** δ 212.6, 170.6, 168.2, 134.0, 132.0, 123.3, 70.3, 65.1, 46.9, 35.6, 29.8, 20.7, 13.7.

**9.26. Synthesis of 4-(2,5-dioxopyrrolidin-1-yl)-2-((ethoxycarbonothioyl)thio)butyl acetate (3jb) (Table 3, entry 9)**

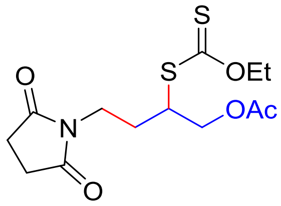


Prepared using xanthate **1j** (69.9 mg, 0.300 mmol) and alkene **2b** (70 μL, 0.649 mmol) with **8** (1.8 mg, 0.00160 mmol) for 24 h.

Purification: Hexane:$Et_2O$ = 1:2

Yield: 56.4 mg (0.169 mmol, 56% yield) as a yellow oil.

**$^{1}$H NMR (400 MHz, $CDCl_3$):** δ 4.64 (2H, q, *J* = 7.2 Hz), 4.40 (1H, dd, *J* = 11.6, 4.8 Hz), 4.25 (1H, dd, *J* = 11.6, 5.6 Hz), 3.95-3.89 (1H, m), 3.74-3.62 (2H, m), 2.73 (4H, s), 2.13-2.04 (3H, s + 1H, m), 2.00-1.91 (1H, m), 1.43 (3H, t, *J* = 7.2 Hz).

**$^{13}$C NMR (100 MHz, $CDCl_3$):** δ 213.0, 177.1, 170.6, 70.4, 64.9, 47.1, 36.3, 28.8, 28.2, 20.7, 13.7.

**ESIHRMS:** Found: m/z 334.0786; Calcd for $C_{13}H_{20}NO_5S_2$: $(M+H)^+$ 334.0783.

**9.27. Synthesis of 4-acetamido-2-((ethoxycarbonothioyl)thio)-5,5,5-trifluoro-pentyl acetate (3kb)** [12] **(Table 3, entry 10)**

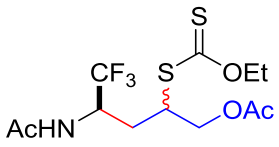


Prepared using xanthate **1k** (78.5 mg, 0.300 mmol) and alkene **2b** (70 μL, 0.649 mmol) with **8** (1.6 mg, 0.00143 mmol) for 24 h.

Purification: Hexane:EtOAc = 3:1

Yield: 79.8 mg (0.221 mmol, 74% yield) as a pale yellow oil (dr = 63:37, determined by $^{19}$F NMR analysis. Two diastereomers were separated partially for characterization).

**$^{1}$H NMR (400 MHz, $CDCl_3$):** δ 6.03-5.97 (1H + 1H×0.57, s br), 4.95-4.78 (1H + 1H×0.57, m), 4.69-4.62 (2H, q, *J* = 7.2 Hz + 2H×0.57, q, *J* = 7.2 Hz), 4.43 (1H×0.57, dd, *J* = 12.0, 4.4 Hz), 4.32-4.25 (2H, m + 1H×0.57, dd, *J* = 12.0, 4.8 Hz), 4.18-4.11 (1H, m), 4.00-3.94 (1H×0.57, m), 2.31-2.24 (1H×0.57, m), 2.20-1.93 (3H, s + 3H, s + 2H, m + 3H×0.57, s + 3H×0.57, s + 1H×0.57, m), 1.45-1.40 (3H, t, *J* = 7.2 Hz + 3H×0.57, t, *J* = 7.2 Hz).

**$^{19}$F NMR (377 MHz, $CDCl_3$):** δ -76.0 (3F, d, *J* = 7.5 Hz), -76.1 (3F×0.57, d, *J* = 7.5 Hz).

Major-**3kb**

**$^{1}$H NMR (400 MHz, $CDCl_3$):** δ 6.00 (1H, s br), 4.94-4.88 (1H, m), 4.64 (2H, q, *J* = 7.2 Hz), 4.33-4.25 (2H, m), 4.18-4.11 (1H, m), 2.19-2.12 (1H, m), 2.10 (3H, s), 2.04 (3H, s), 2.02-1.95 (1H, m), 1.42 (3H, t, *J* = 7.2 Hz).

**$^{13}$C NMR (100 MHz, $CDCl_3$):** δ 212.3, 170.7, 170.0, 124.9 (q, *J* = 280.4 Hz), 70.8, 65.8, 48.3 (q, *J* = 30.8 Hz), 45.9, 29.2, 23.0, 20.7, 13.6.

**$^{19}$F NMR (377 MHz, $CDCl_3$):** δ -76.0 (3F, d, *J* = 7.5 Hz).

Minor-**3kb**

**$^{1}$H NMR (400 MHz, $CDCl_3$):** δ 5.88 (1H, s br), 4.86-4.78 (1H, m), 4.66 (2H, q, *J* = 7.2 Hz), 4.43 (1H, dd, *J* = 12.0, 4.4 Hz), 4.29 (1H, dd, *J* = 12.0, 4.8 Hz), 4.00-3.94

(1H, m), 2.31-2.24 (1H, m), 2.12 (3H, s), 2.10 (3H, s), 2.01-1.93 (1H, m), 1.43 (3H, t, *J* = 7.2 Hz).

**$^{13}$C NMR (100 MHz, $CDCl_3$):** δ 213.2, 170.4, 170.2, 124.7 (q, *J* = 280.3 Hz), 70.7, 63.9, 48.5 (q, *J* = 30.8 Hz), 45.6, 30.4, 23.1, 20.6, 13.7.

**$^{19}$F NMR (377 MHz, $CDCl_3$):** δ -76.2 (d, *J* = 7.2 Hz).

## 10. $^{1}$H and $^{13}$C NMR spectra for new compounds

$^{1}$H NMR Spectrum of **2p** (400 MHz, $CDCl_3$)

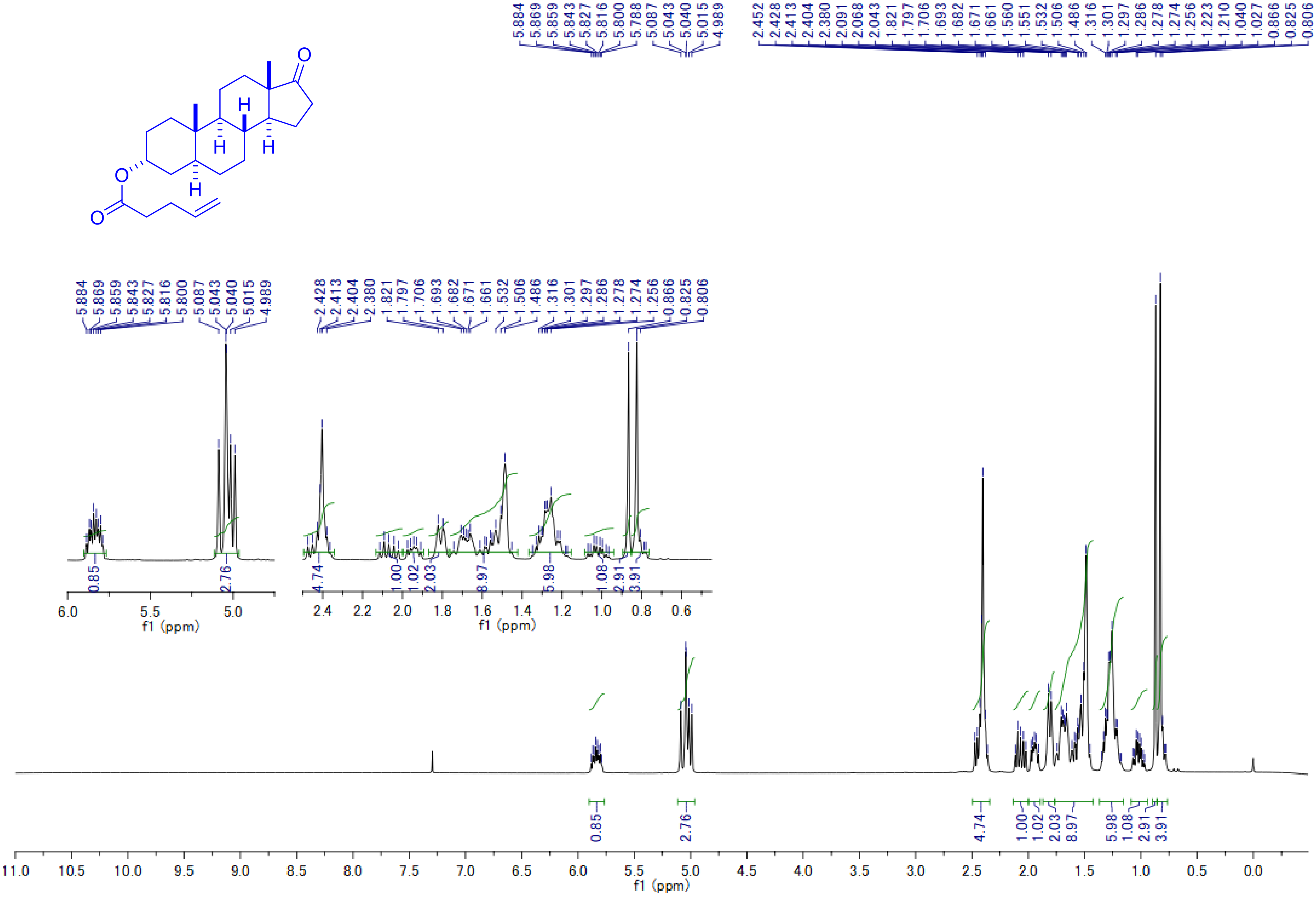

$^{13}C$ NMR Spectrum of **2p** (100 MHz, $CDCl_3$)

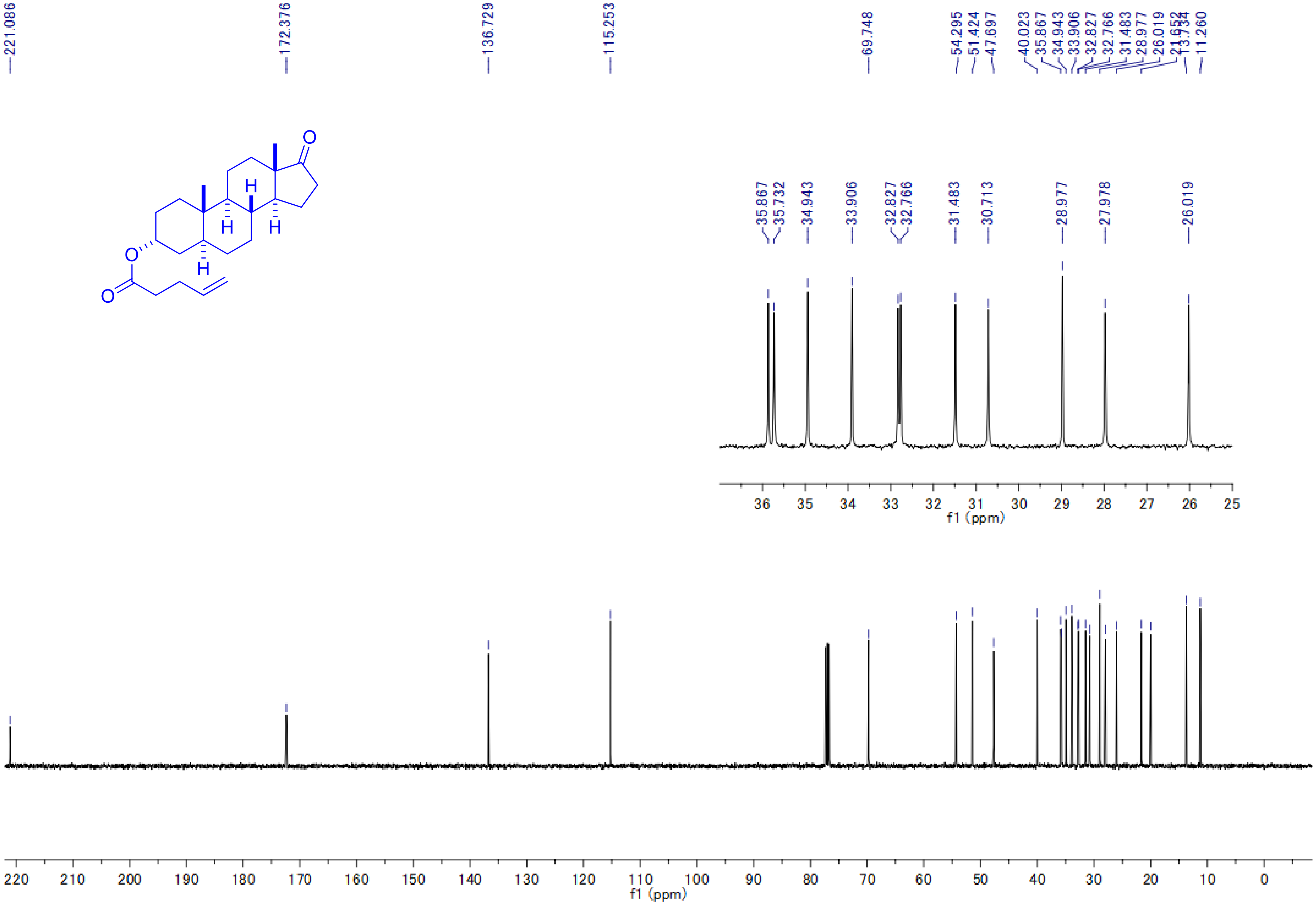

$^{1}$H NMR Spectrum of **2q** (400 MHz, $CDCl_3$)

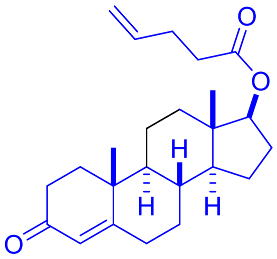

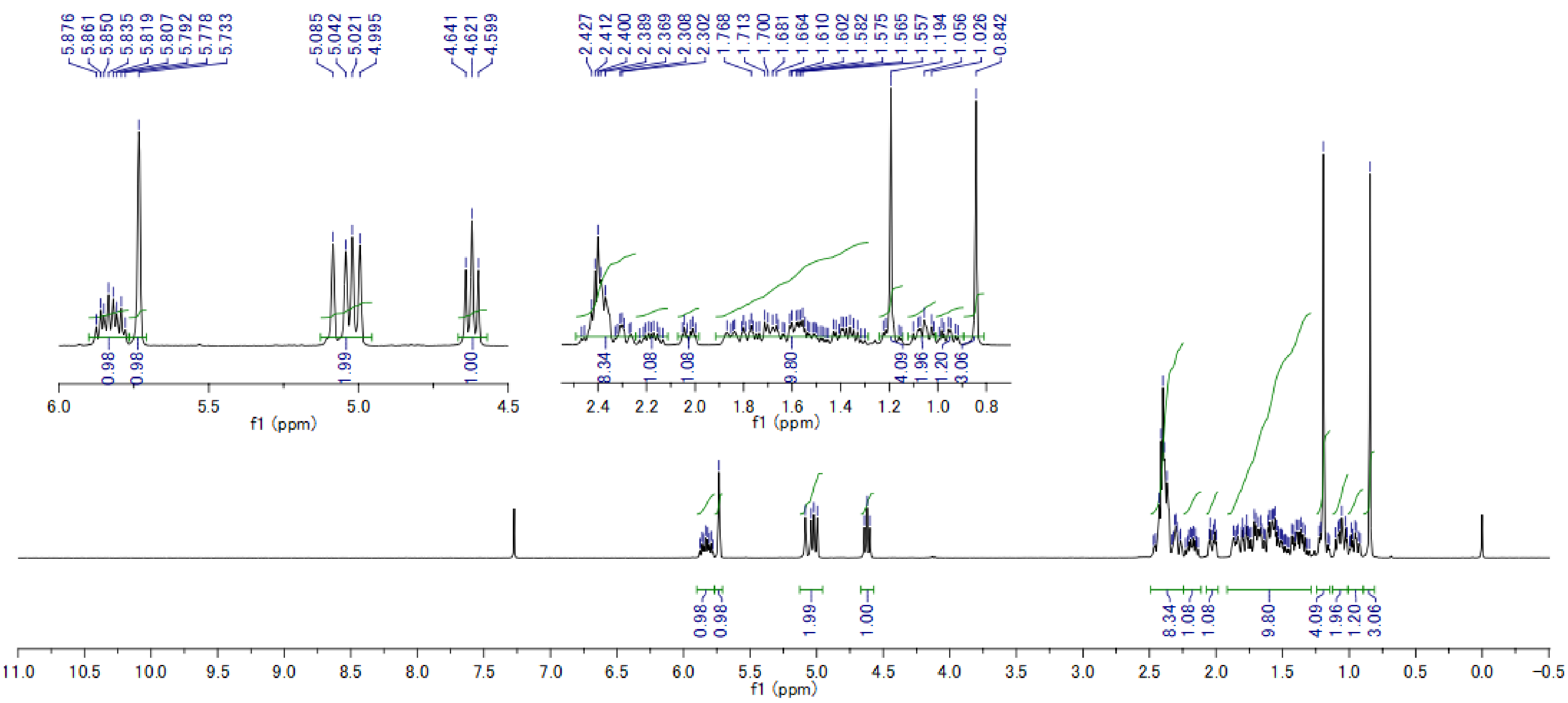

$^{13}$C NMR Spectrum of **2q** (100 MHz, $CDCl_3$)

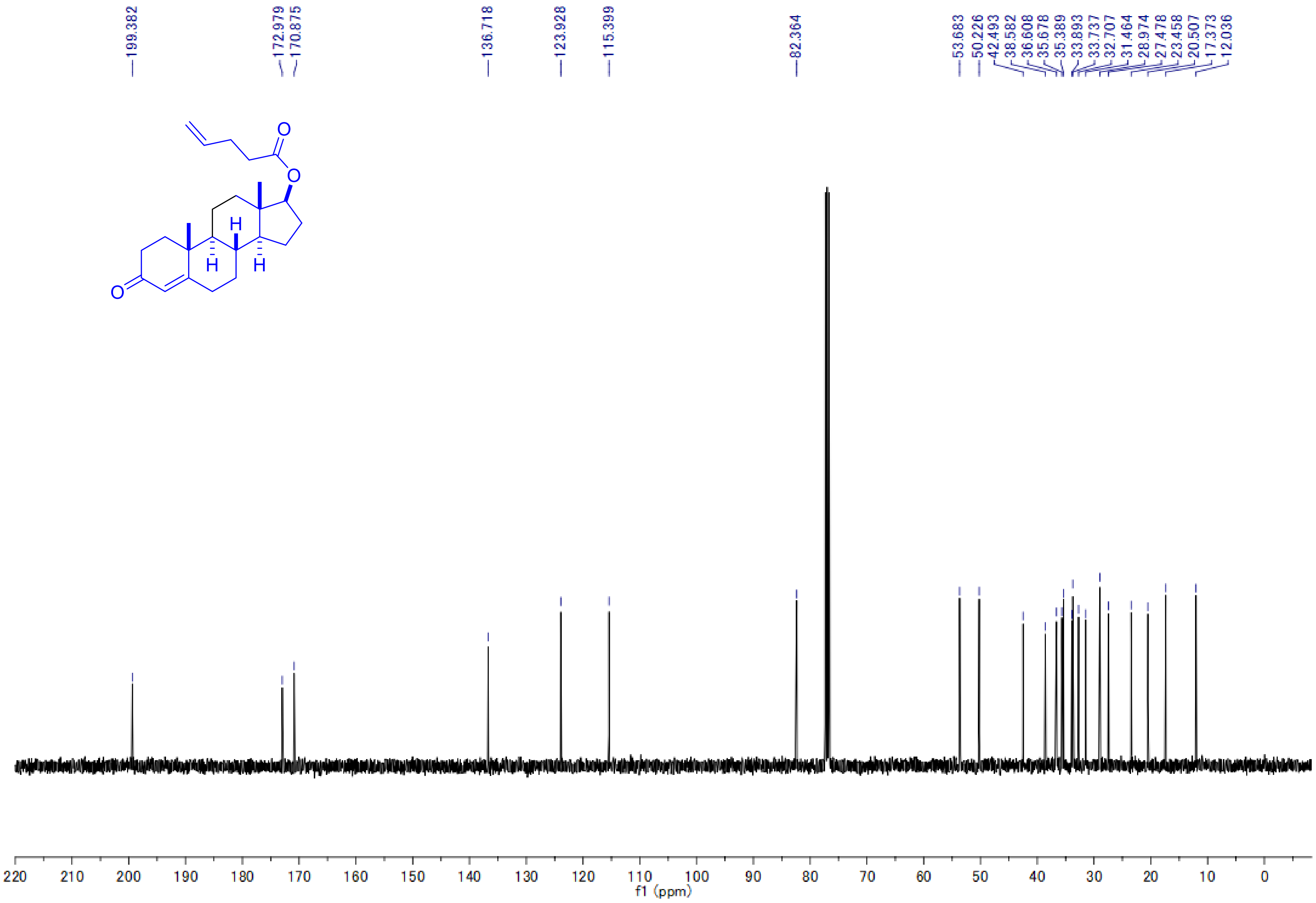

$^{1}$H NMR Spectrum of **3aa** (400 MHz, $CDCl_3$)

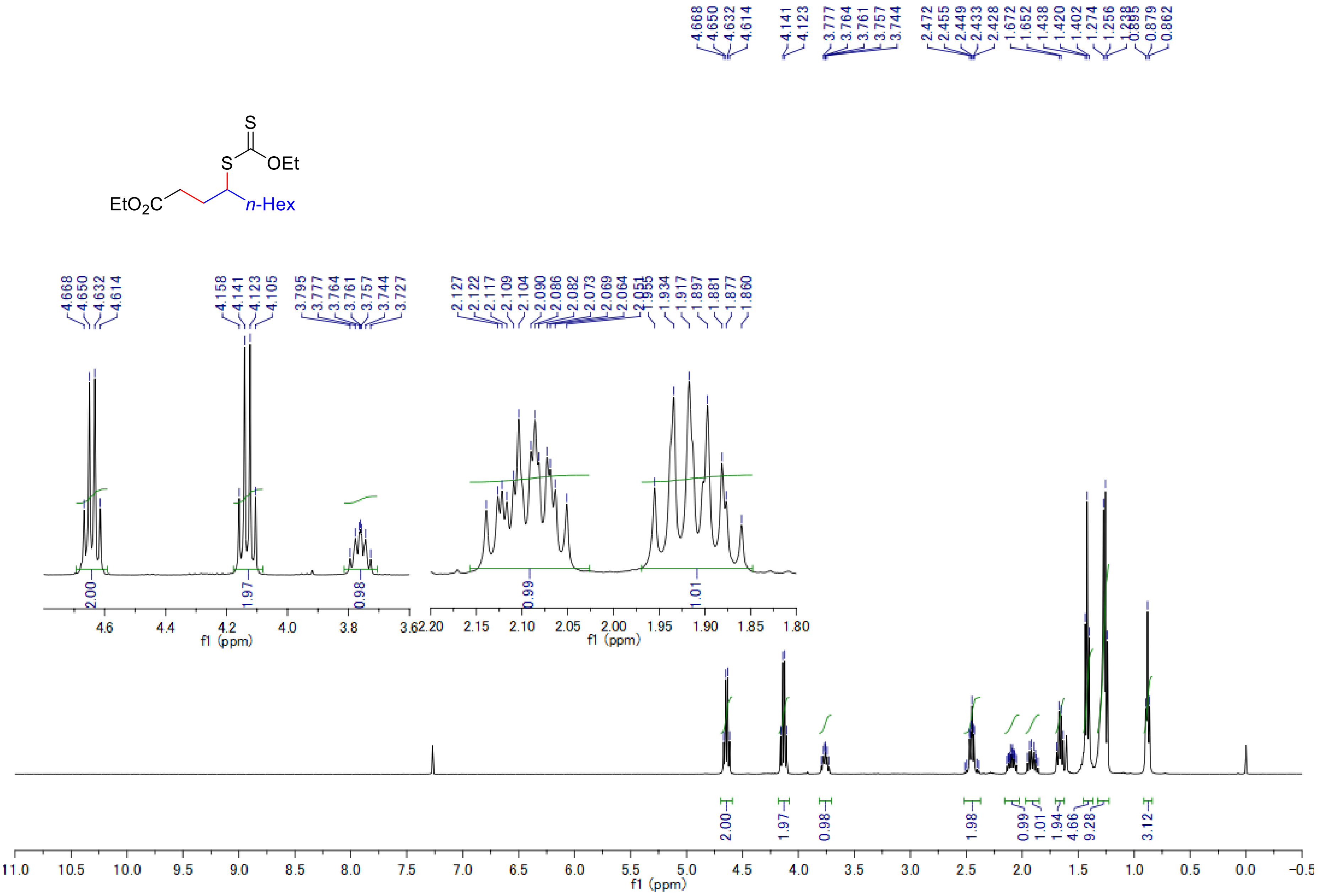

$^{13}$C NMR Spectrum of **3aa** (100 MHz, $CDCl_3$)

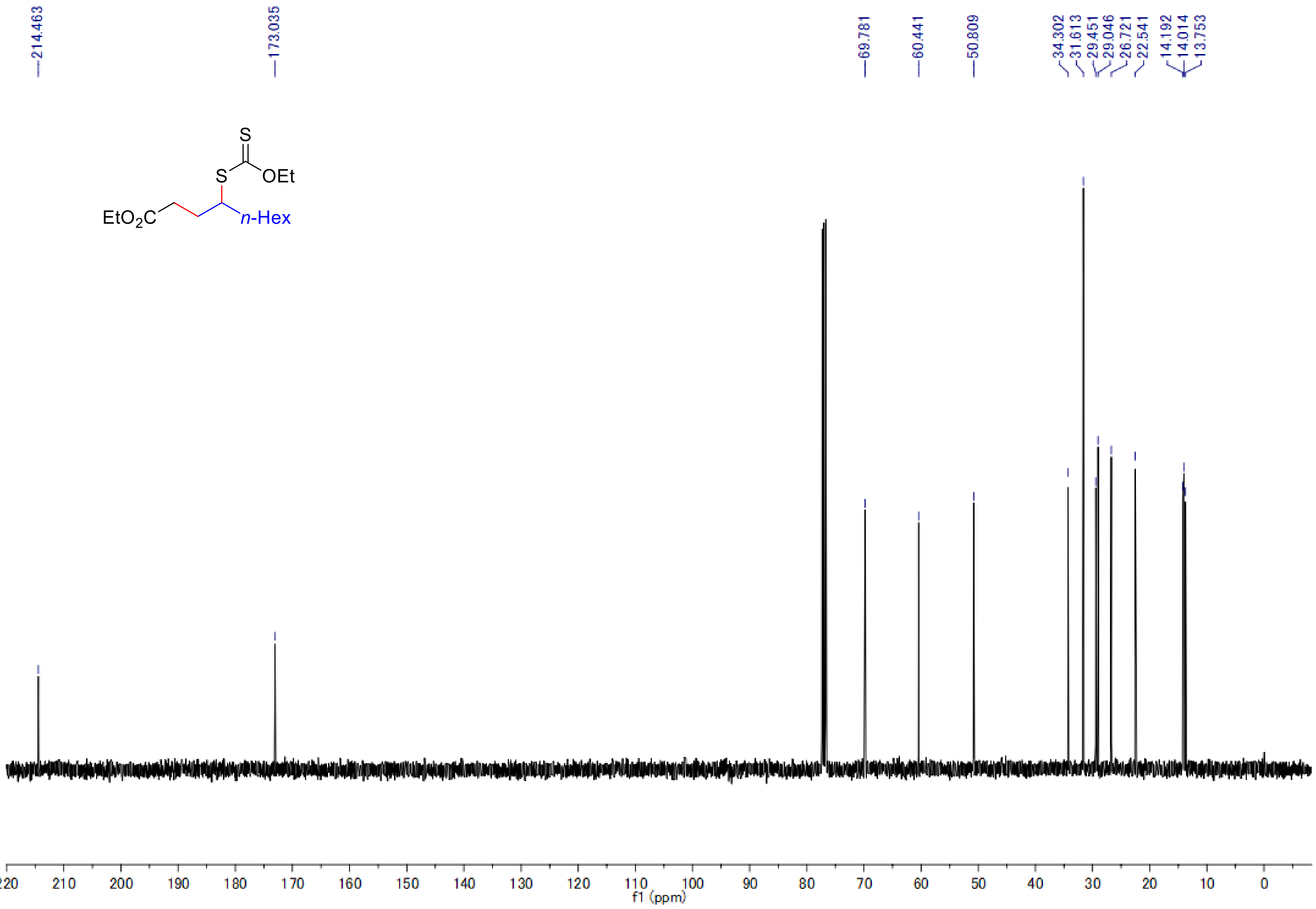

$^{1}$H NMR Spectrum of **3ab** (400 MHz, $CDCl_3$)

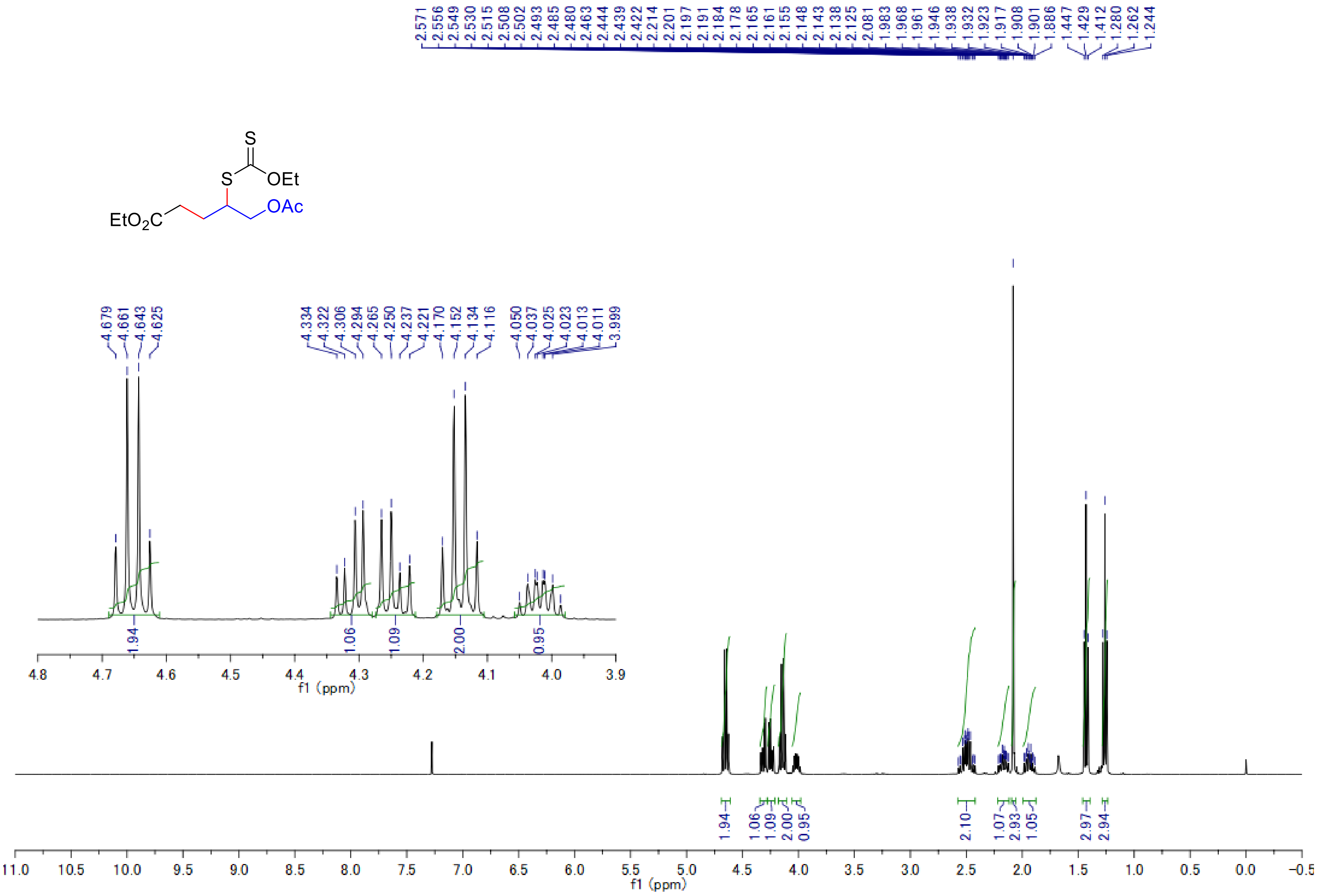

$^{13}$C NMR Spectrum of **3ab** (100 MHz, $CDCl_3$)

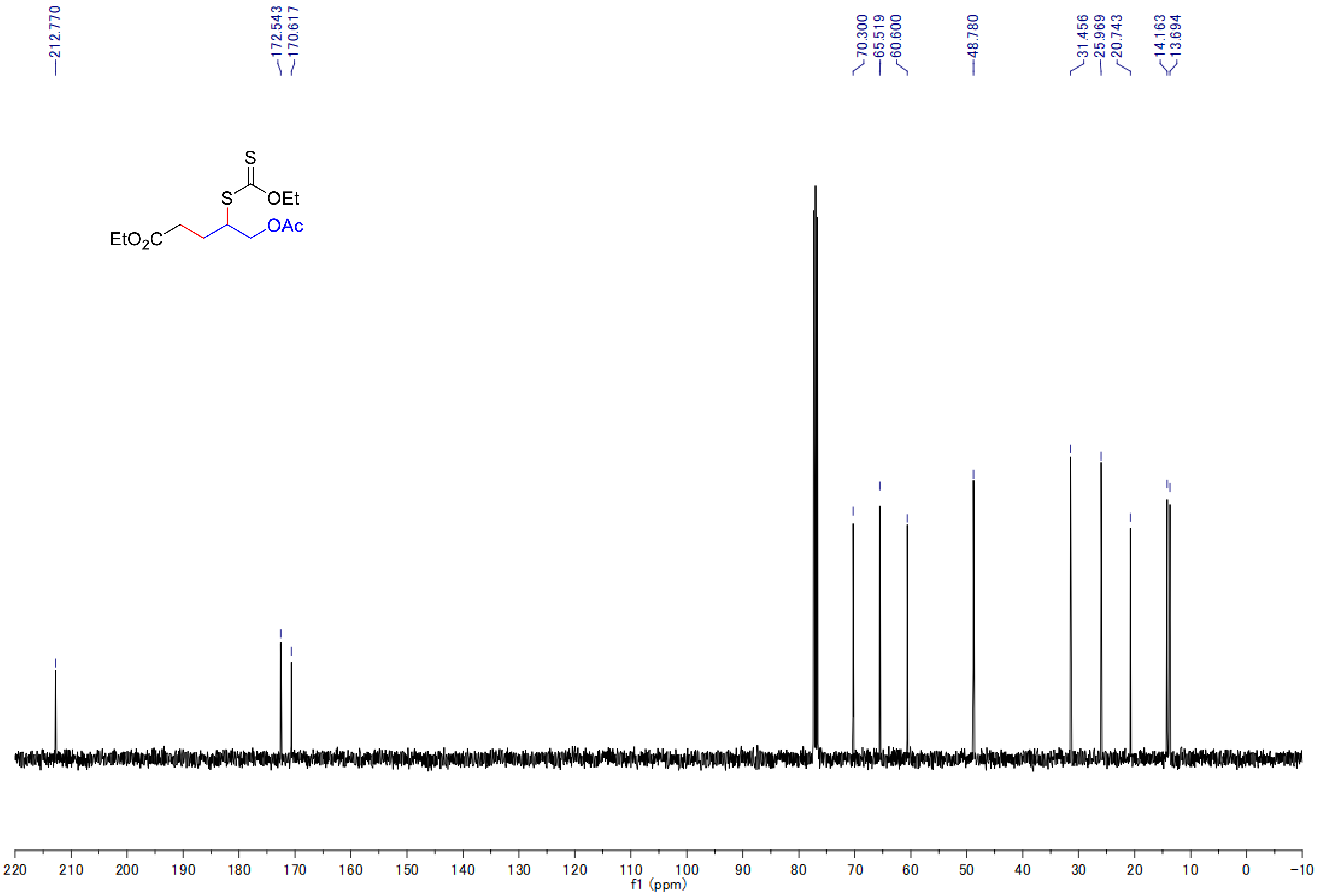

$^{1}$H NMR Spectrum of **3ac** (400 MHz, $CDCl_3$)

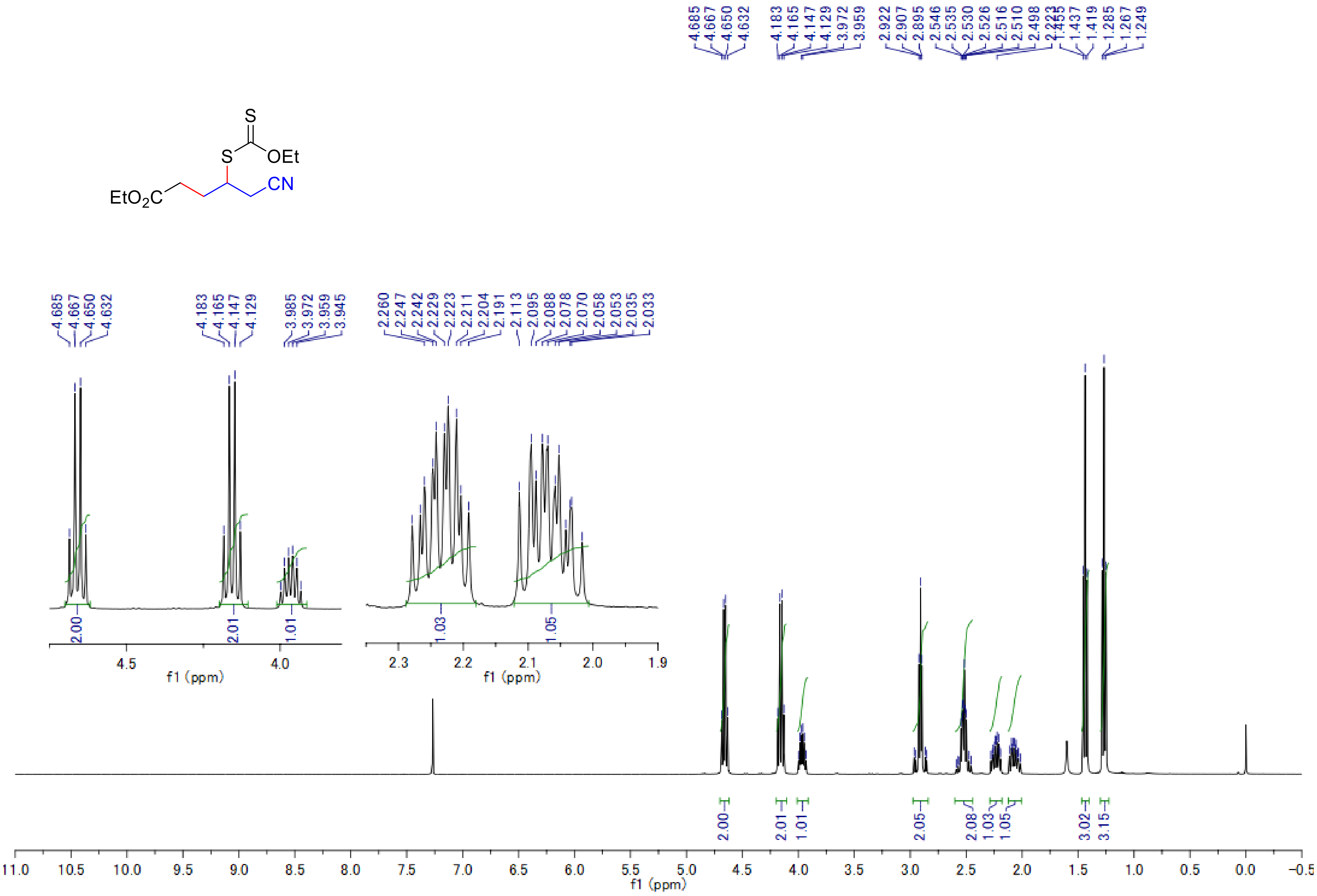

$^{13}$C NMR Spectrum of **3ac** (100 MHz, $CDCl_3$)

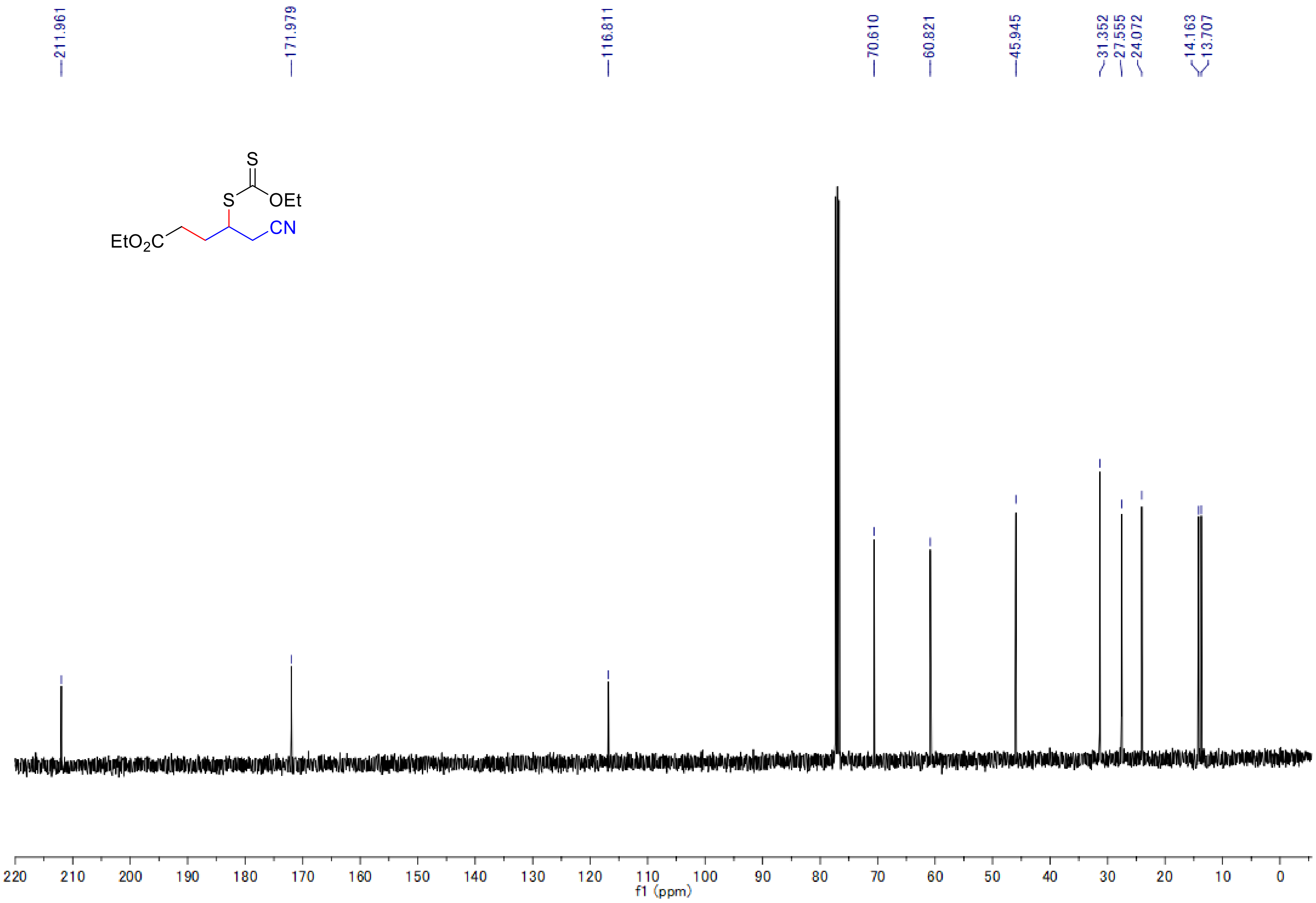

$^{1}$H NMR Spectrum of **3ad** (400 MHz, $CDCl_3$)

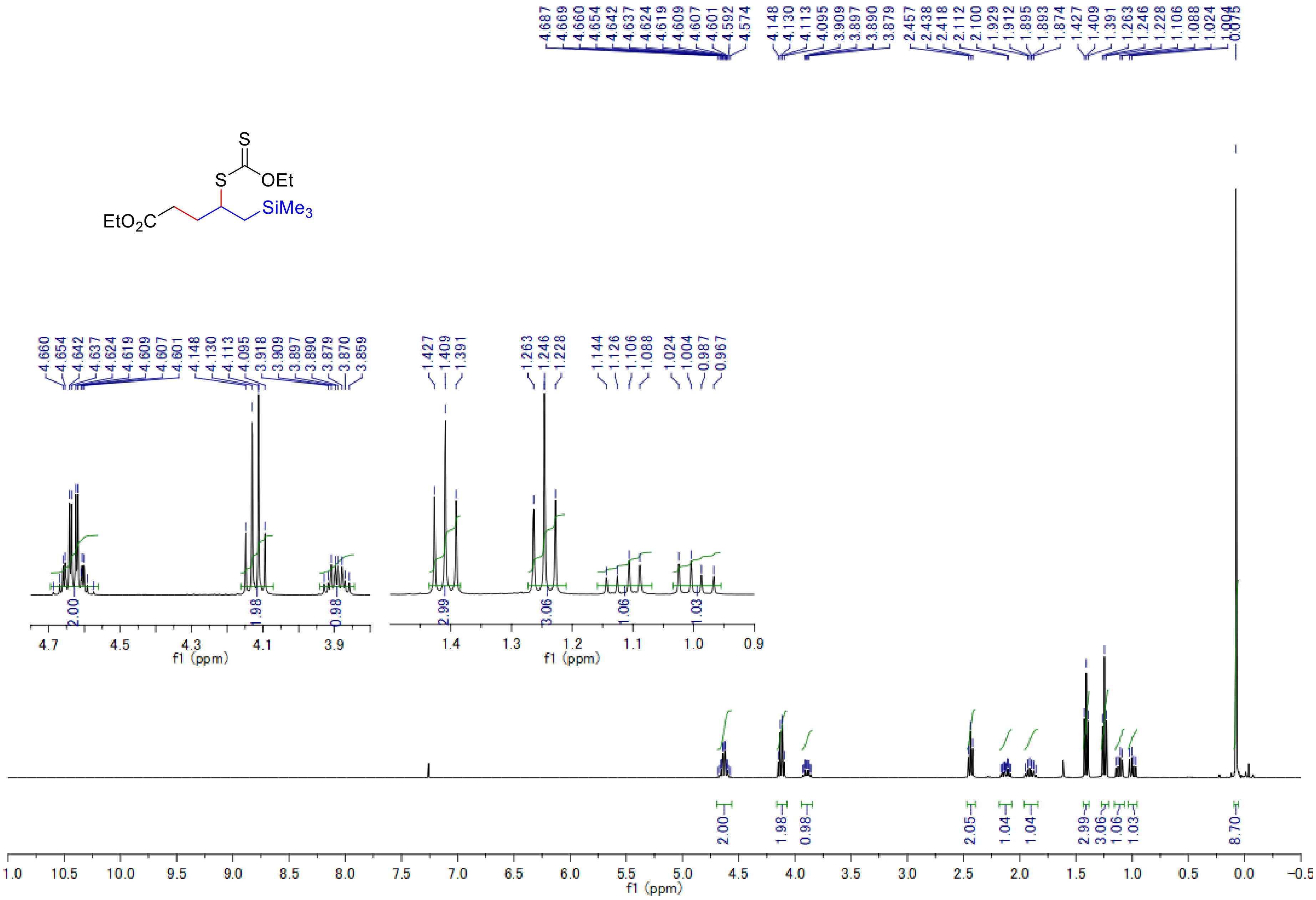

$^{13}C$ NMR Spectrum of **3ad** (100 MHz, $CDCl_3$)

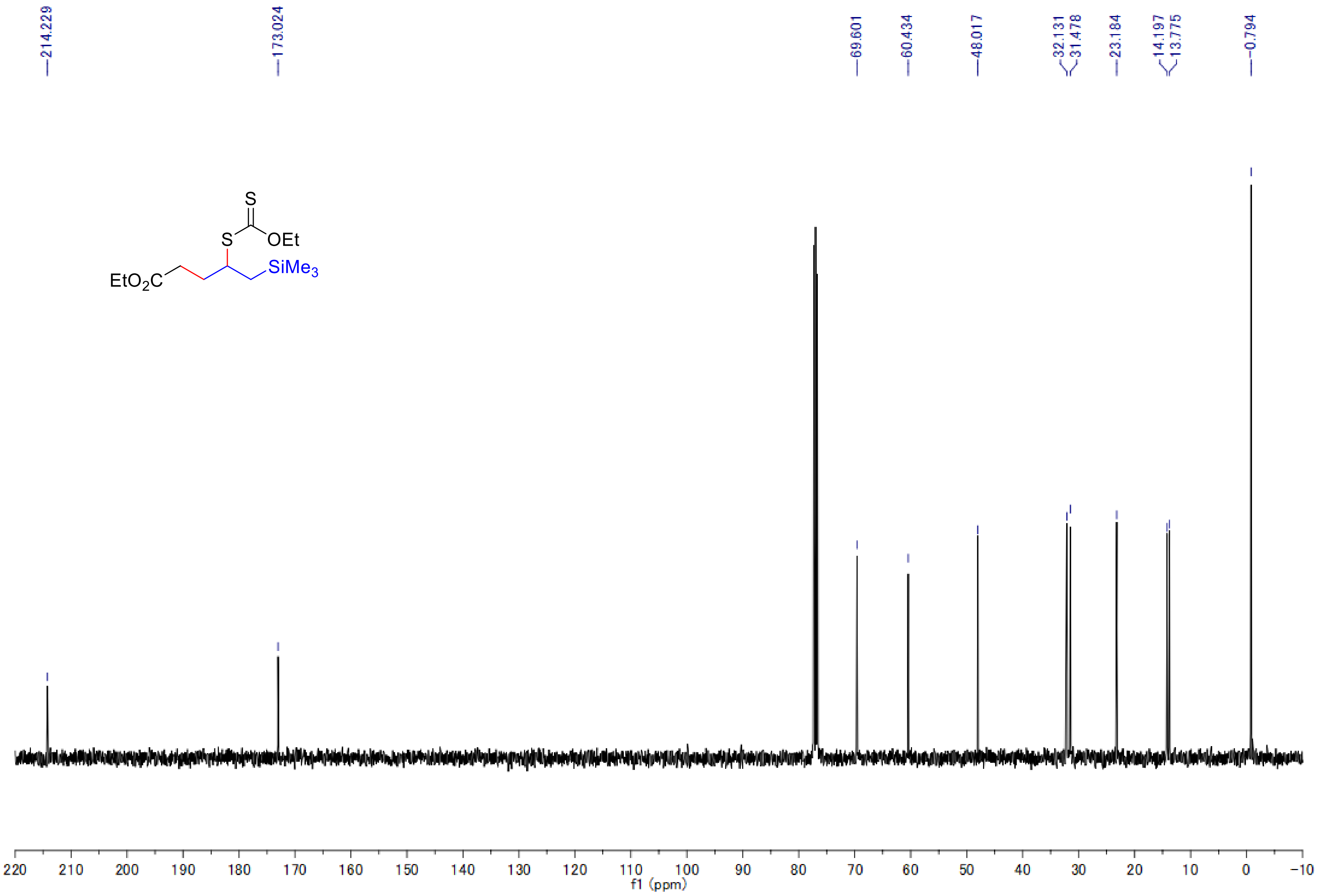

$^{1}$H NMR Spectrum of **3ae** (400 MHz, $CDCl_3$)

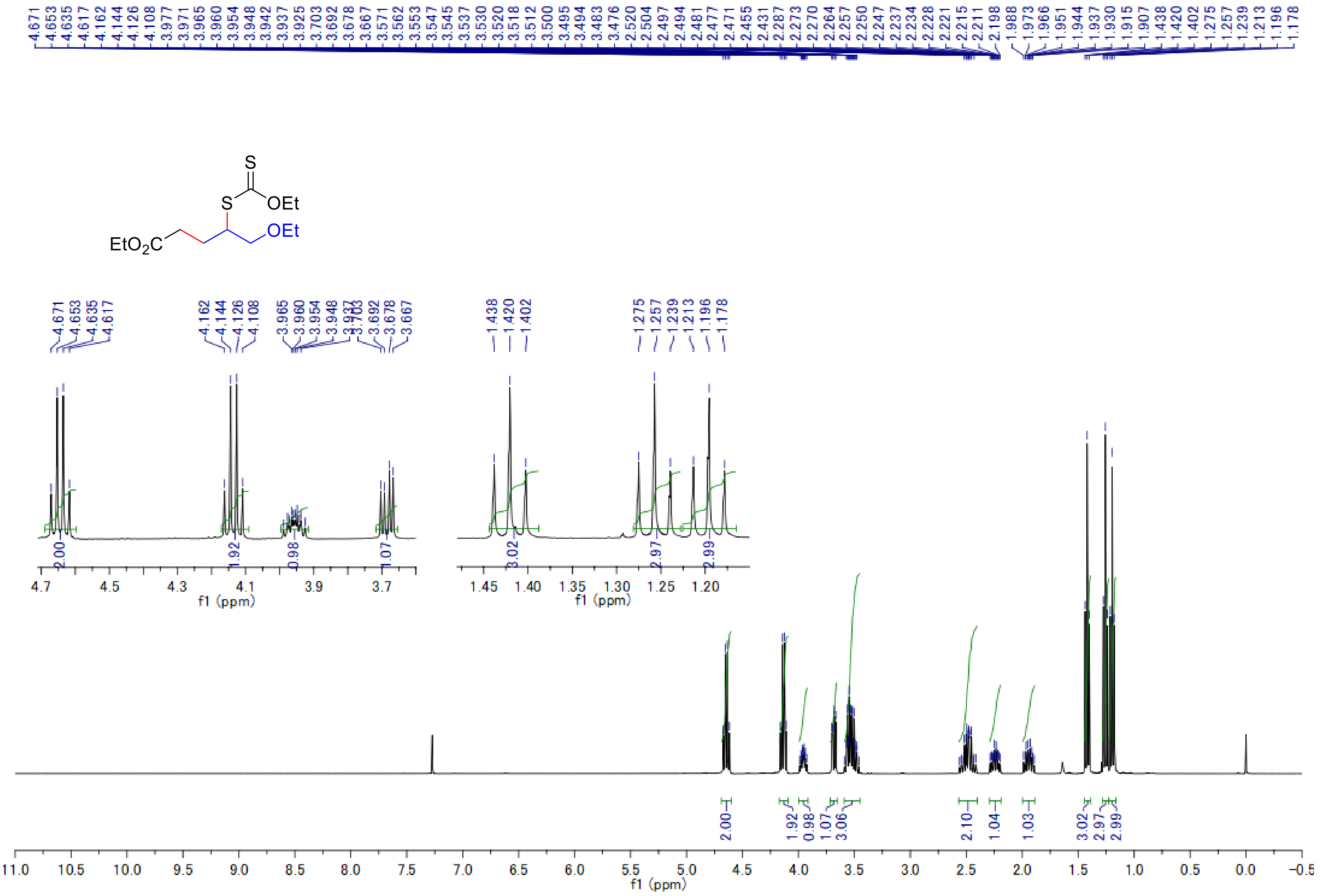

$^{13}$C NMR Spectrum of **3ae** (100 MHz, $CDCl_3$)

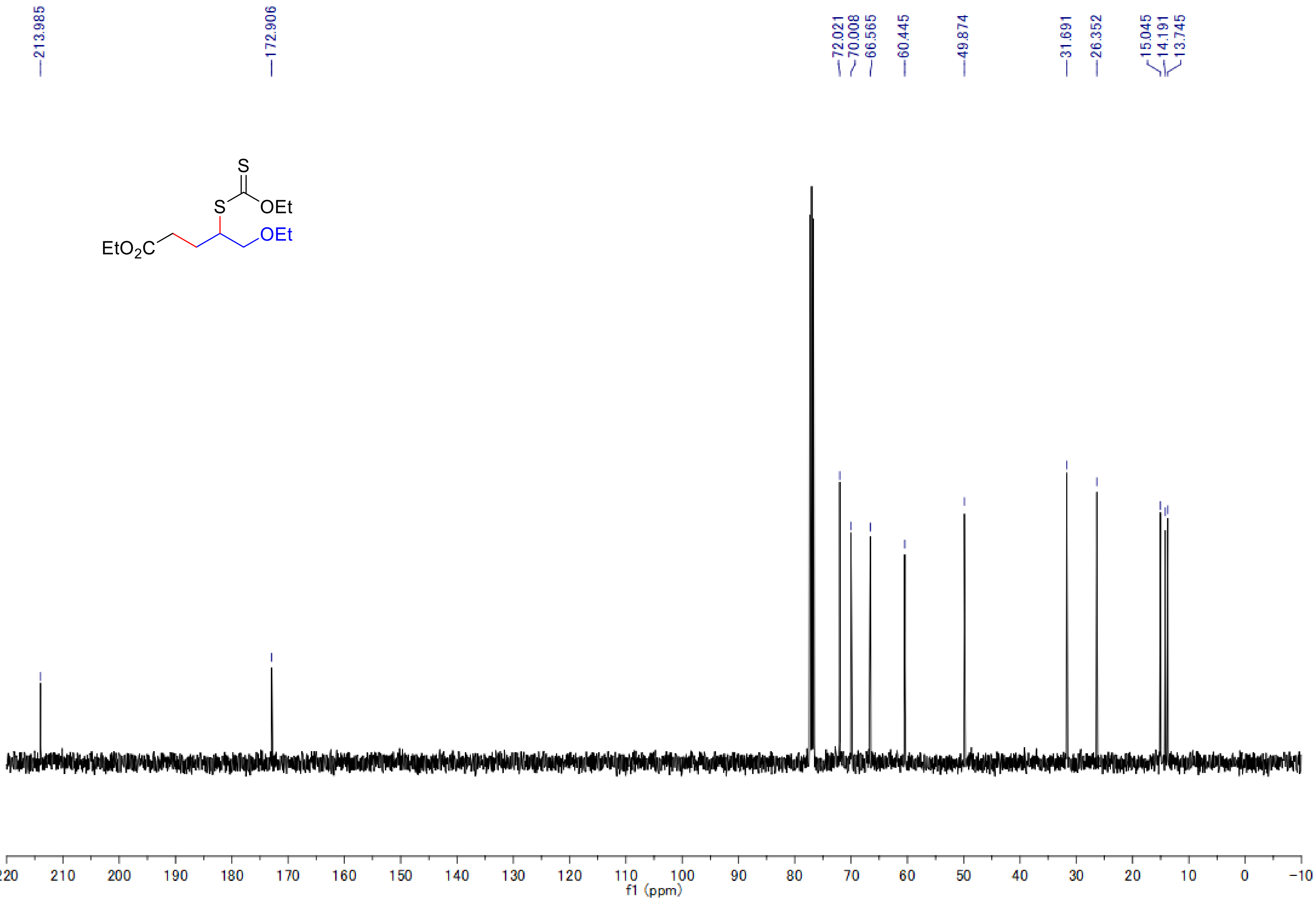

$^{1}$H NMR Spectrum of **3af** (400 MHz, $CDCl_3$)

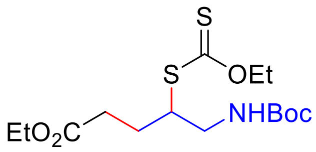


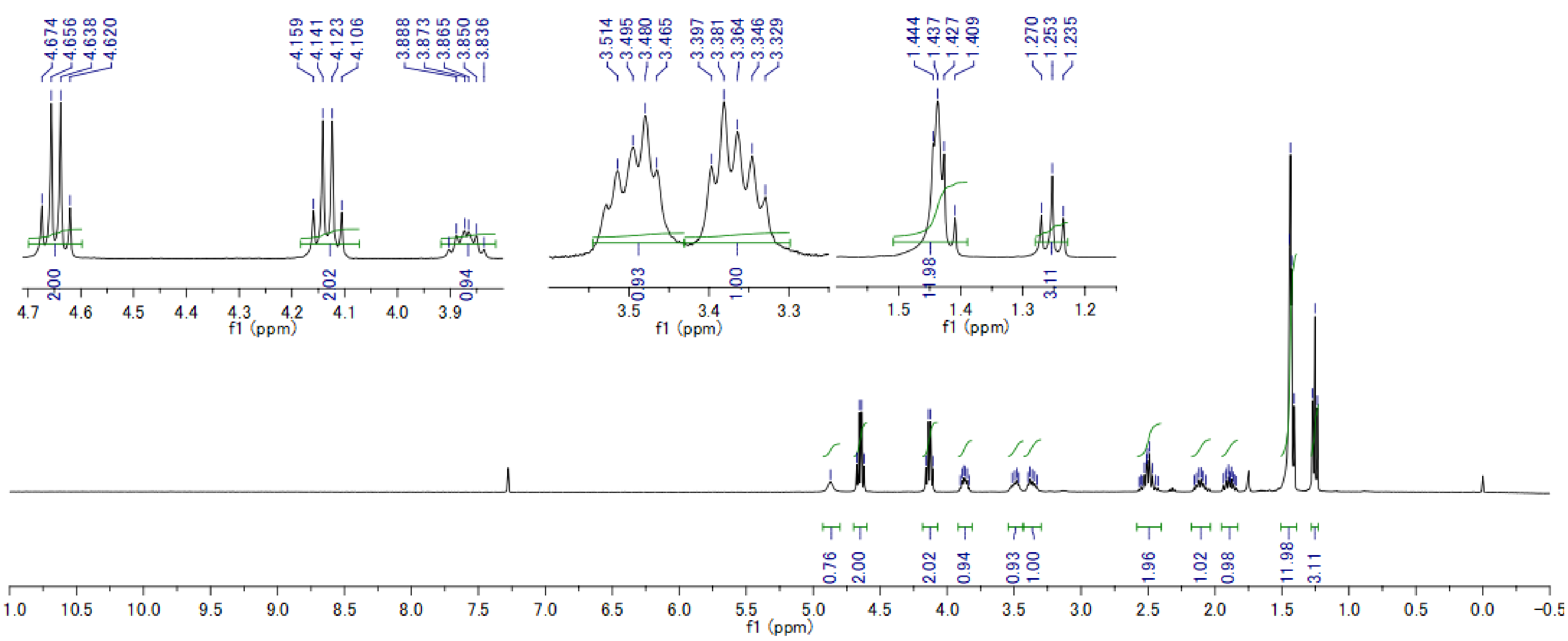

$^{13}C$ NMR Spectrum of **3af** (100 MHz, $CDCl_3$)

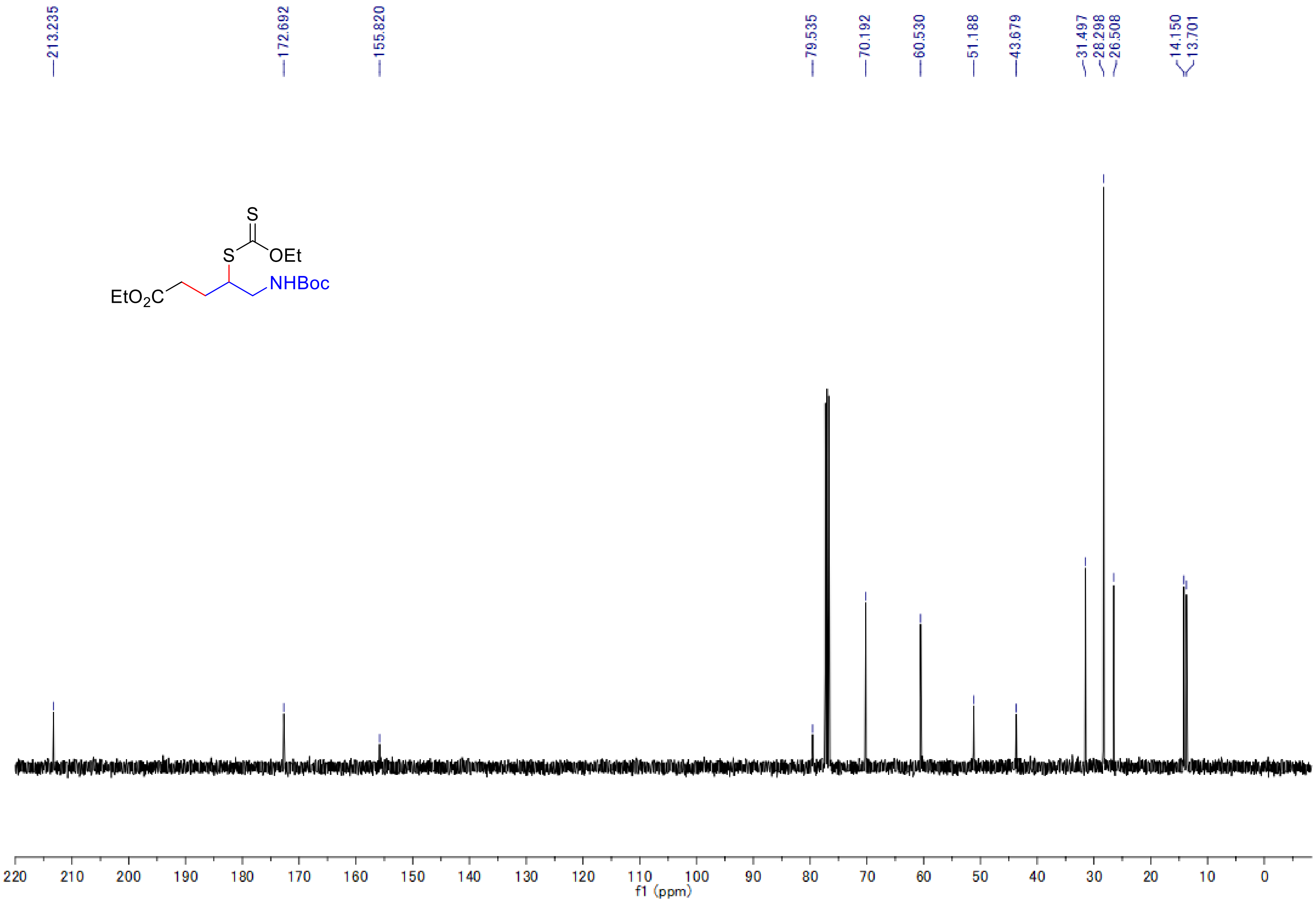

$^{1}$H NMR Spectrum of **3ag** (400 MHz, $CDCl_3$)

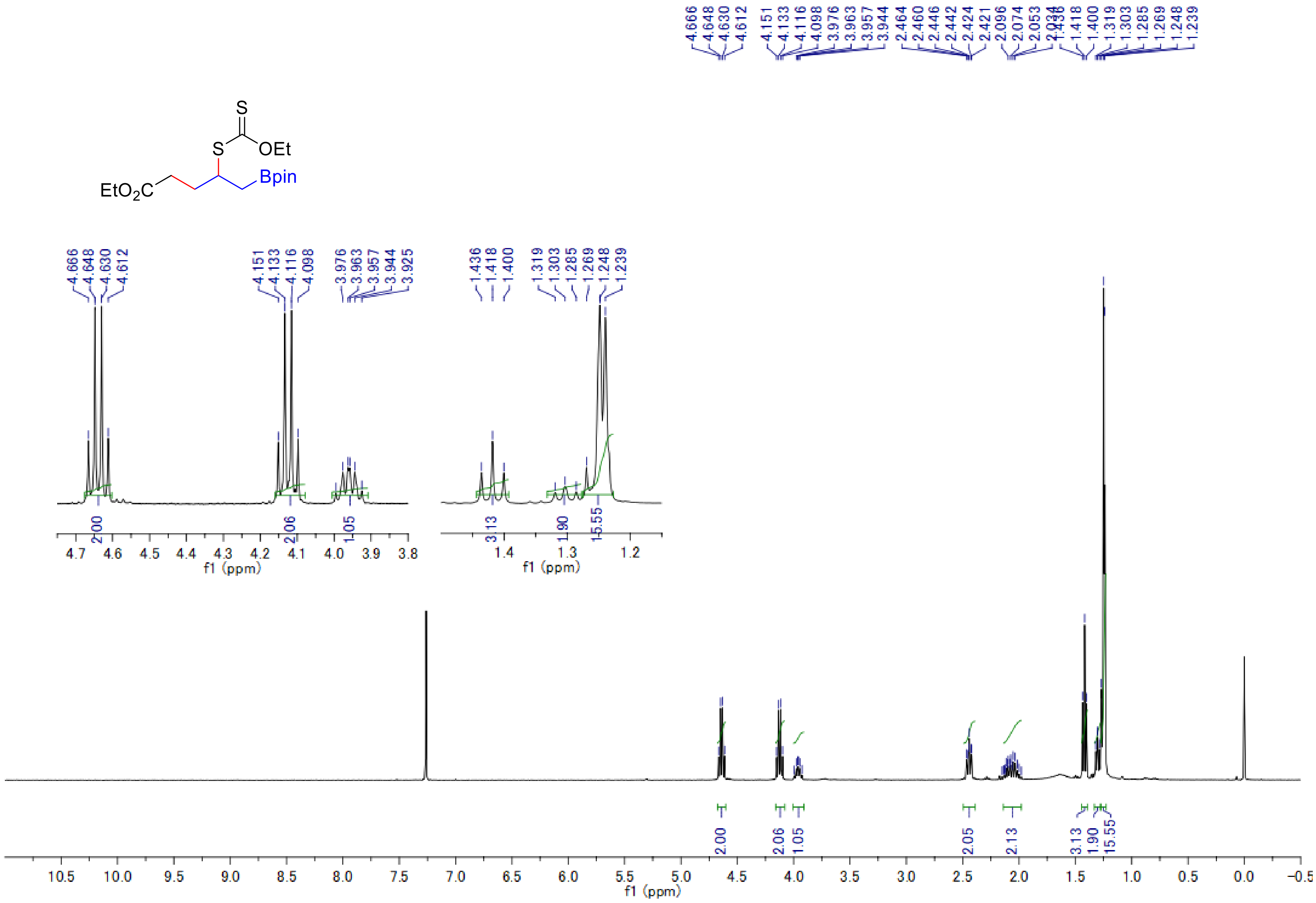

$^{13}C$ NMR Spectrum of **3ag** (100 MHz, $CDCl_3$)

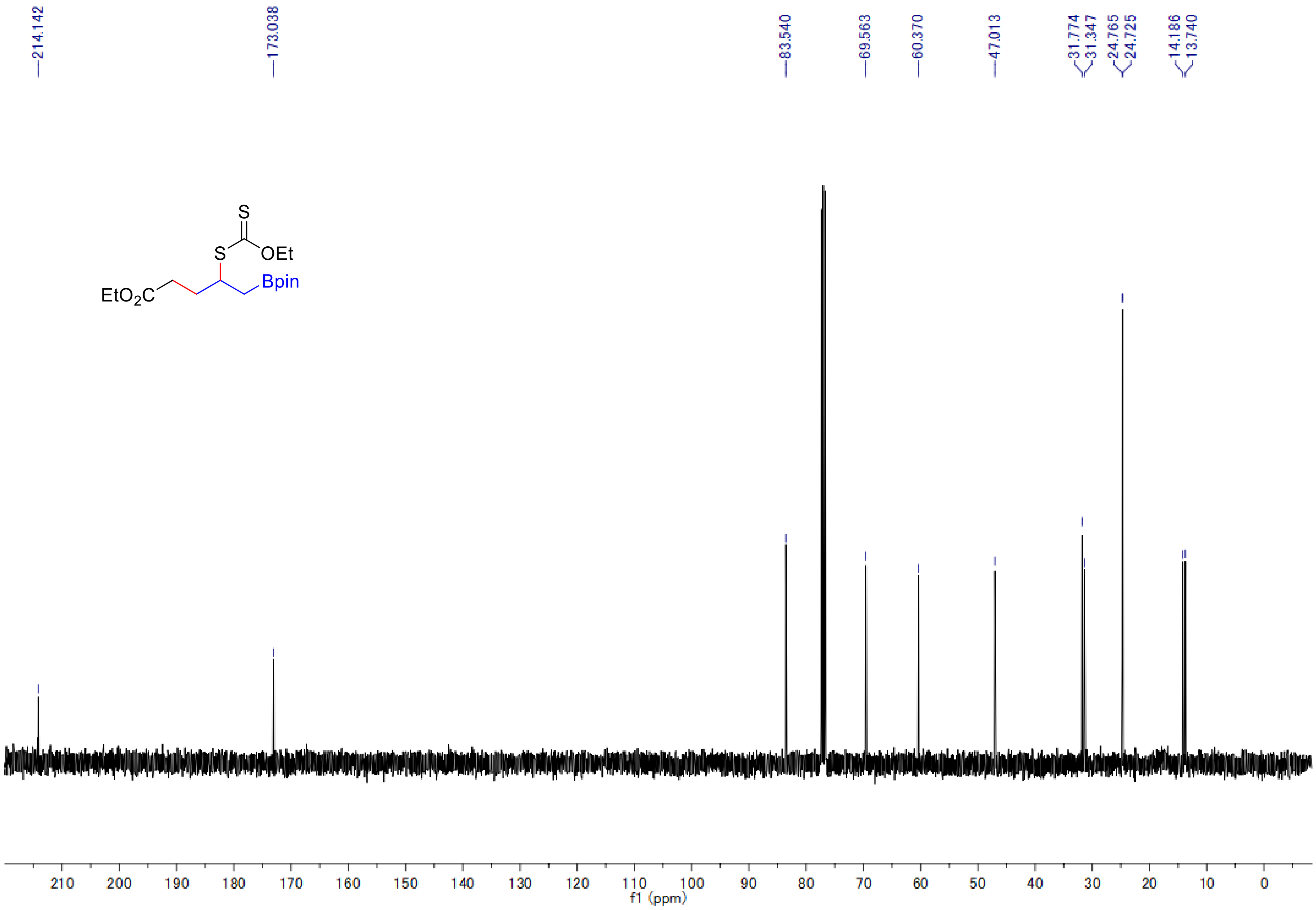

$^{1}$H NMR Spectrum of **3ah** (400 MHz, $CDCl_3$)

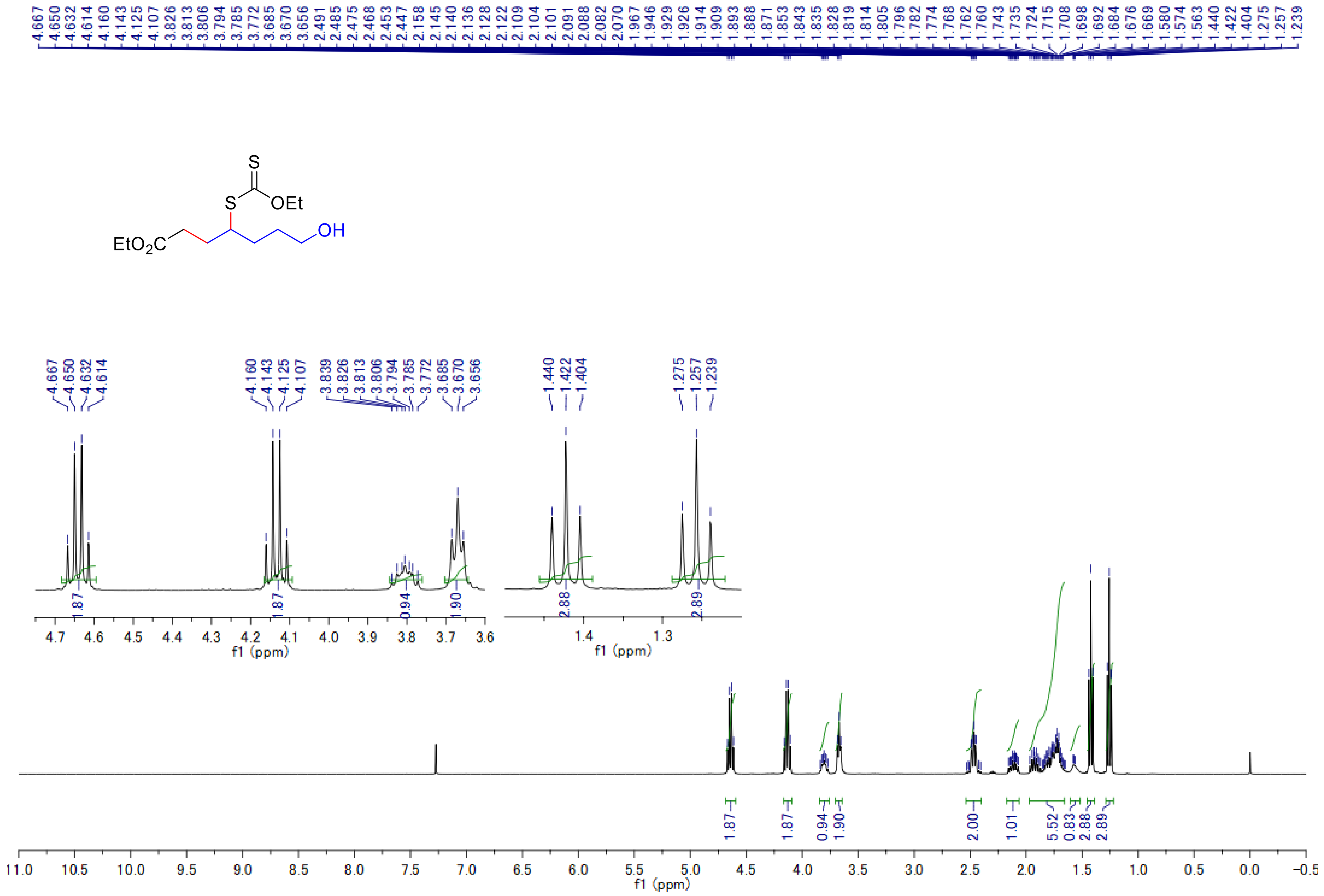

$^{13}$C NMR Spectrum of **3ah** (100 MHz, $CDCl_3$)

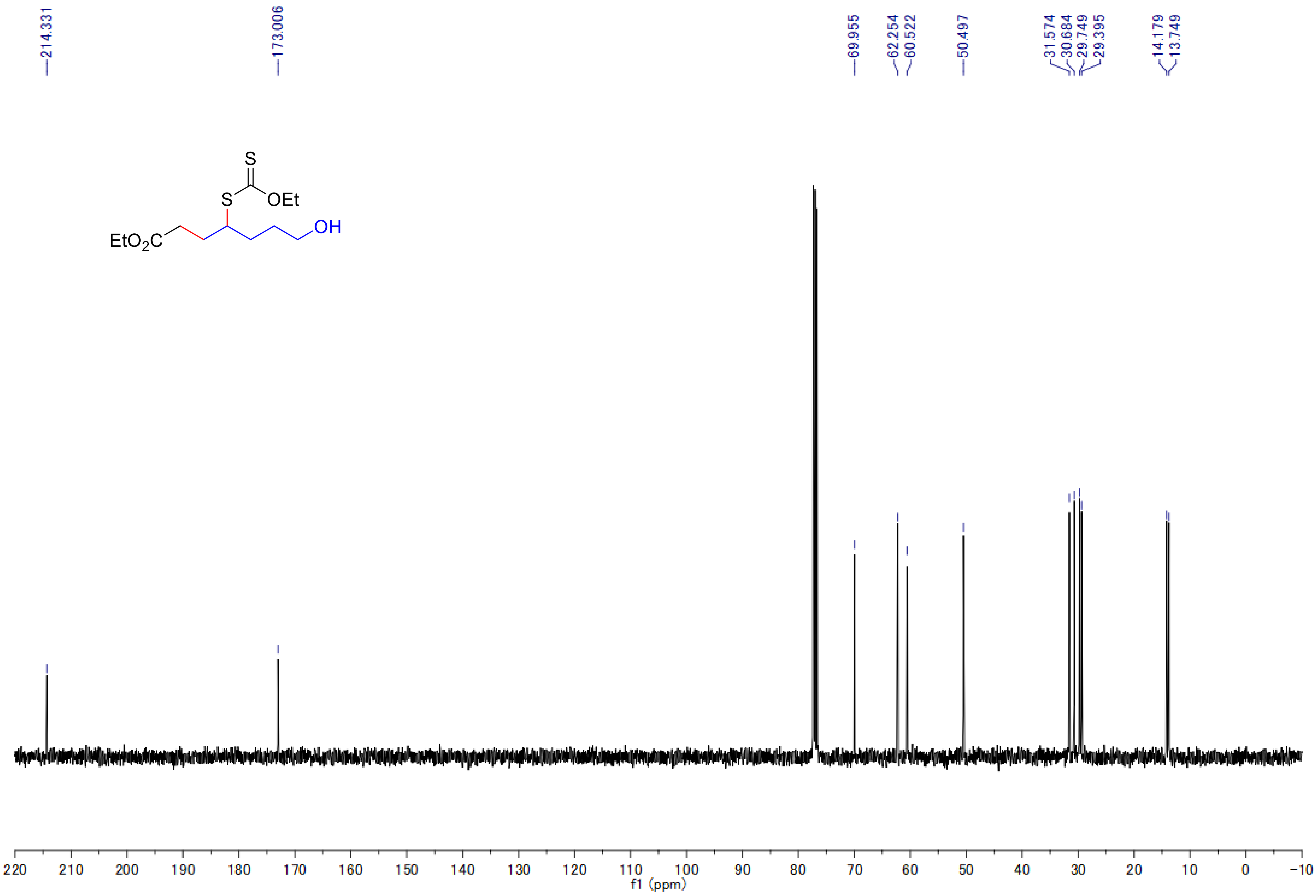

$^{1}$H NMR Spectrum of **3ai** (400 MHz, $CDCl_3$)

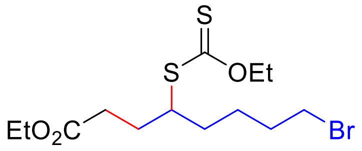


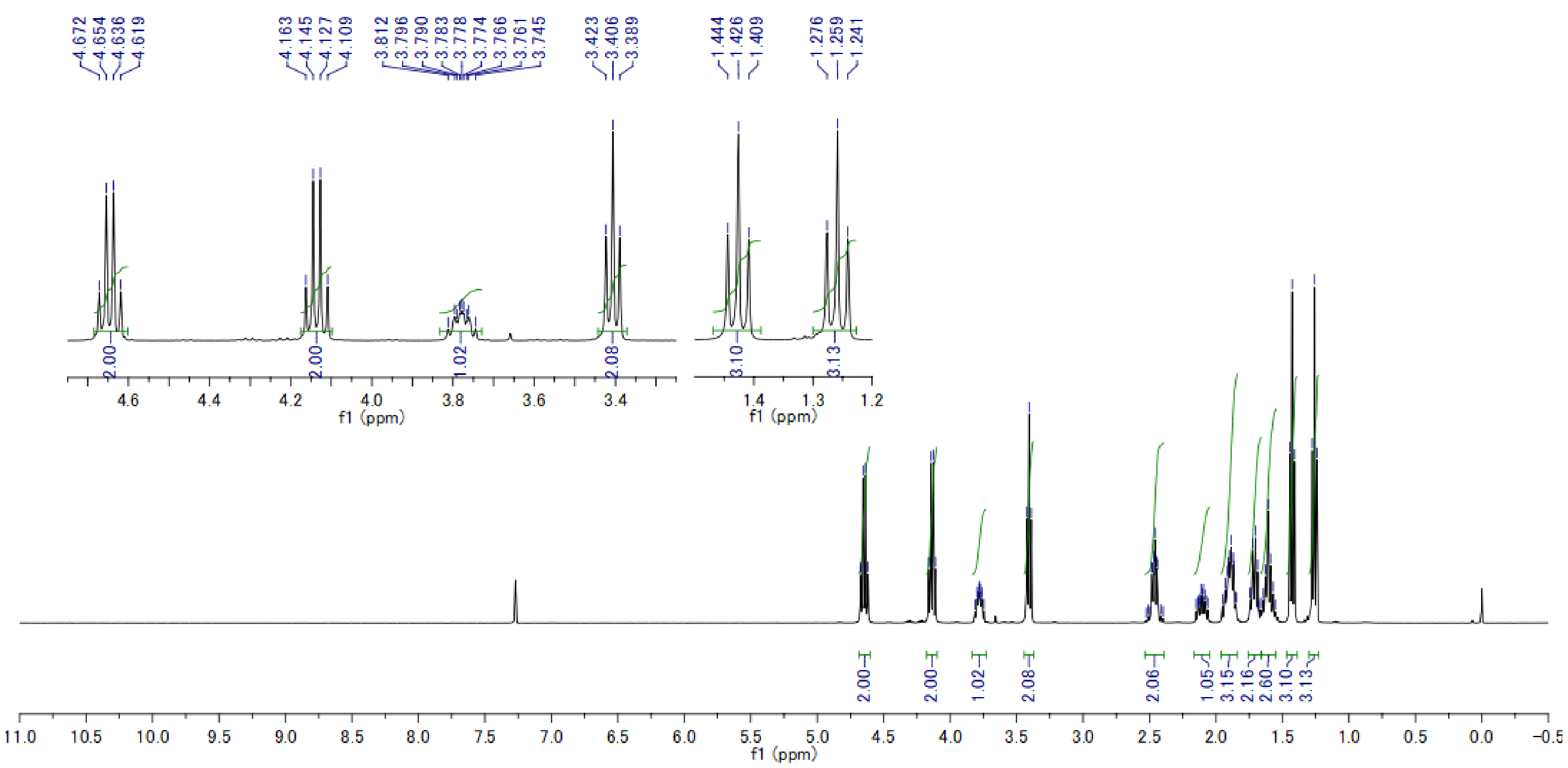

$^{13}$C NMR Spectrum of **3ai** (100 MHz, $CDCl_3$)

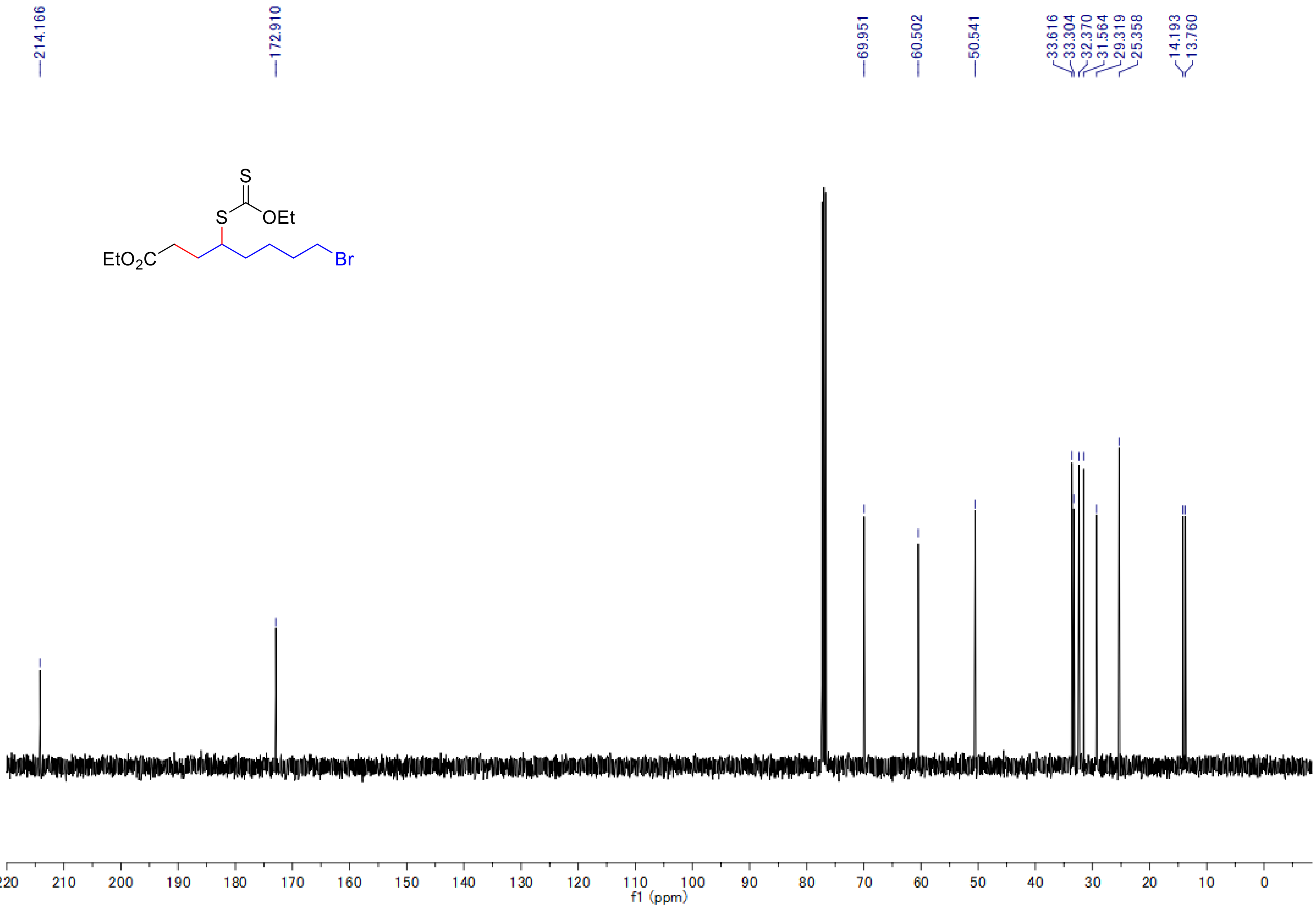

$^{1}$H NMR Spectrum of **3aj** (400 MHz, $CDCl_3$)

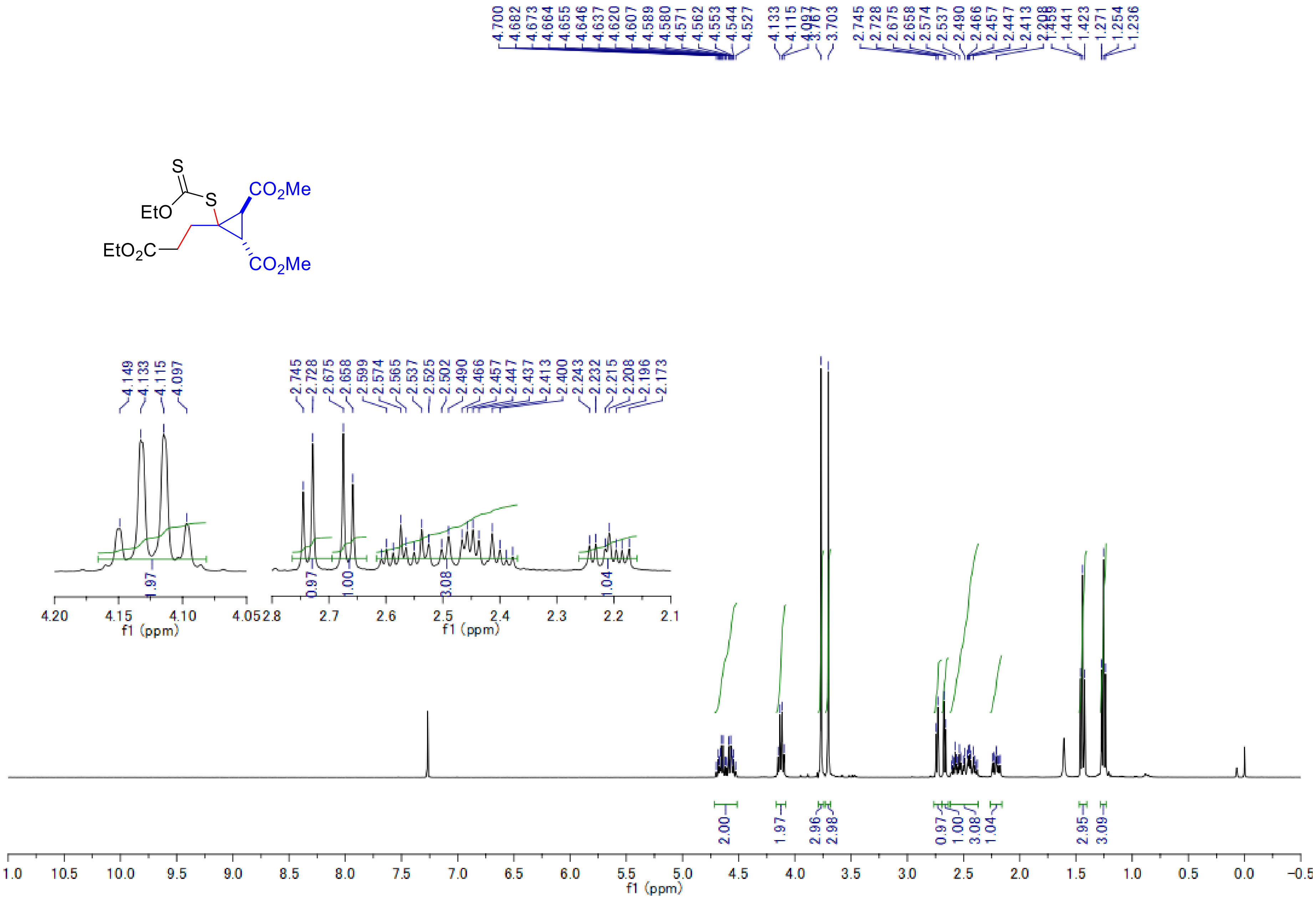

$^{13}$C NMR Spectrum of **3aj** (100 MHz, $CDCl_3$)

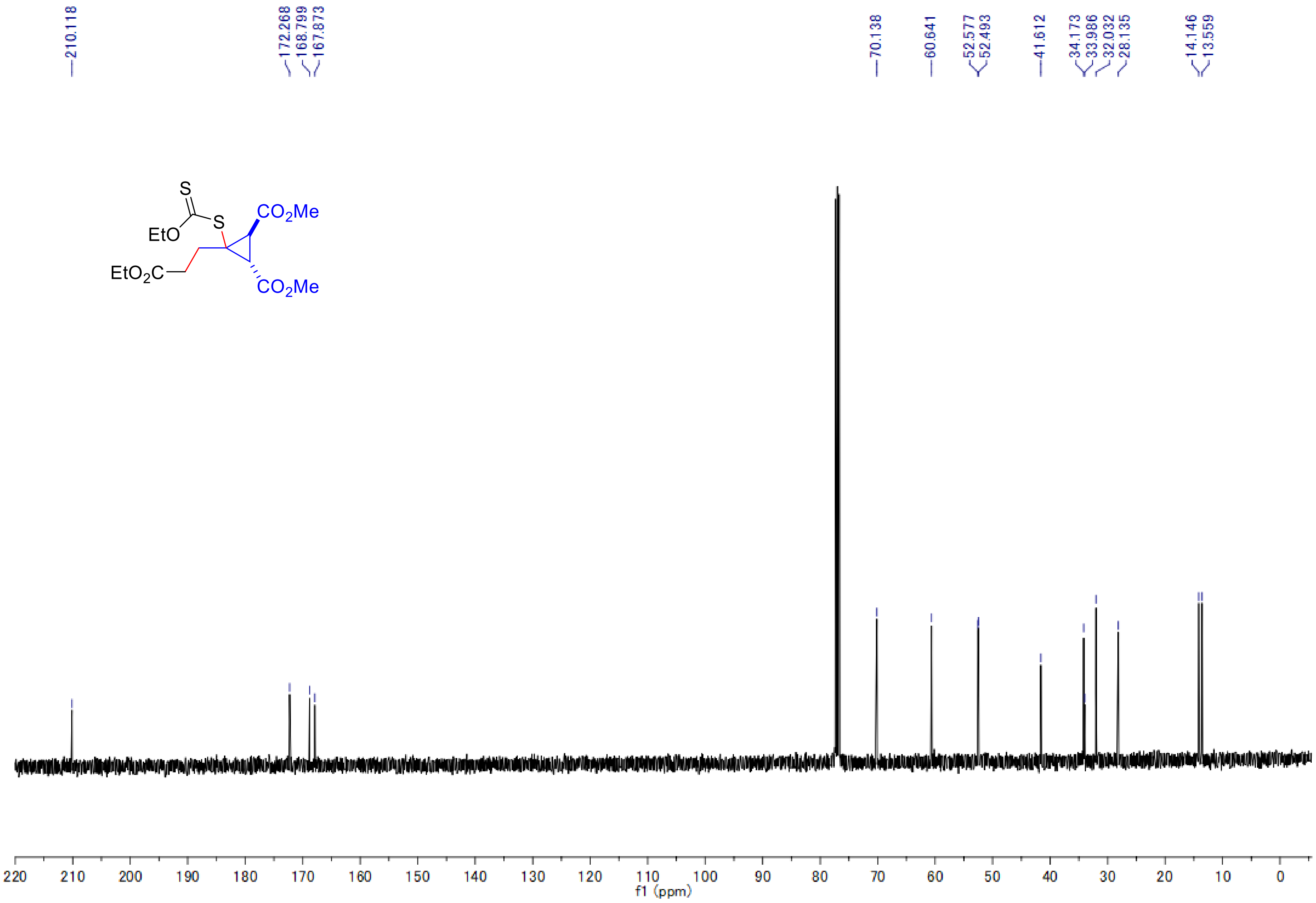

$^{1}$H NMR Spectrum of **3ak** (400 MHz, $CDCl_3$)

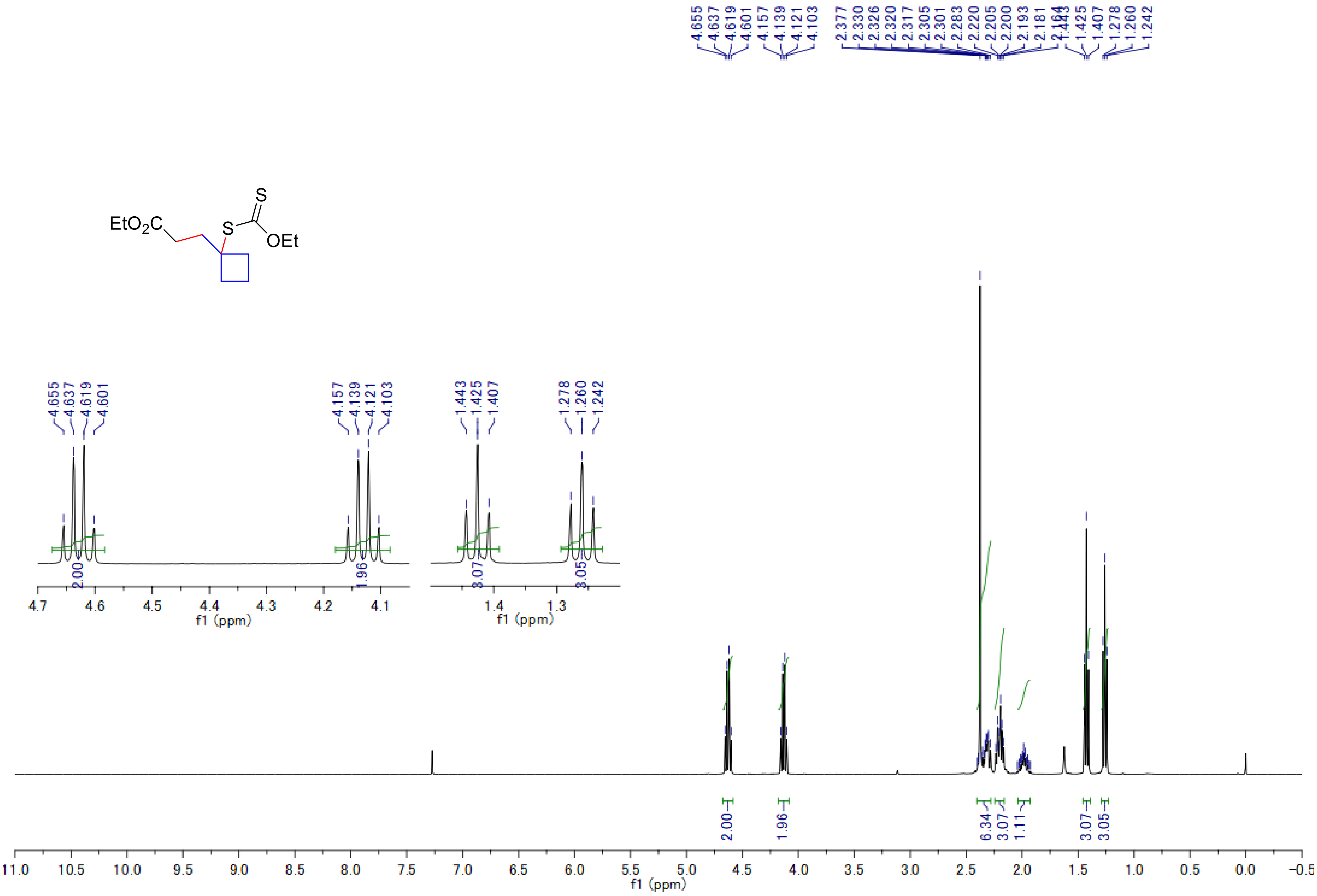

$^{13}$C NMR Spectrum of **3ak** (100 MHz, $CDCl_3$)

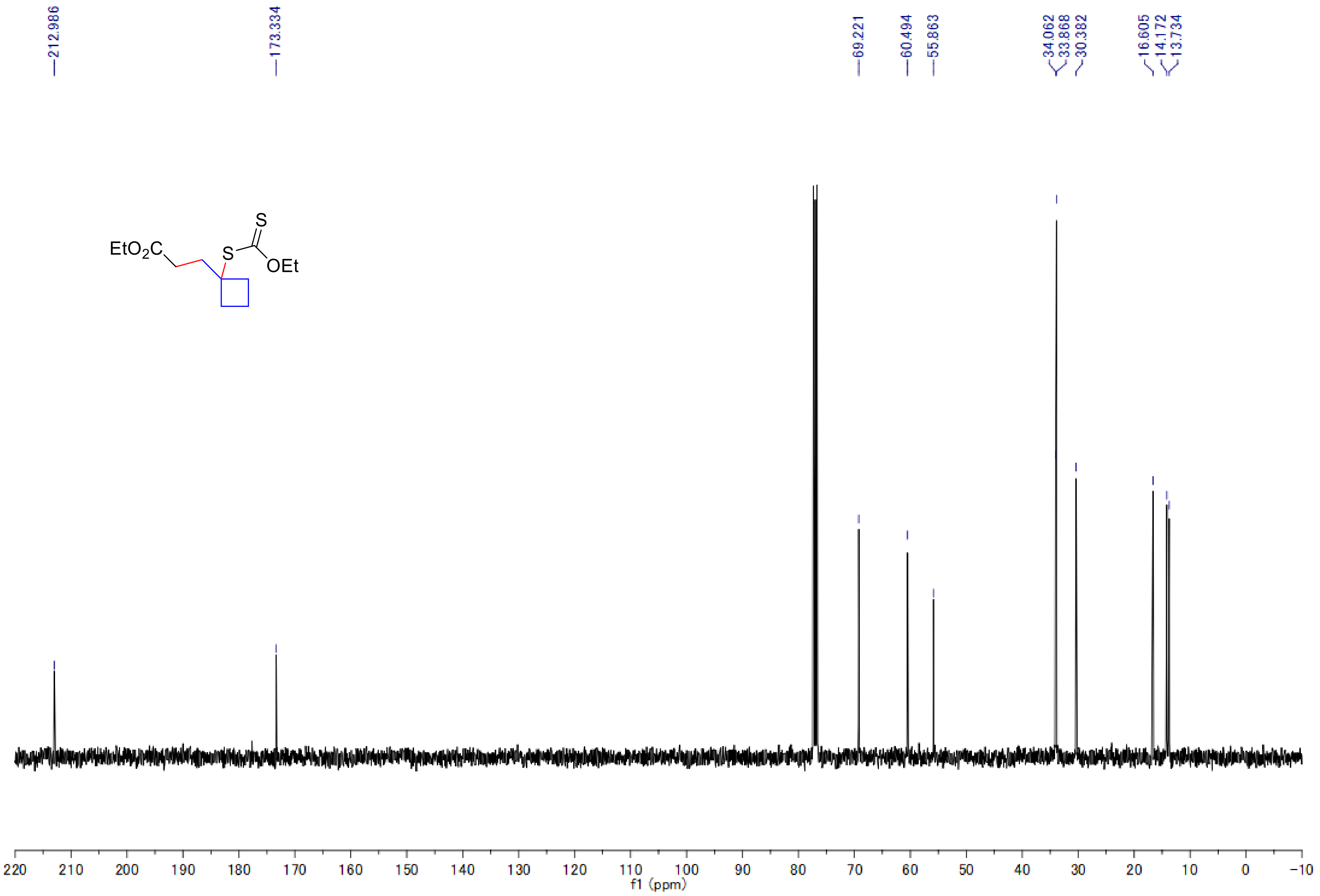

crude $^{1}$H NMR spectrum from the reaction of **1a** and **2l** (400 MHz, $CDCl_3$)

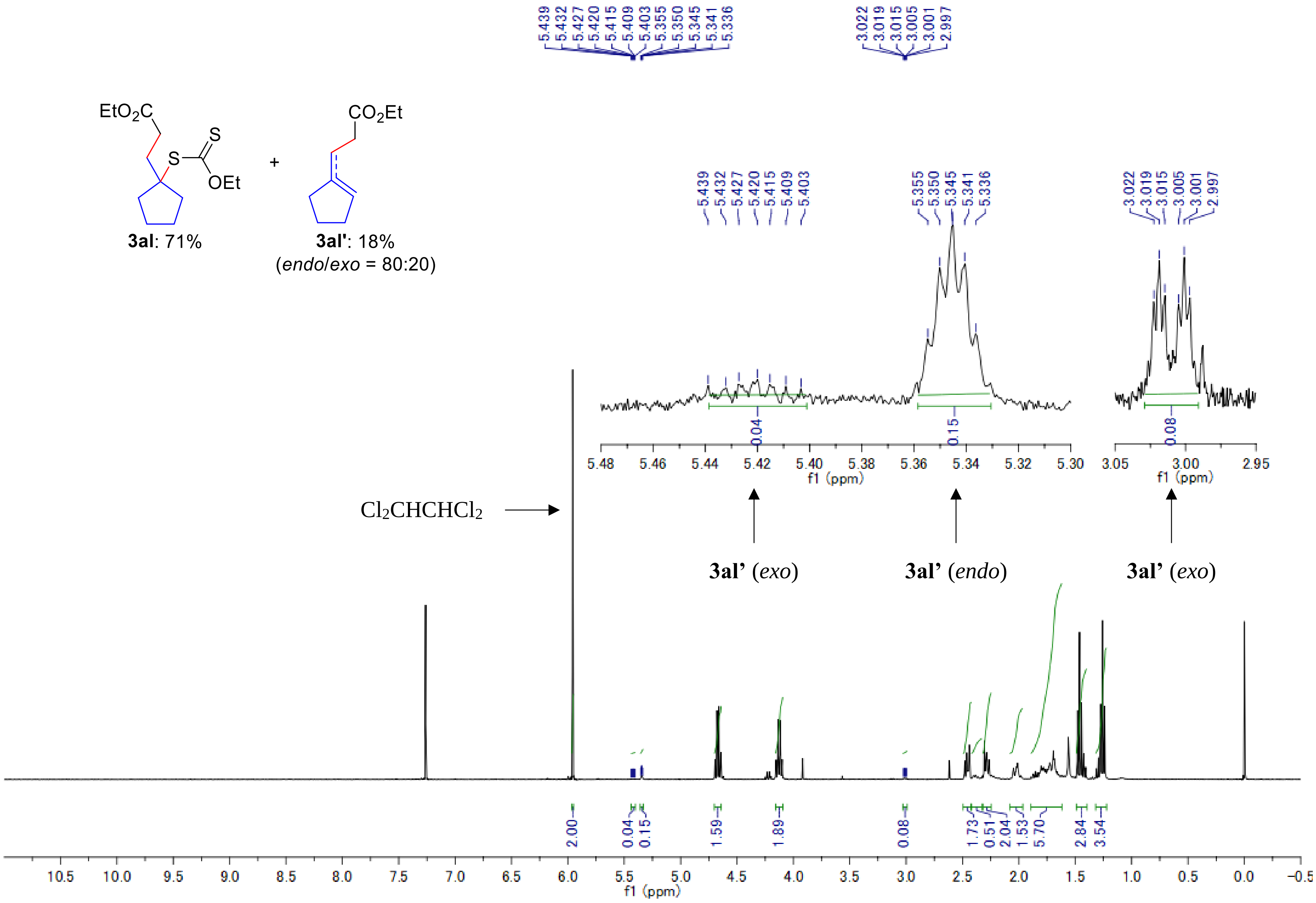

$^{1}$H NMR Spectrum of **3al** (400 MHz, $CDCl_3$)

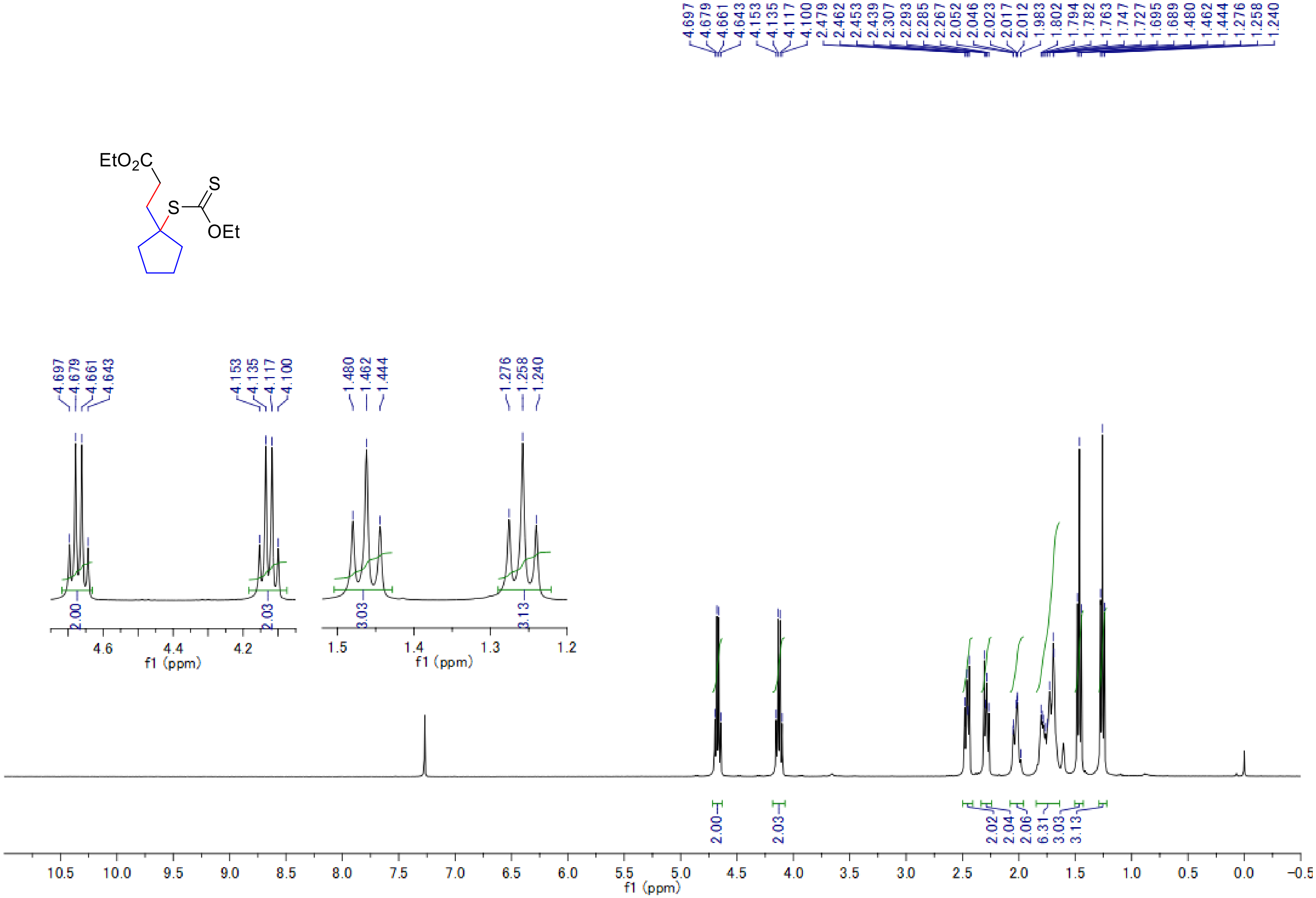

$^{13}$C NMR Spectrum of **3al** (100 MHz, $CDCl_3$)

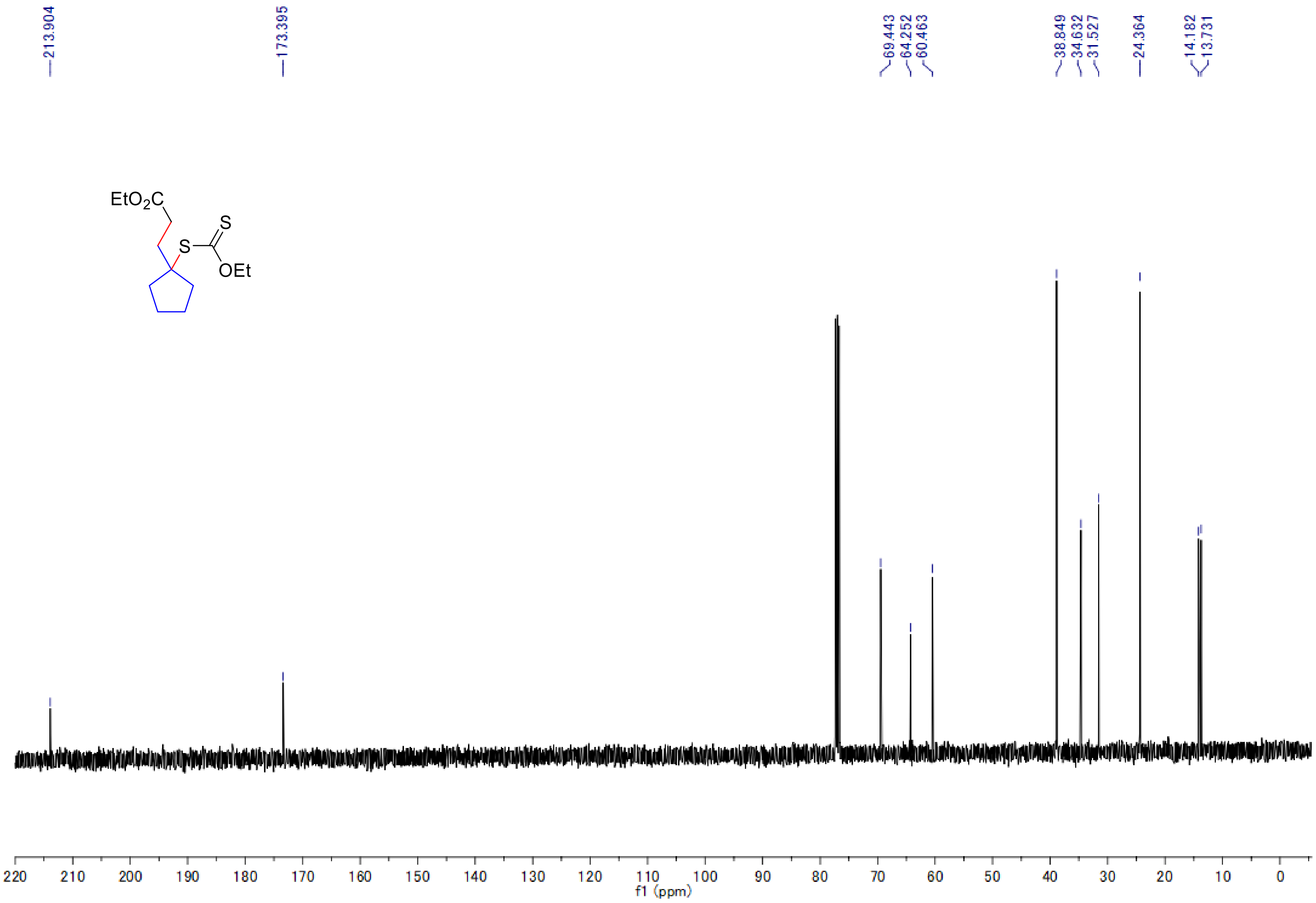

$^{1}$H NMR Spectrum of **3am** (400 MHz, $CDCl_3$)

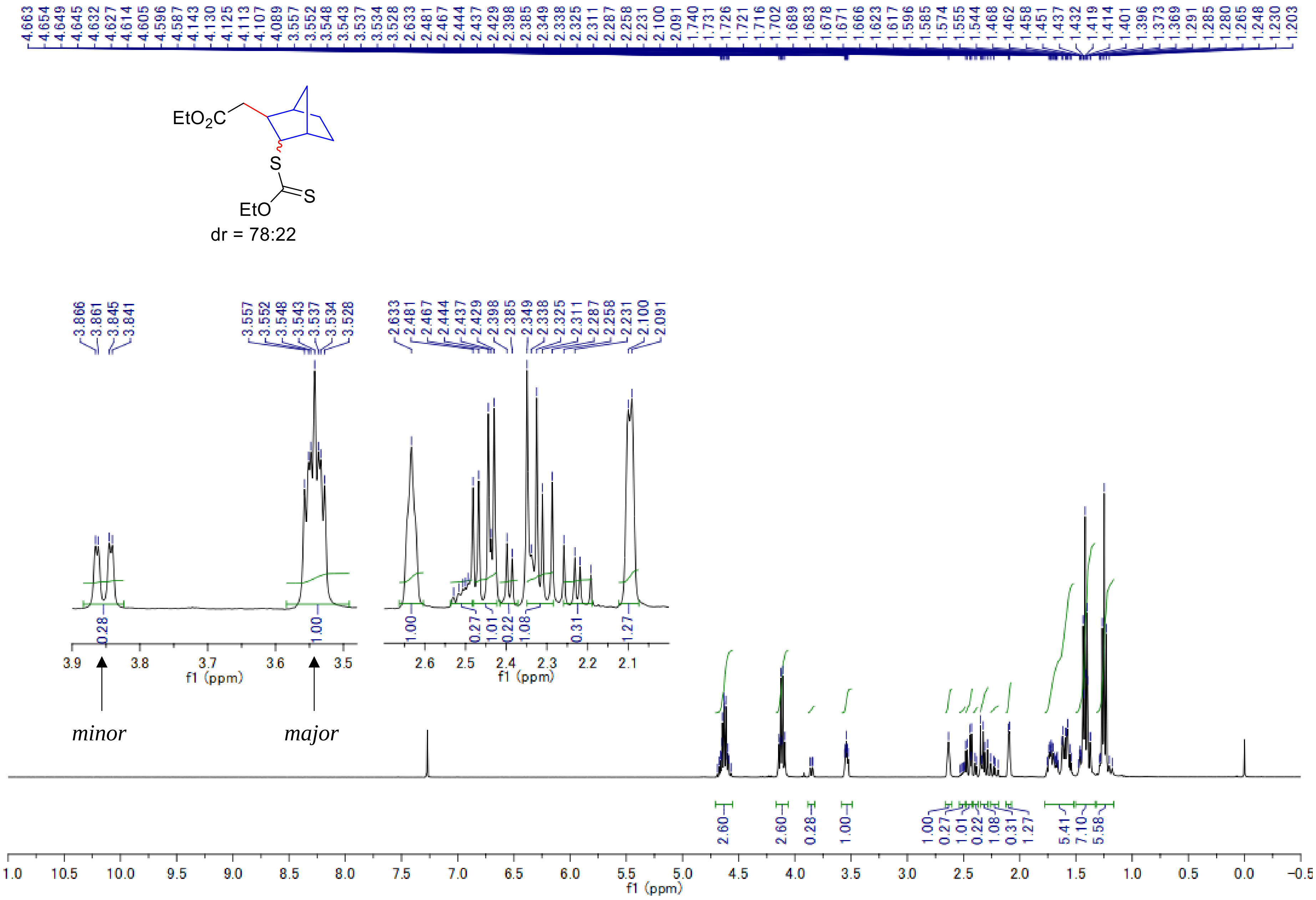

$^{13}C$ NMR Spectrum of **3am** (100 MHz, $CDCl_3$)

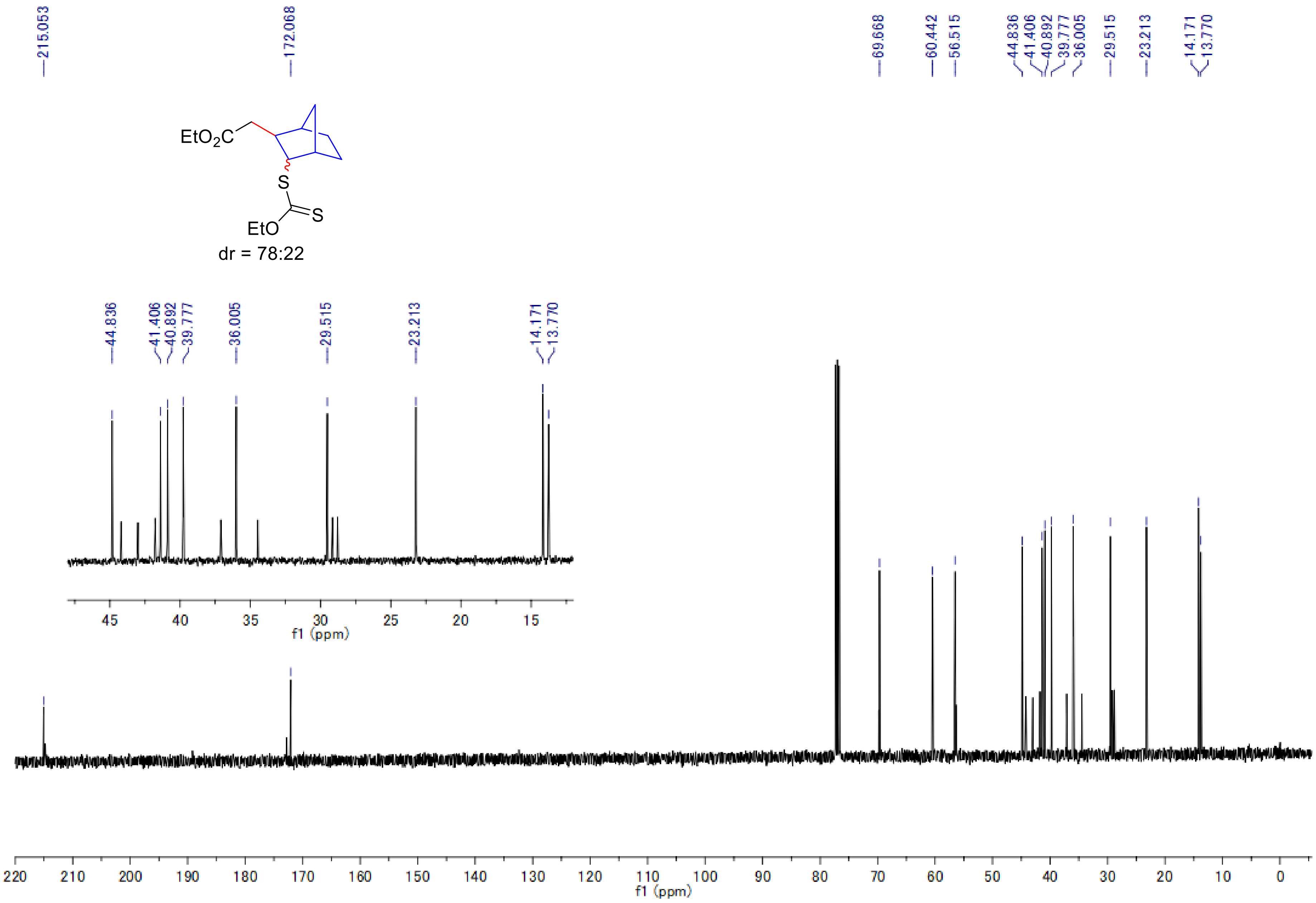

$^{1}$H NMR Spectrum of **3an** (400 MHz, $CDCl_3$)

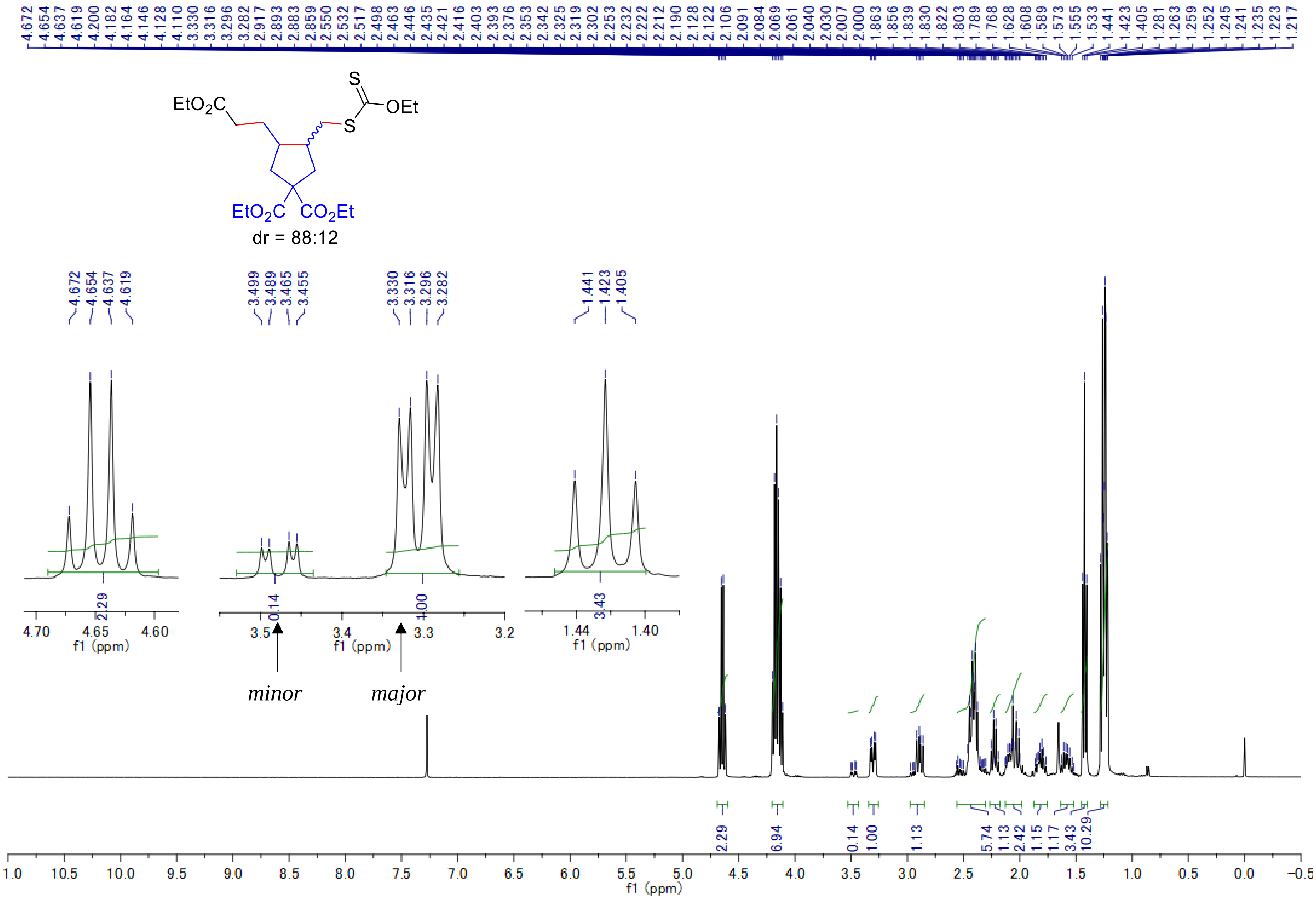

$^{13}$C NMR Spectrum of **3an** (100 MHz, $CDCl_3$)

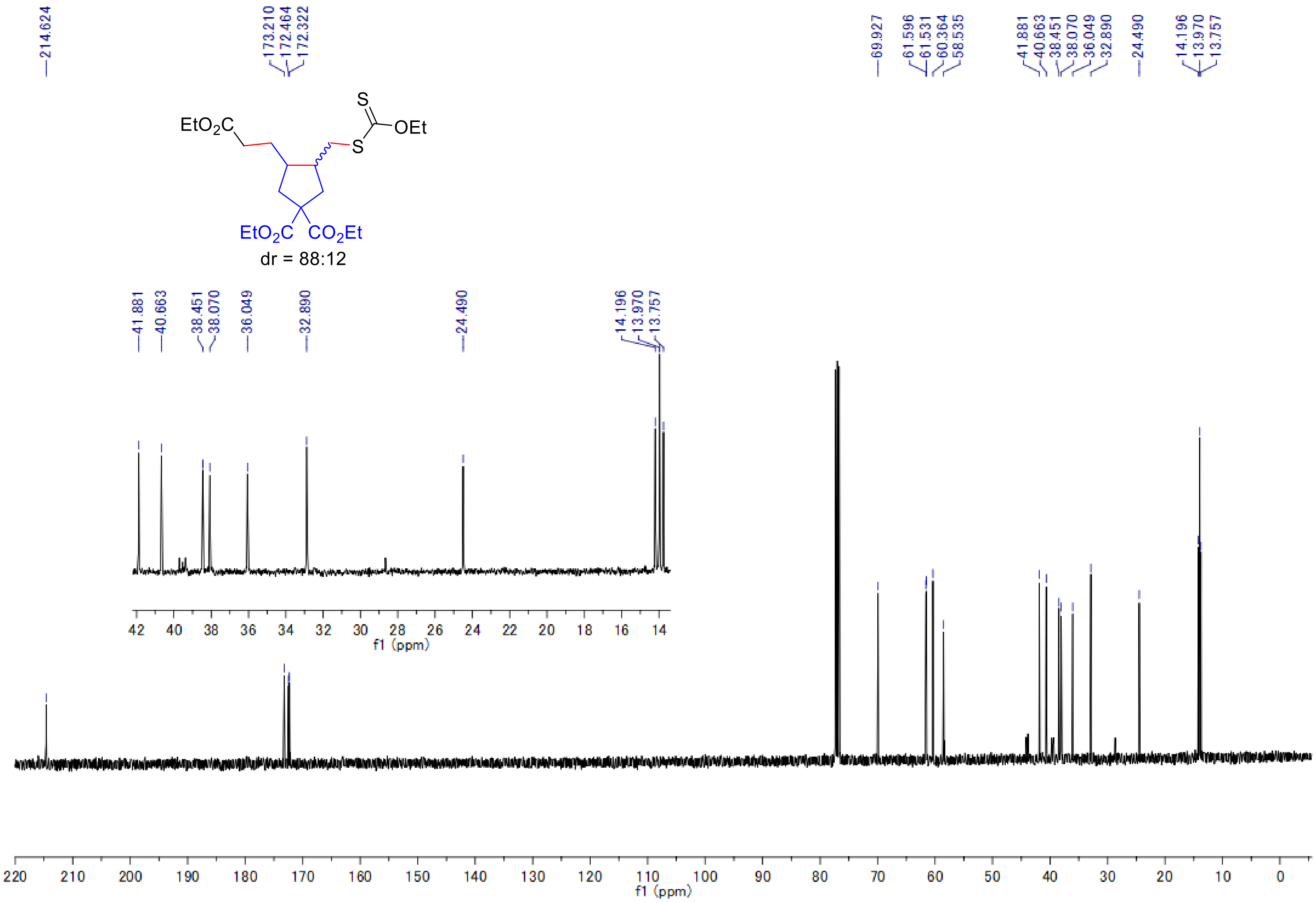

$^{1}$H NMR Spectrum of **3ao** (400 MHz, $CDCl_3$)

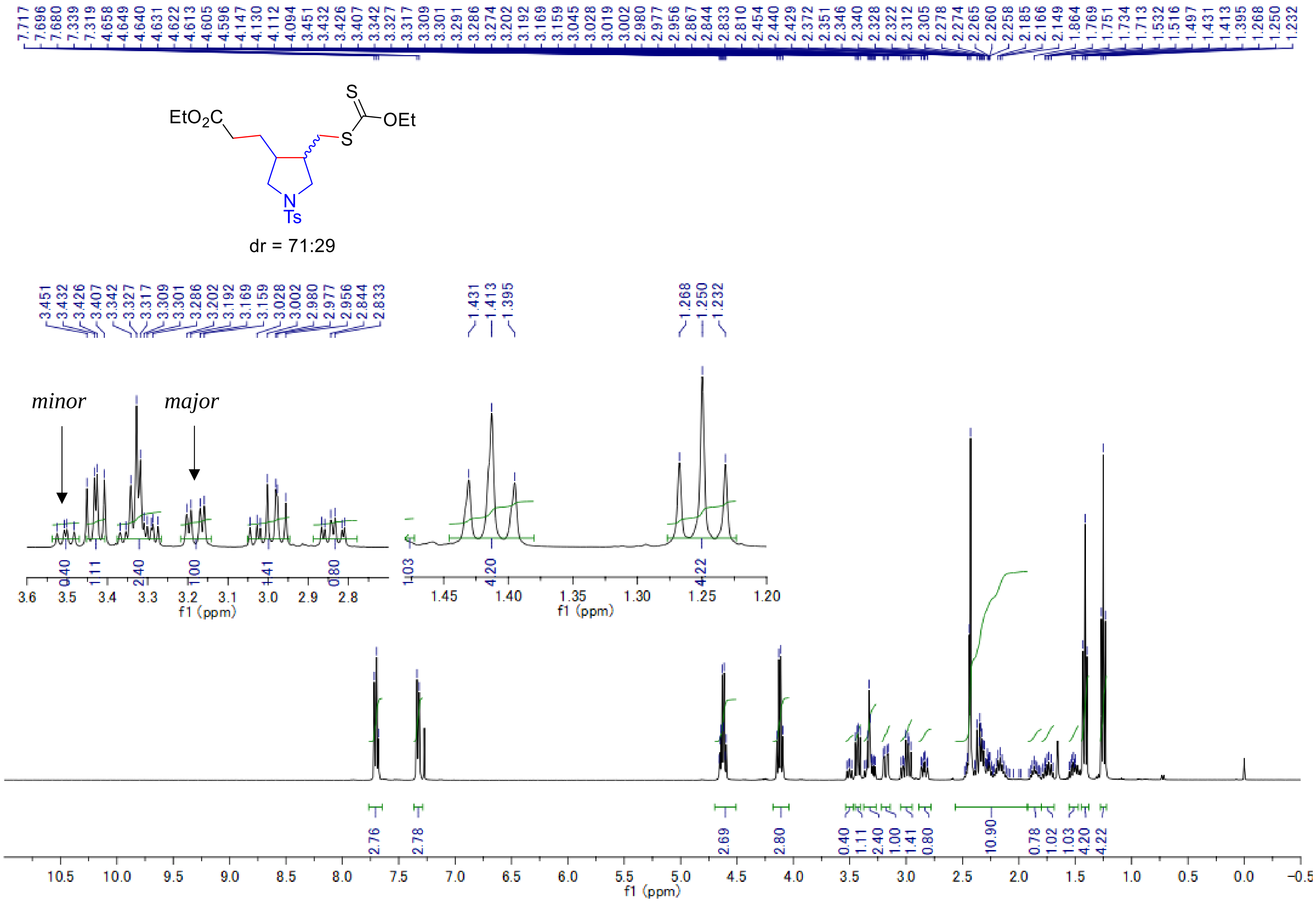

$^{13}$C NMR Spectrum of **3ao** (100 MHz, $CDCl_3$)

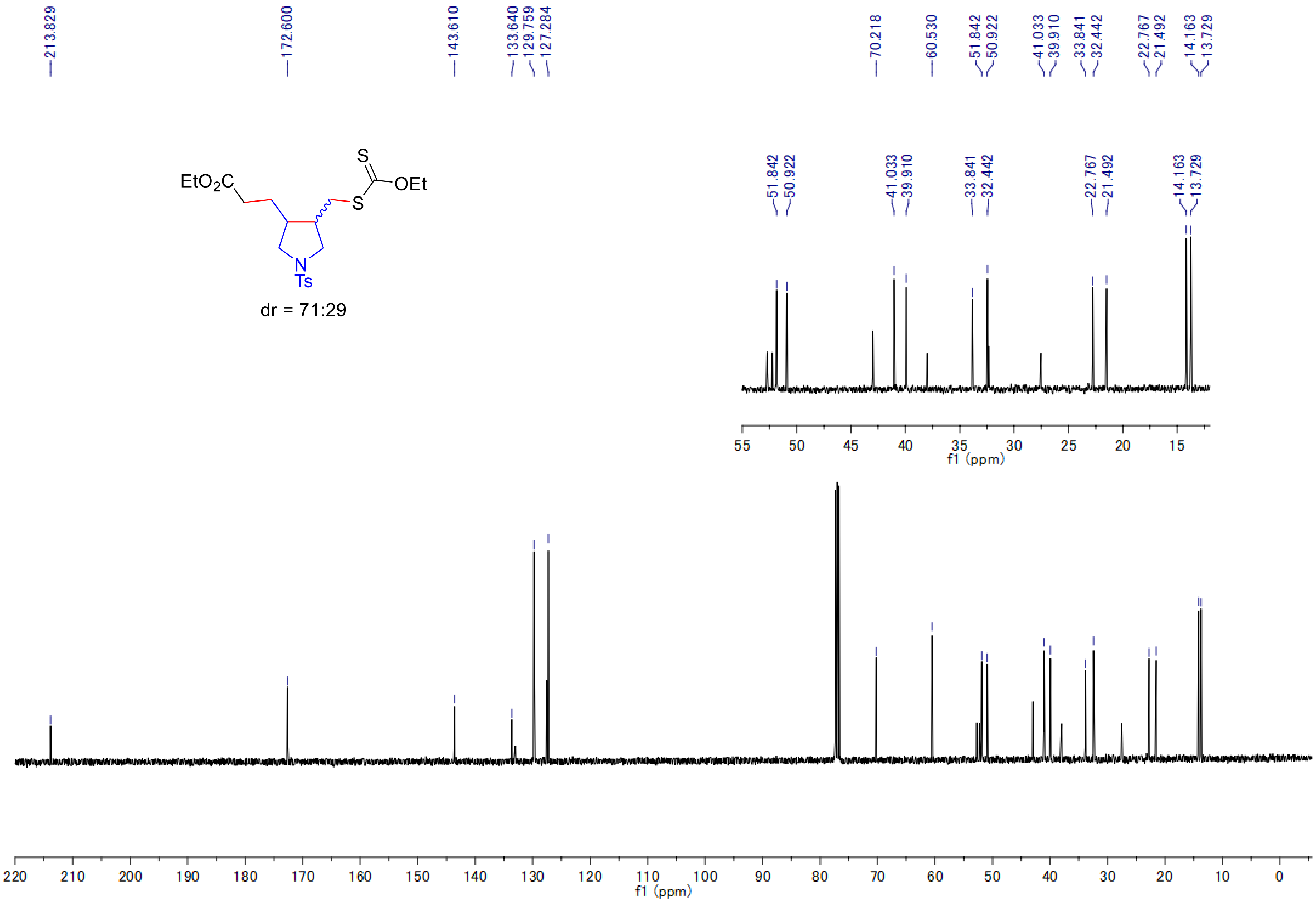

$^{1}$H NMR Spectrum of **3ap** (400 MHz, $C_6D_6$)

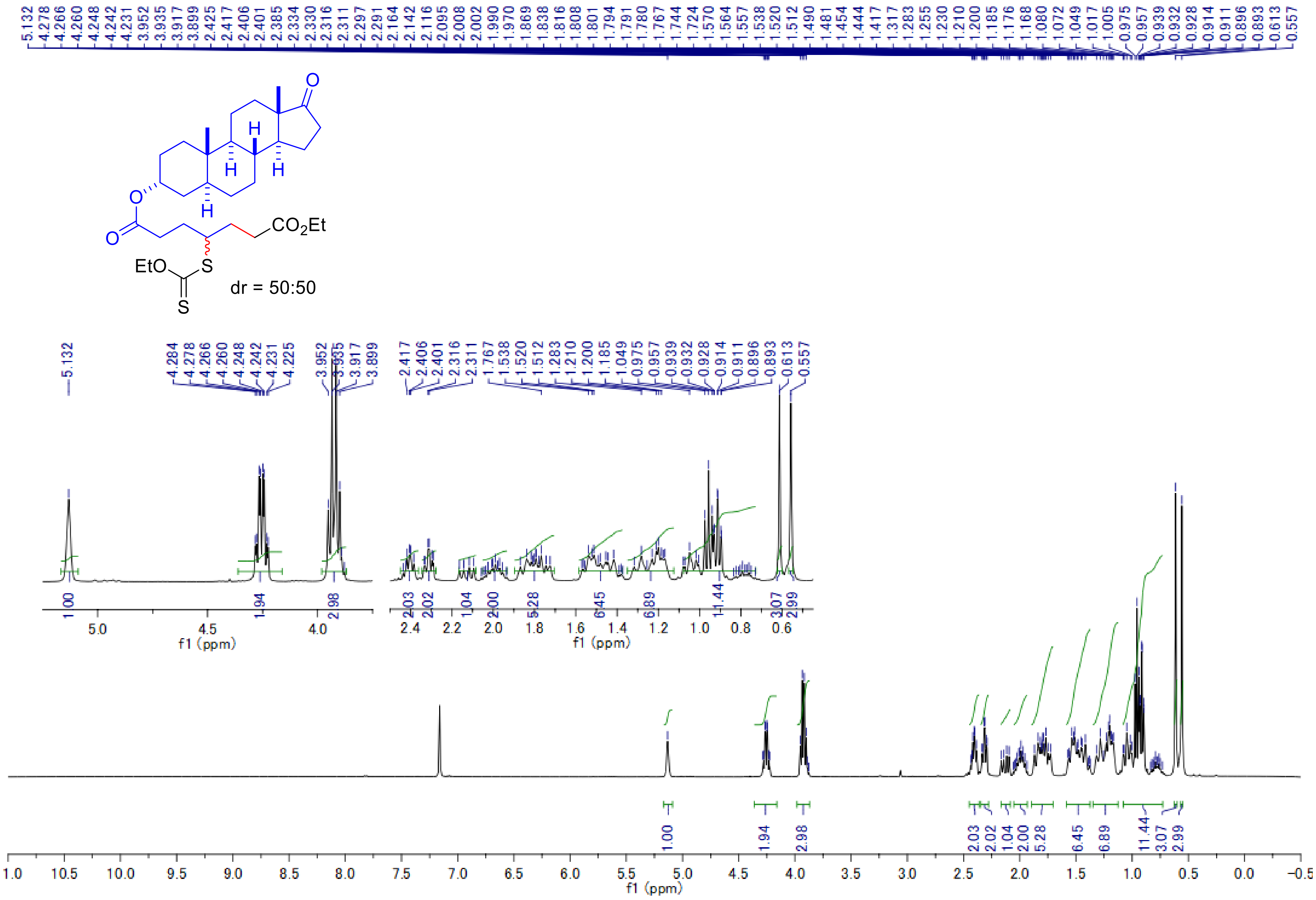

$^{13}$C NMR Spectrum of **3ap** (100 MHz, $C_6D_6$)

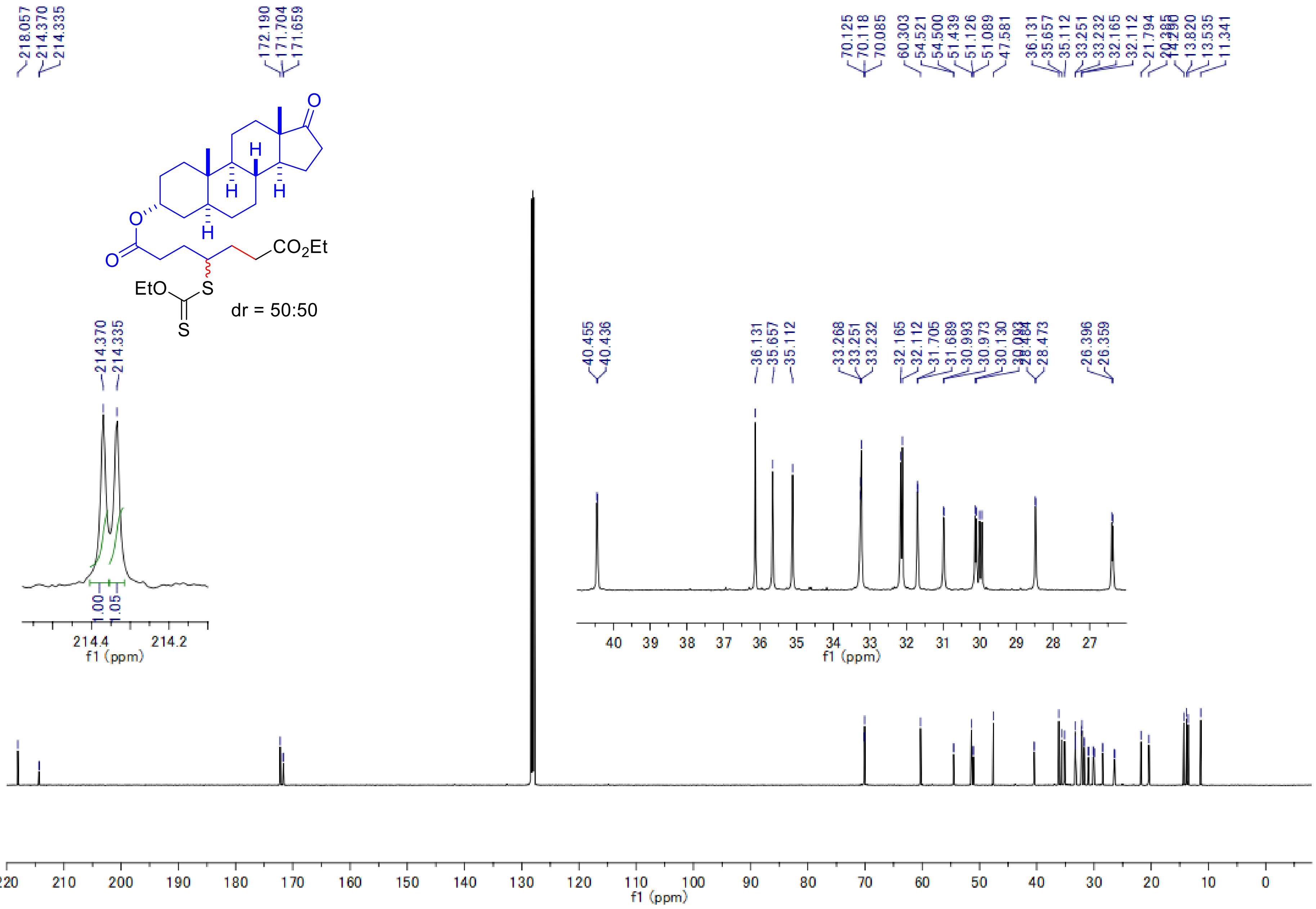

$^{1}$H NMR Spectrum of **3aq** (400 MHz, $CDCl_3$)

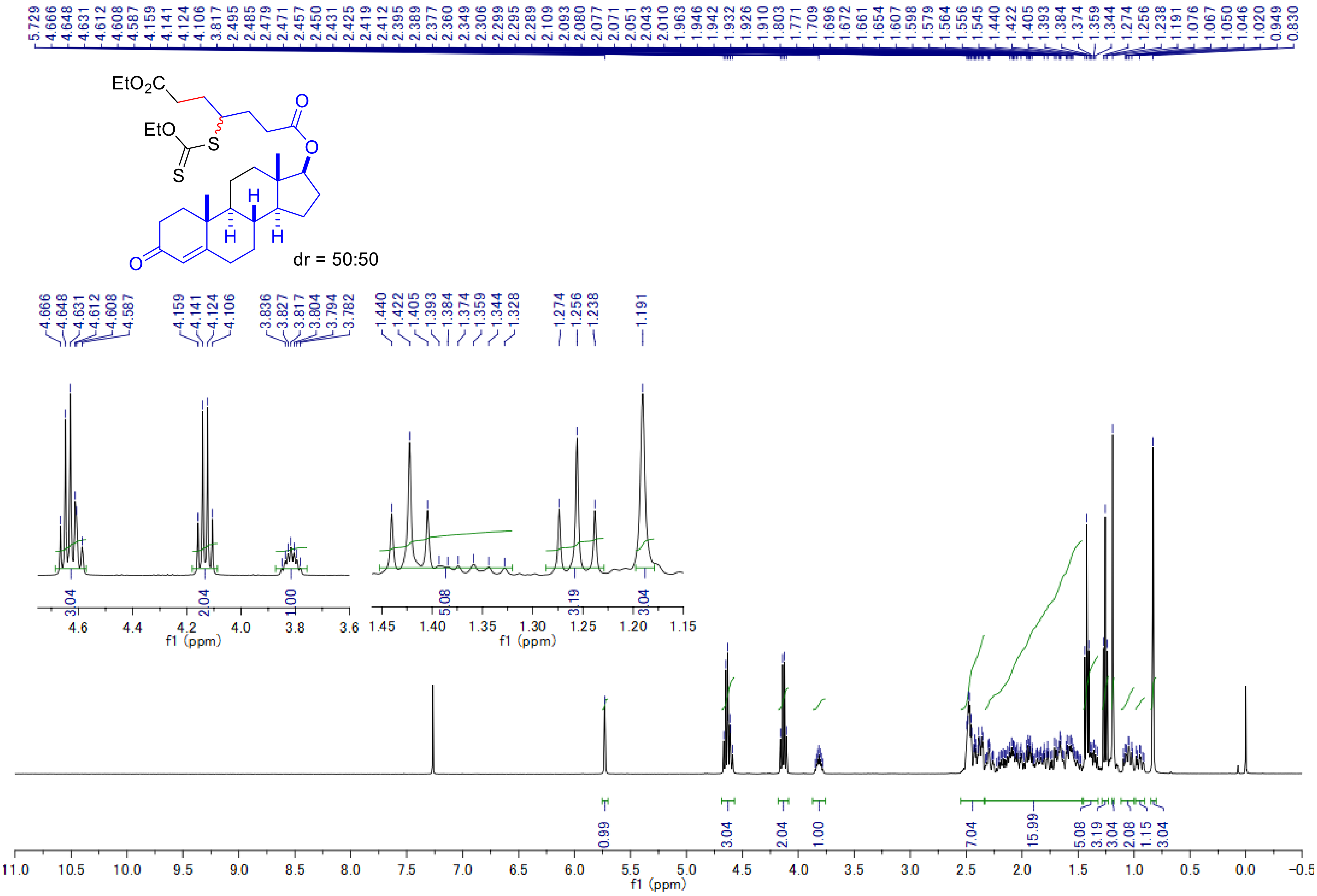

$^{13}$C NMR Spectrum of **3aq** (100 MHz, $CDCl_3$)

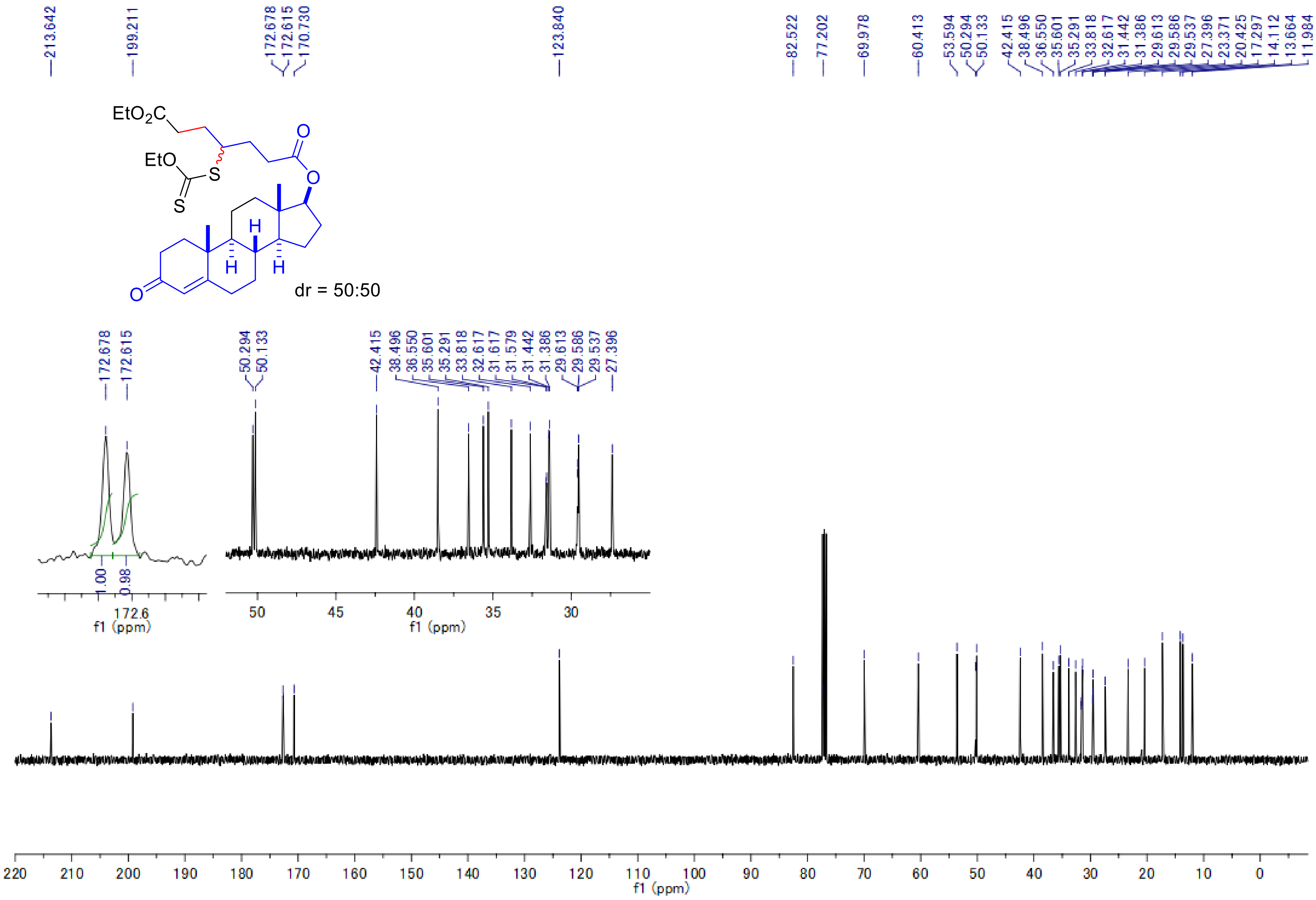

$^{1}$H NMR Spectrum of **3bb** (500 MHz, $CDCl_3$)

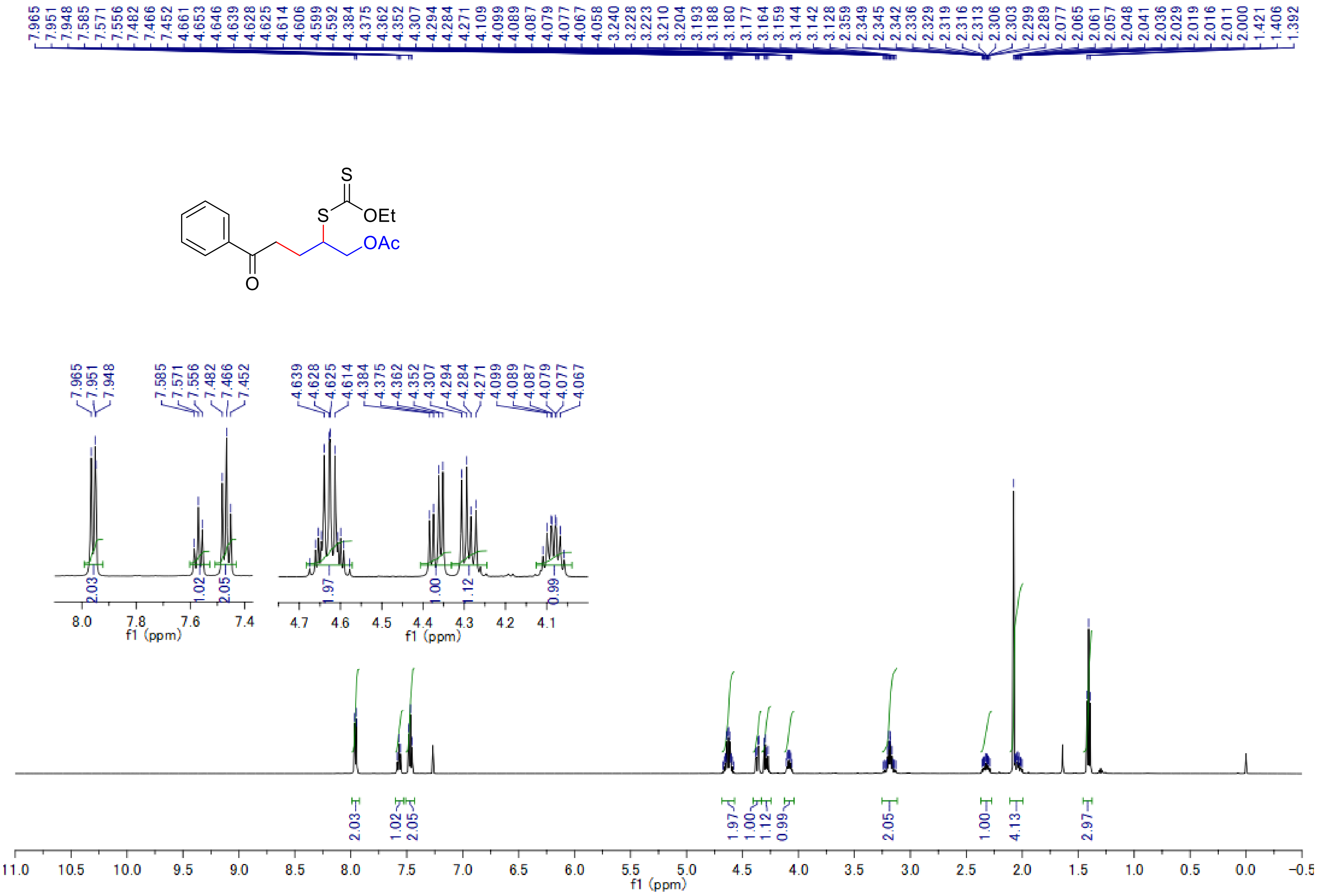

$^{13}$C NMR Spectrum of **3bb** (125 MHz, $CDCl_3$)

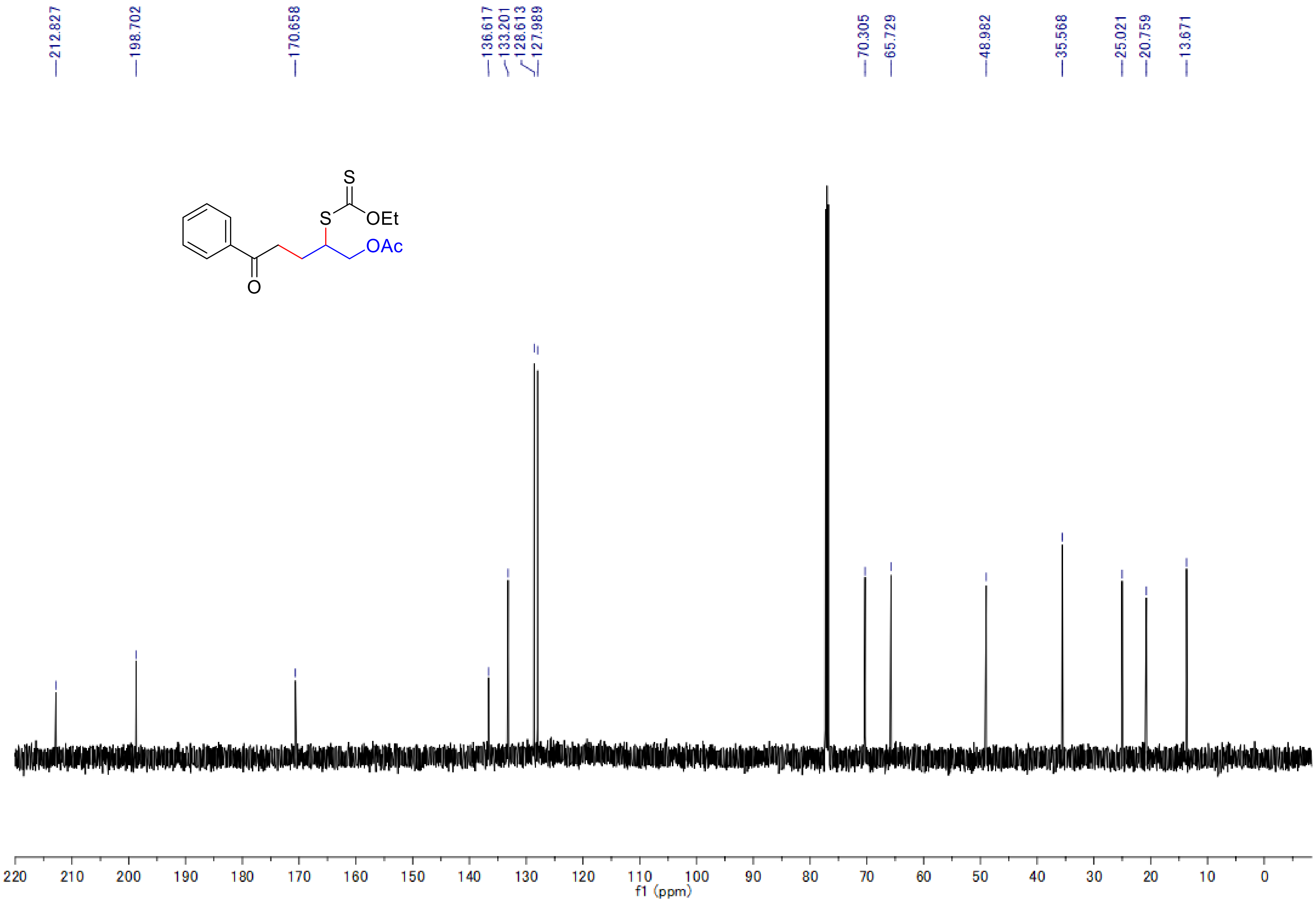

$^{1}$H NMR Spectrum of **3cb** (400 MHz, $CDCl_3$)

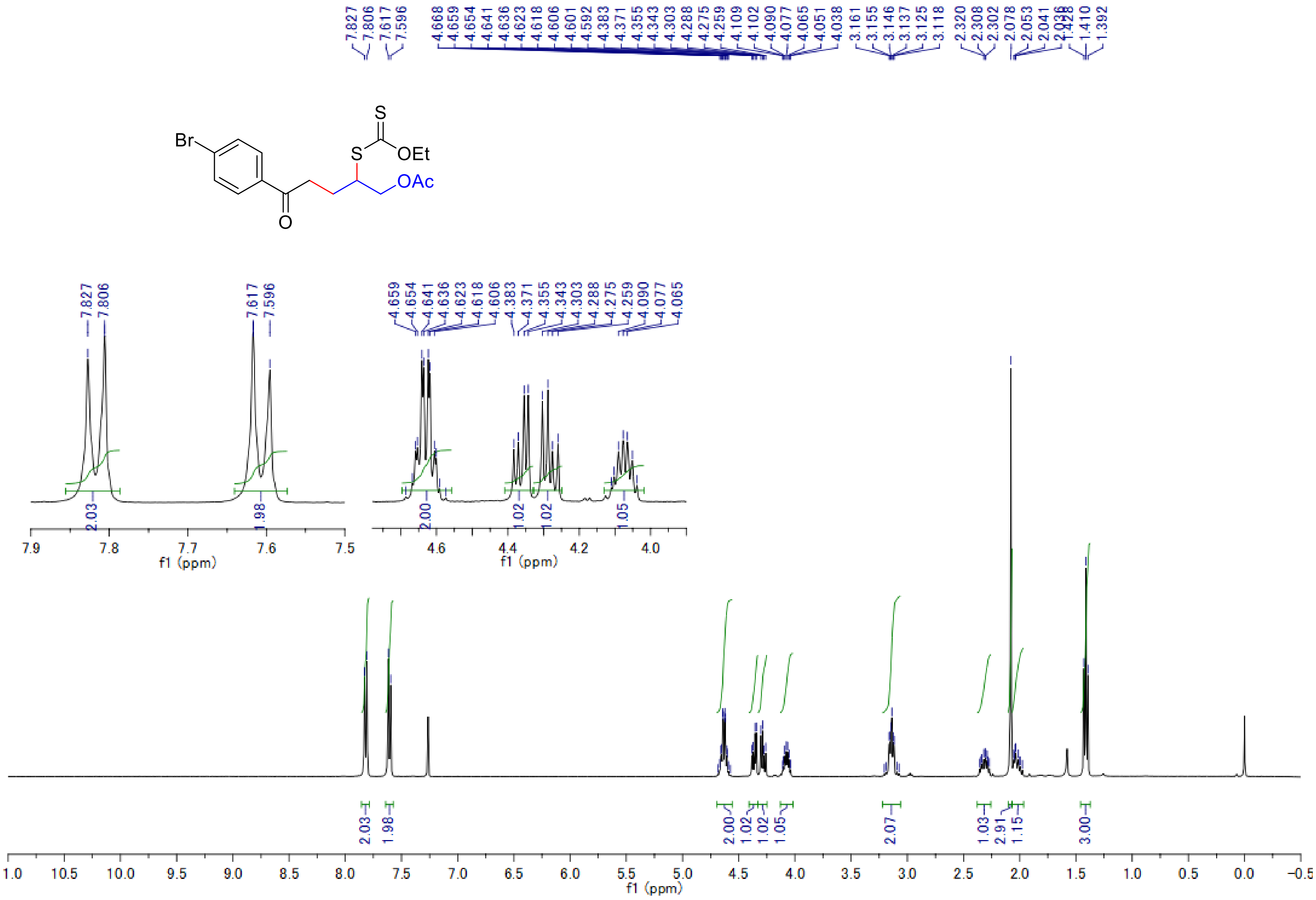

$^{13}$C NMR Spectrum of **3cb** (100 MHz, $CDCl_3$)

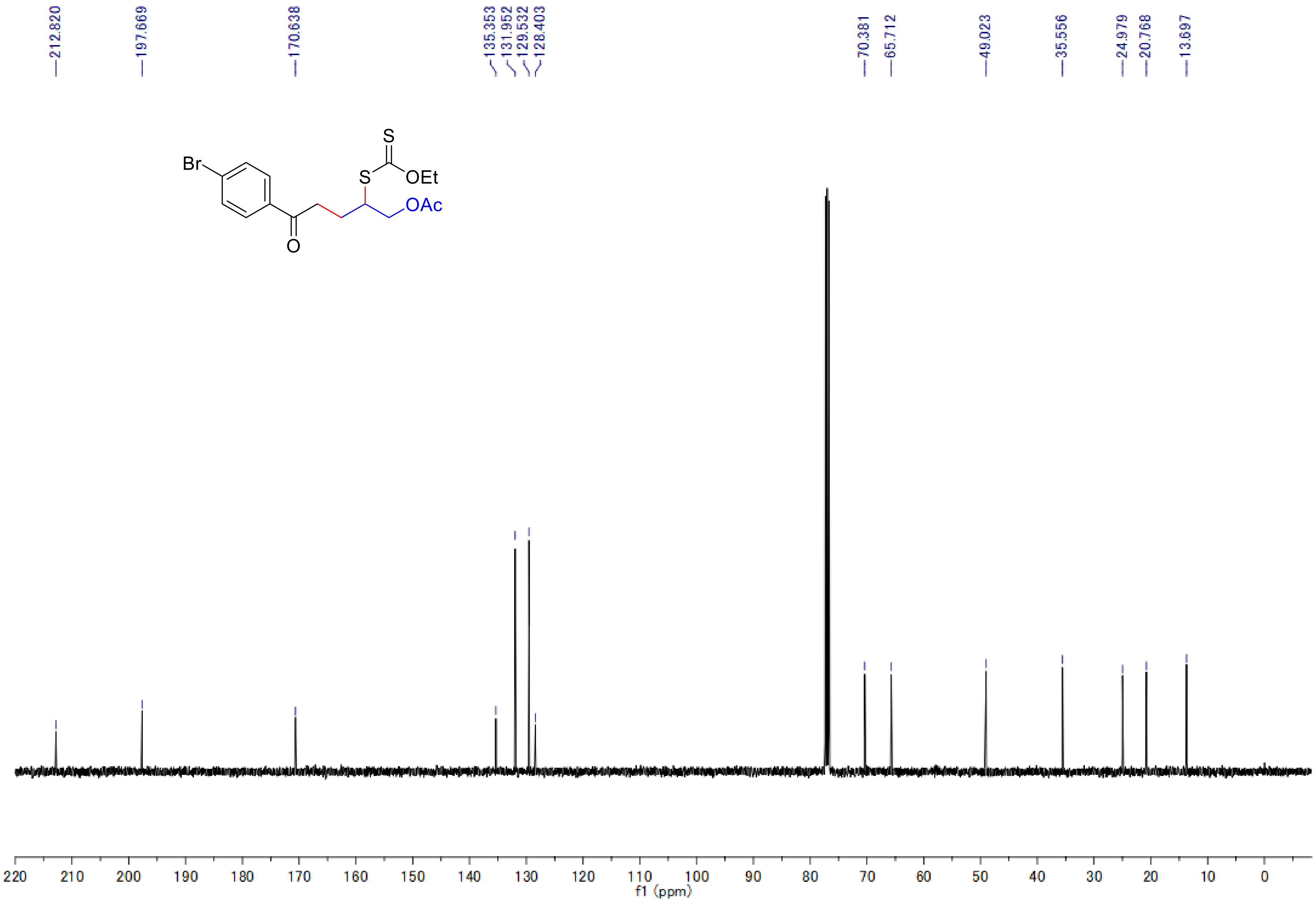

$^{1}$H NMR Spectrum of **3db** (500 MHz, $CDCl_3$)

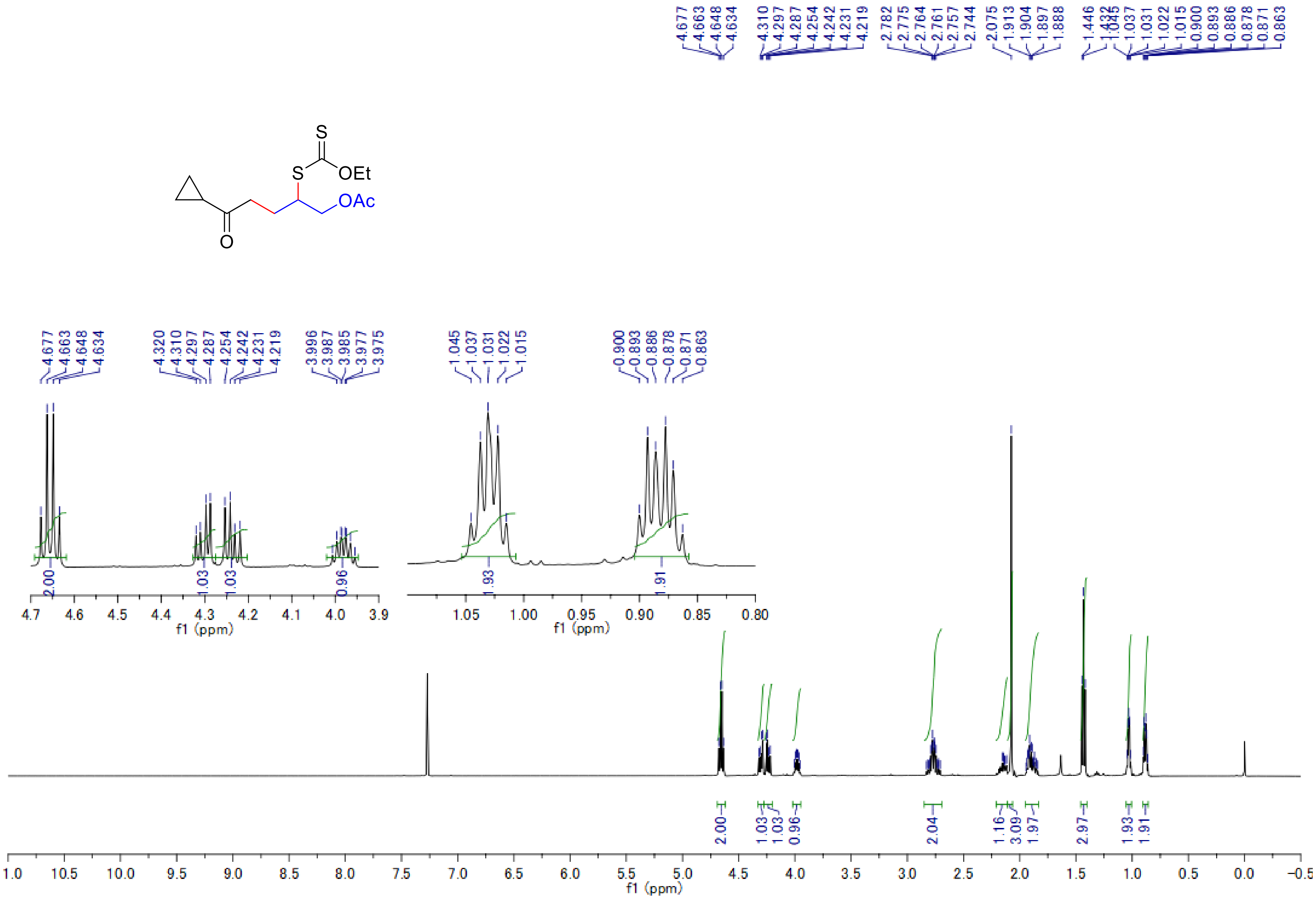

$^{13}$C NMR Spectrum of **3db** (125 MHz, $CDCl_3$)

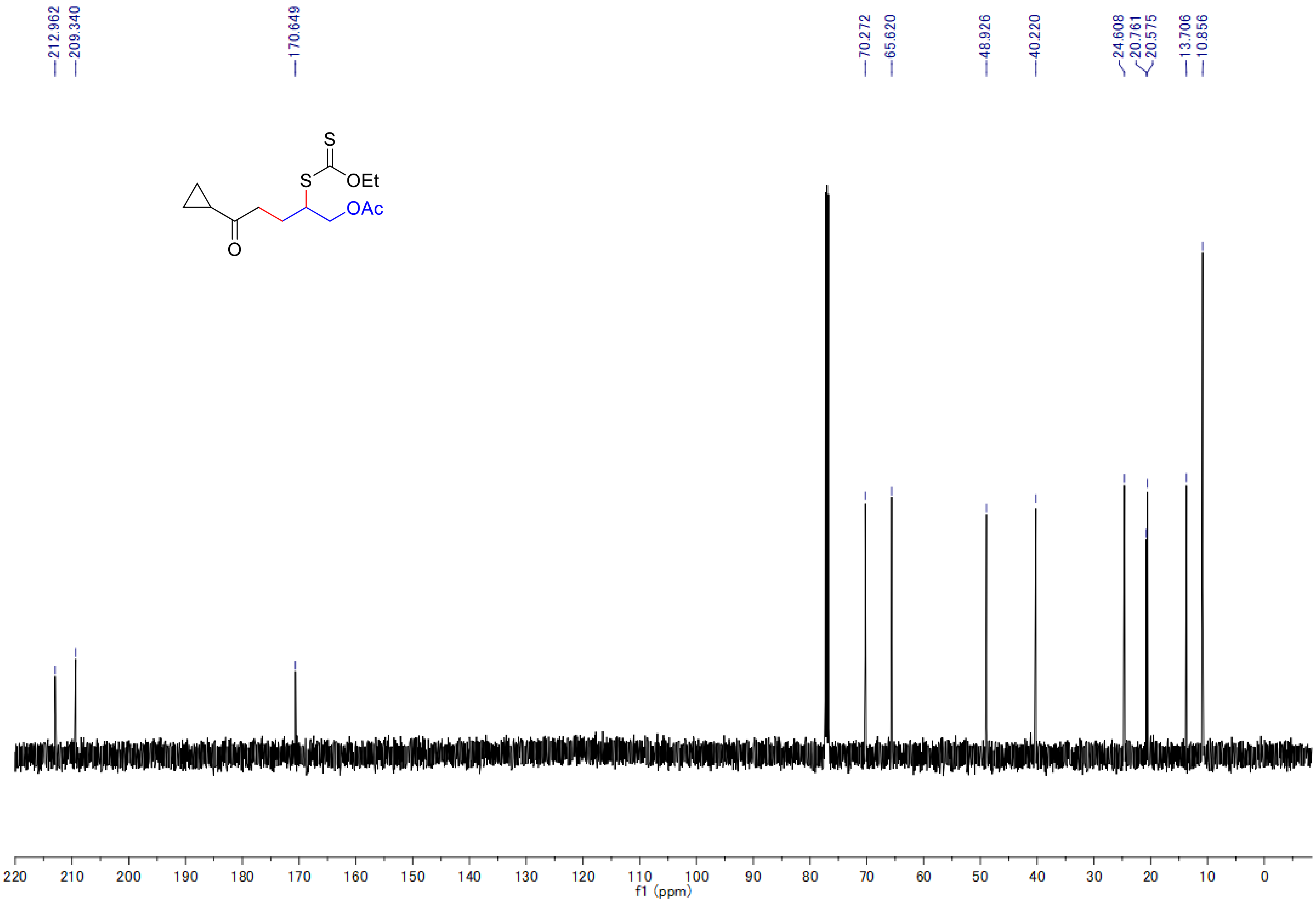

$^{1}$H NMR Spectrum of **3eb** (500 MHz, $CDCl_3$)

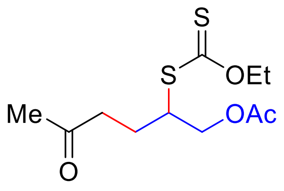


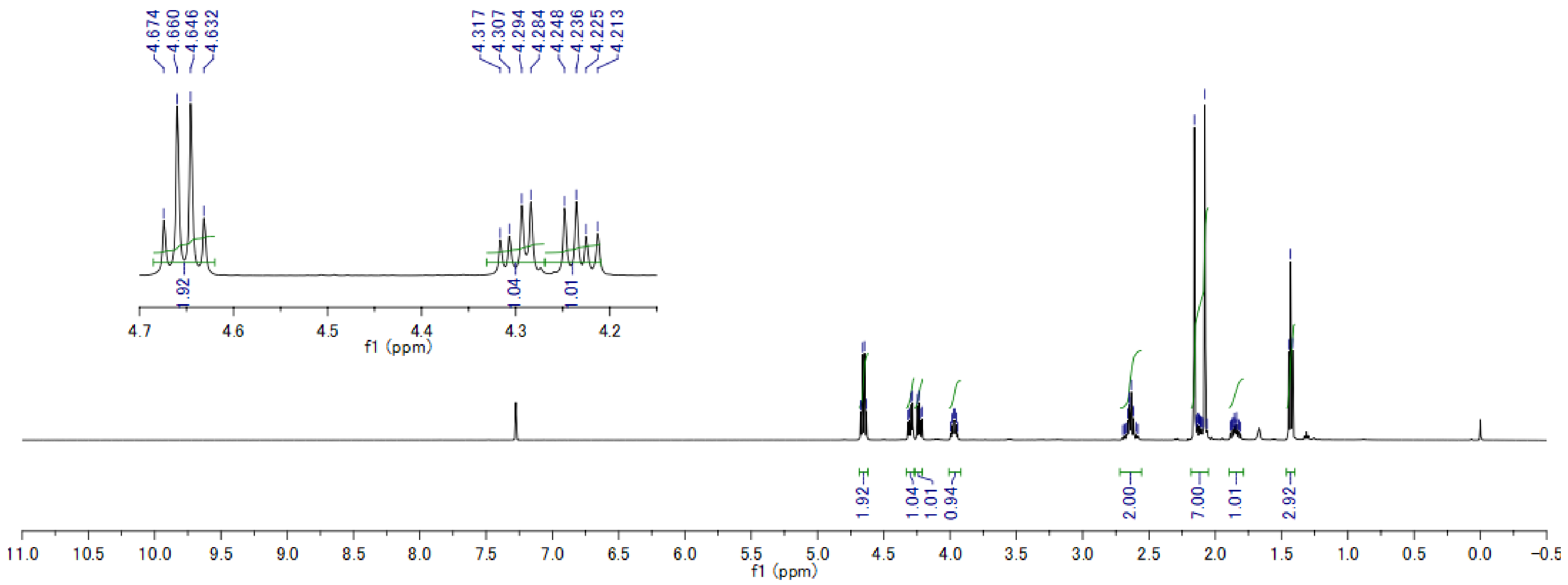

$^{13}$C NMR Spectrum of **3eb** (125 MHz, $CDCl_3$)

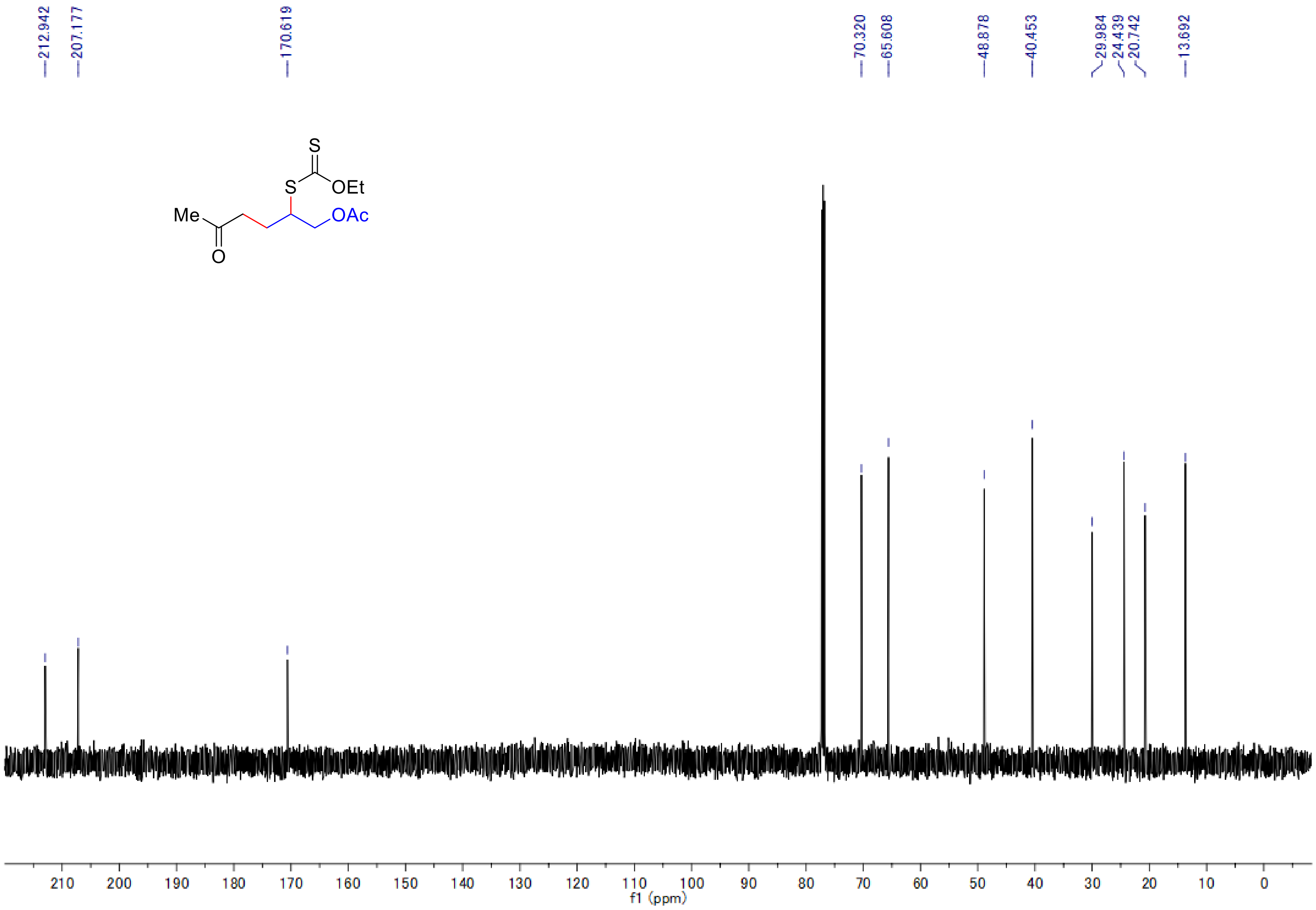

$^{1}$H NMR Spectrum of **3fb** (400 MHz, $CDCl_3$)

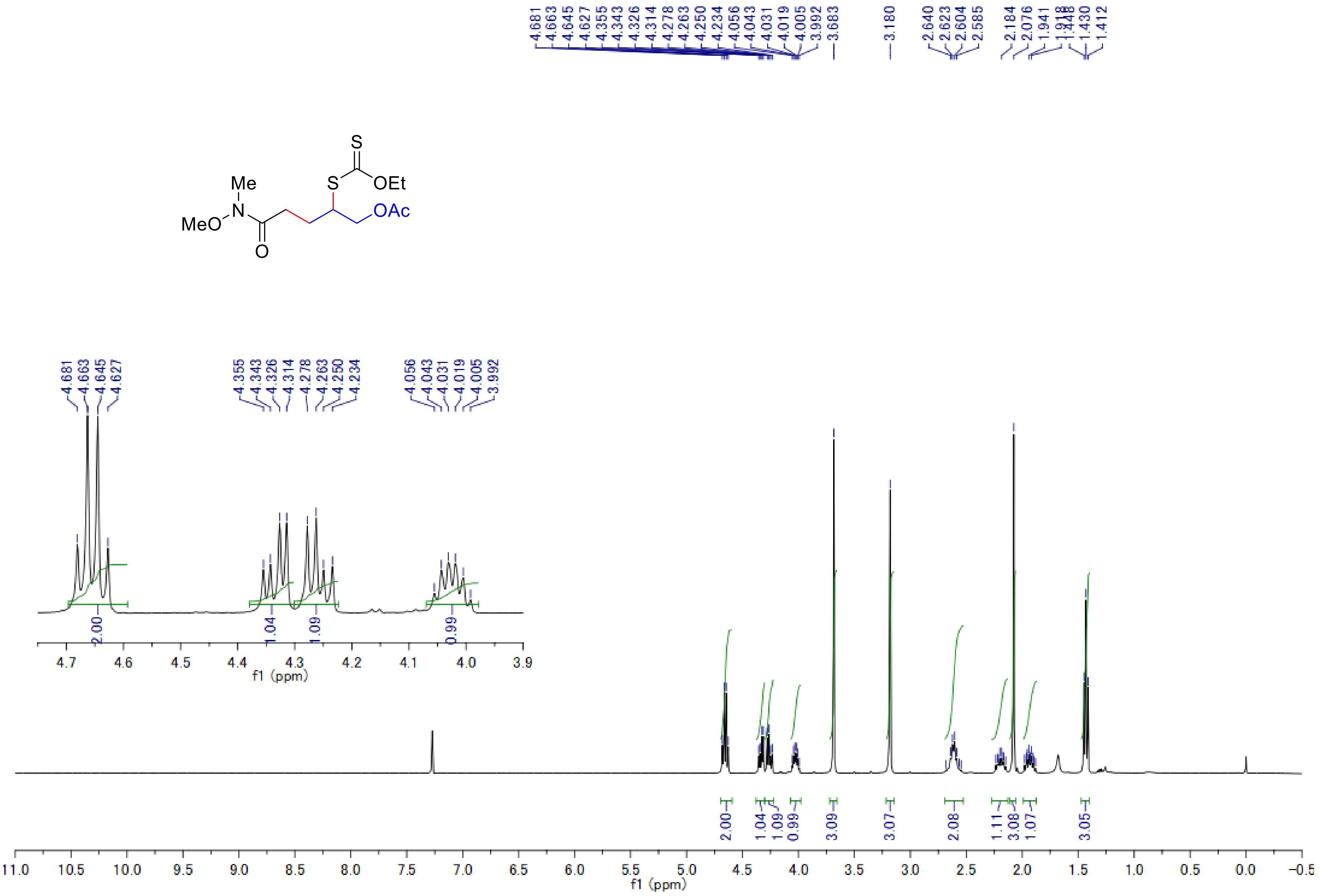

$^{13}$C NMR Spectrum of **3fb** (100 MHz, $CDCl_3$)

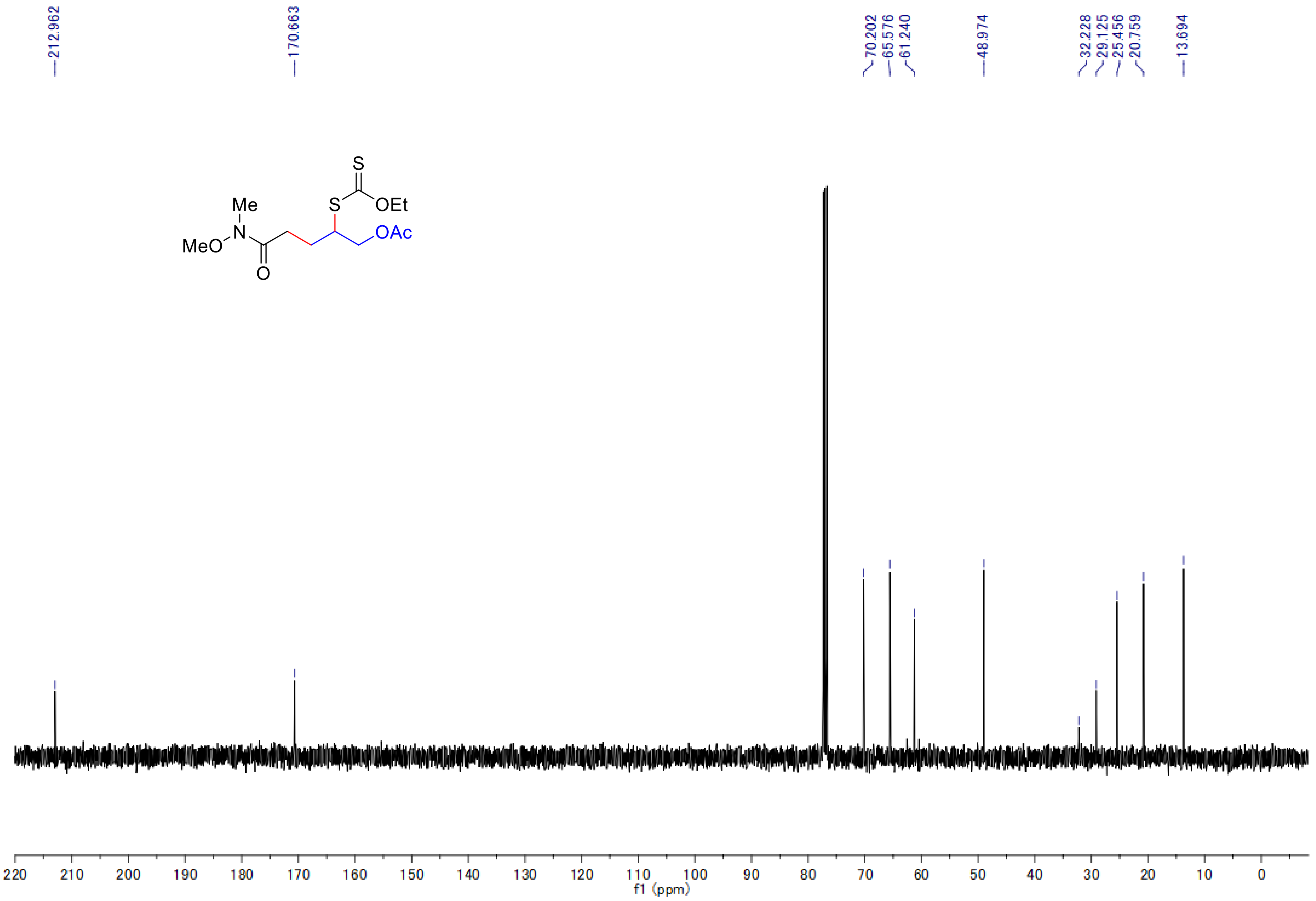

$^{1}$H NMR Spectrum of **3gb** (400 MHz, $CDCl_3$)

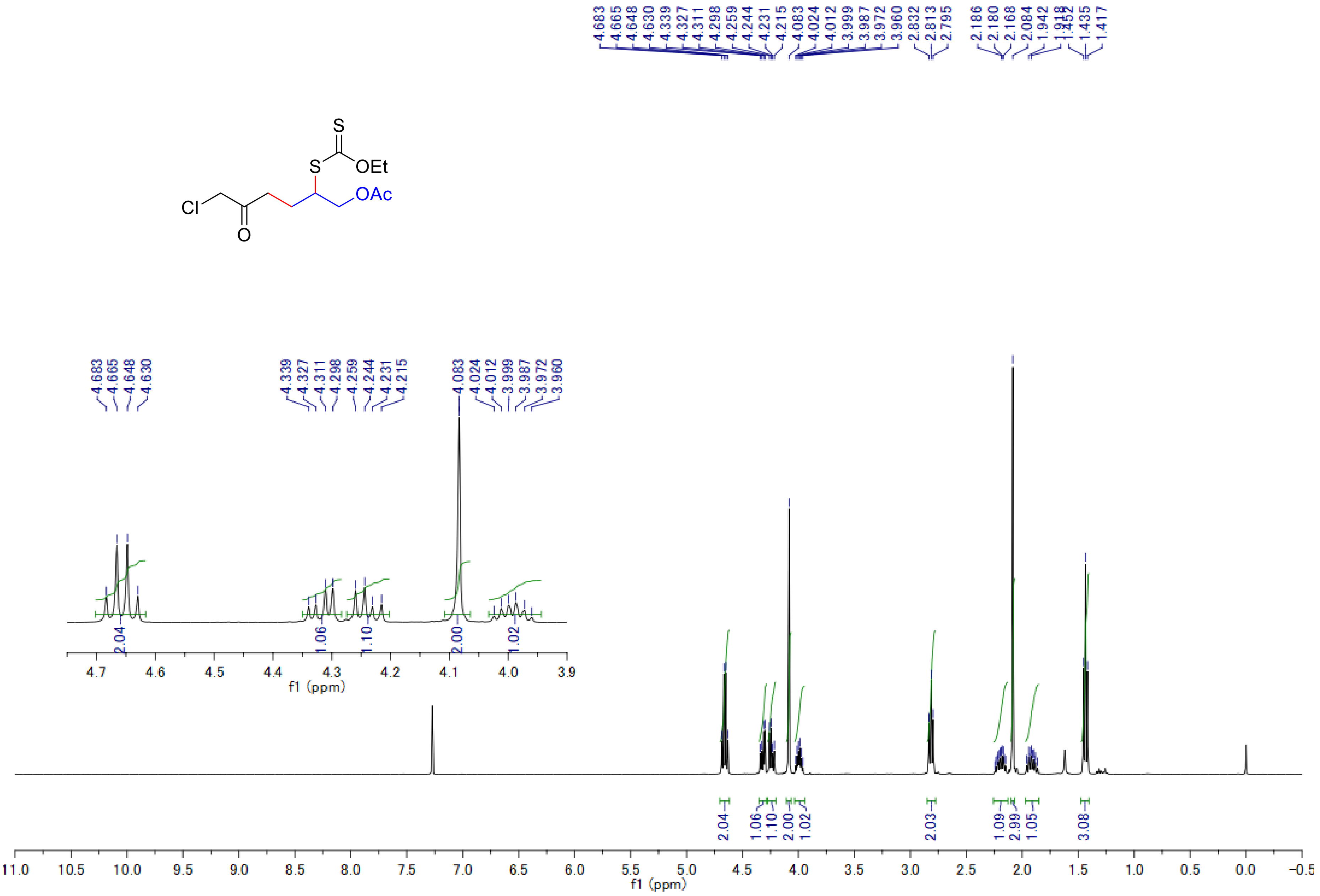

$^{13}$C NMR Spectrum of **3gb** (100 MHz, $CDCl_3$)

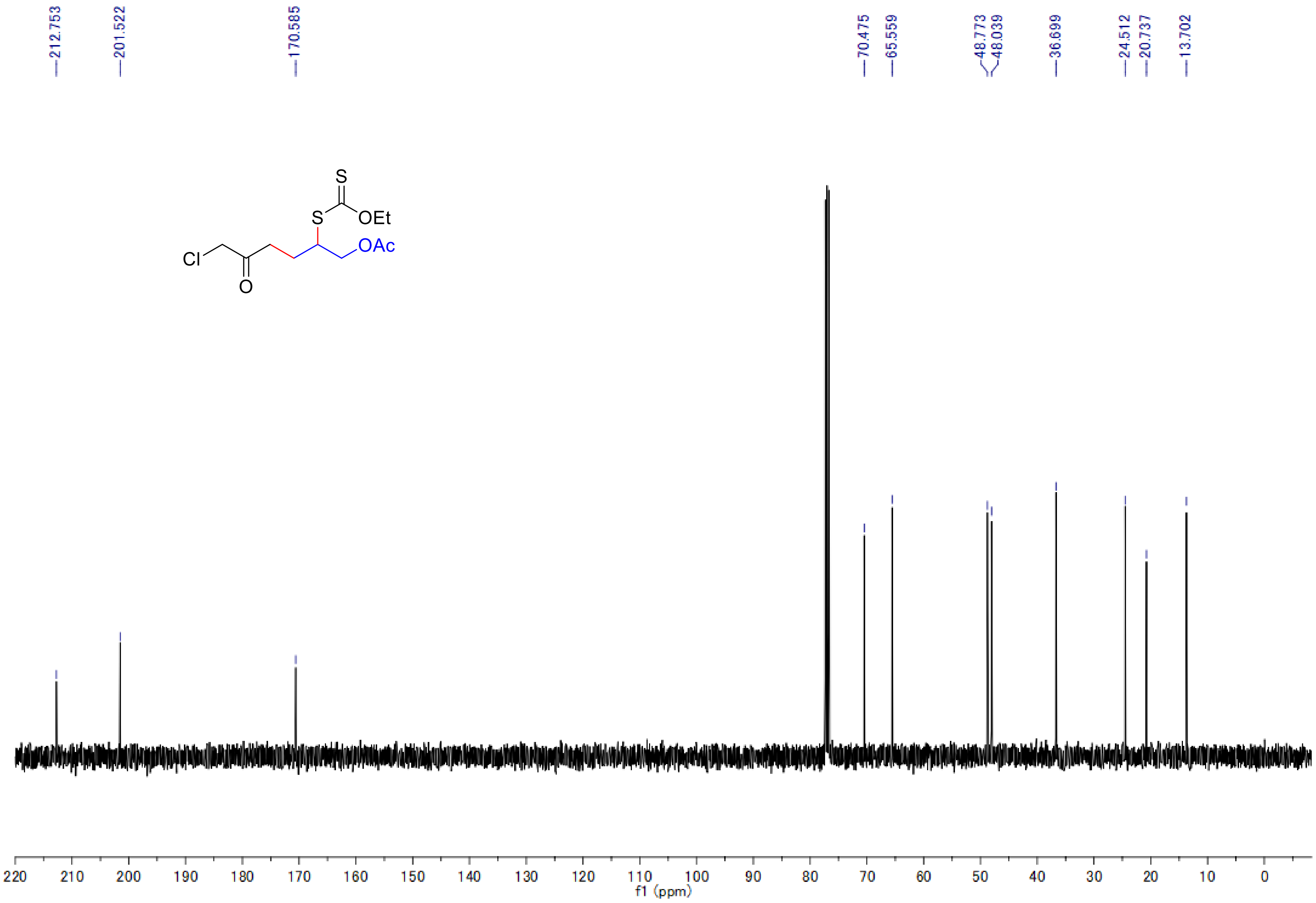

$^{1}$H NMR Spectrum of **3hb** (400 MHz, $CDCl_3$)

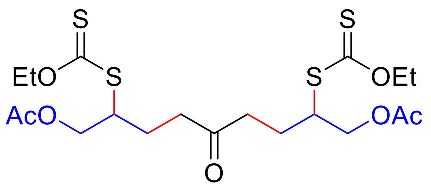


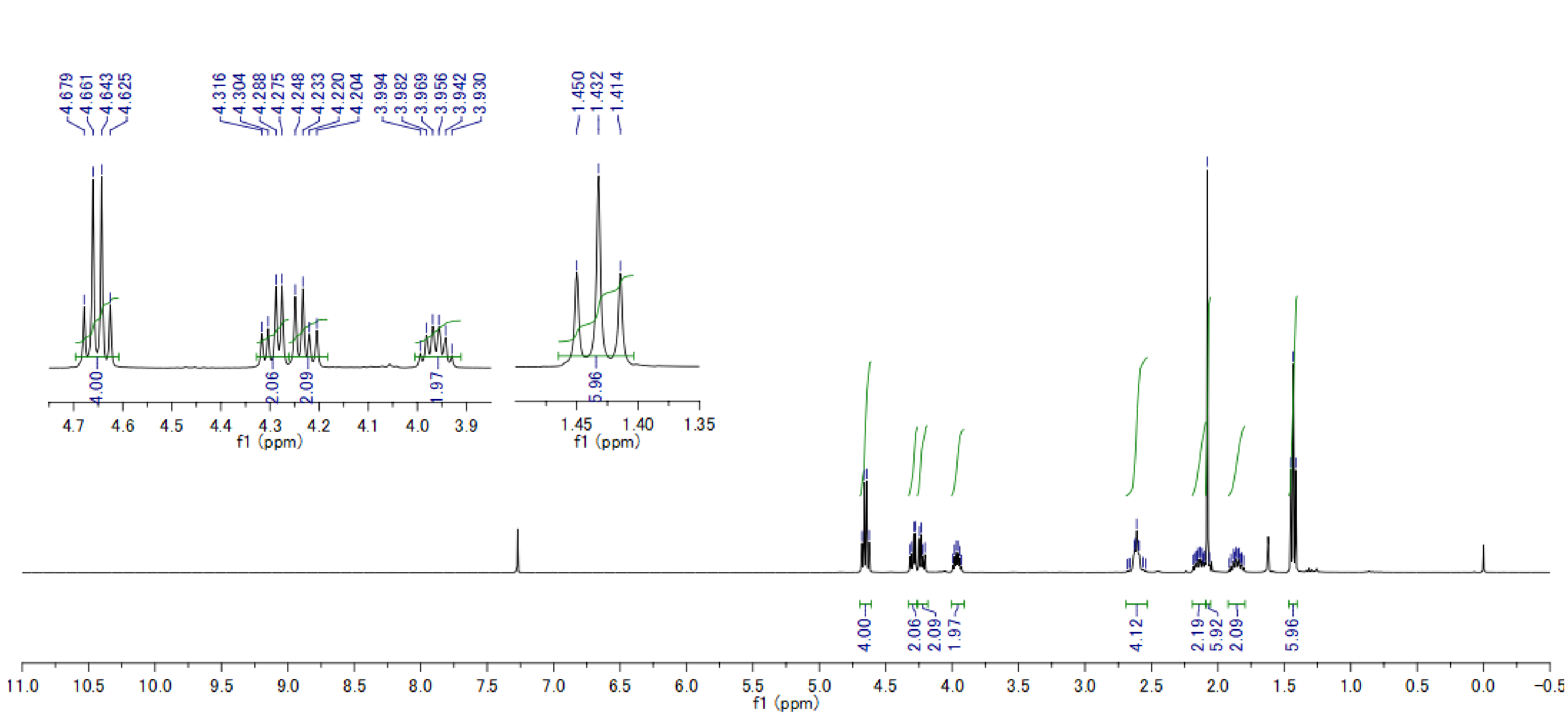

$^{13}C$ NMR Spectrum of **3hb** (100 MHz, $CDCl_3$)

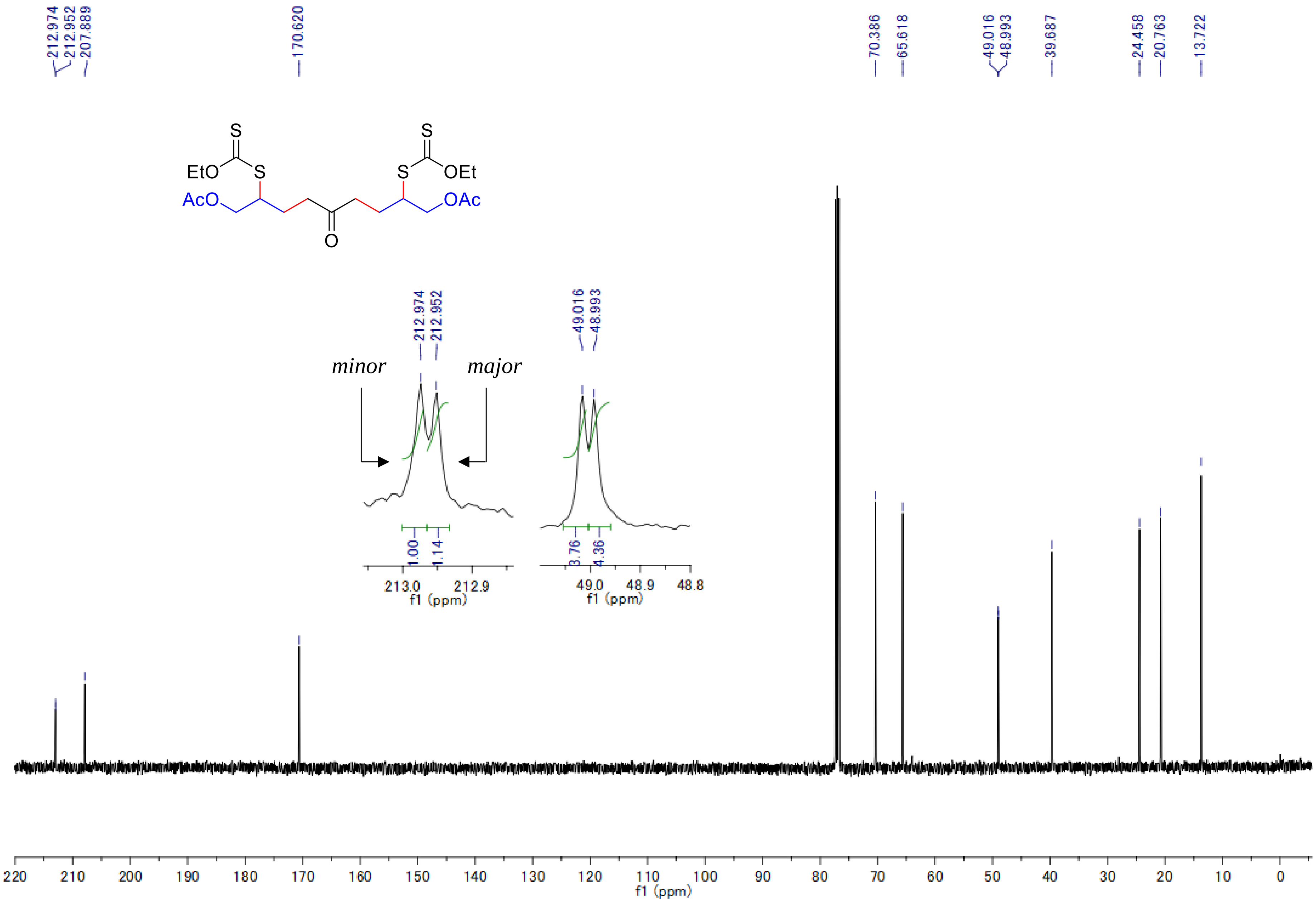

$^{1}$H NMR Spectrum of **3ib** (400 MHz, $CDCl_3$)

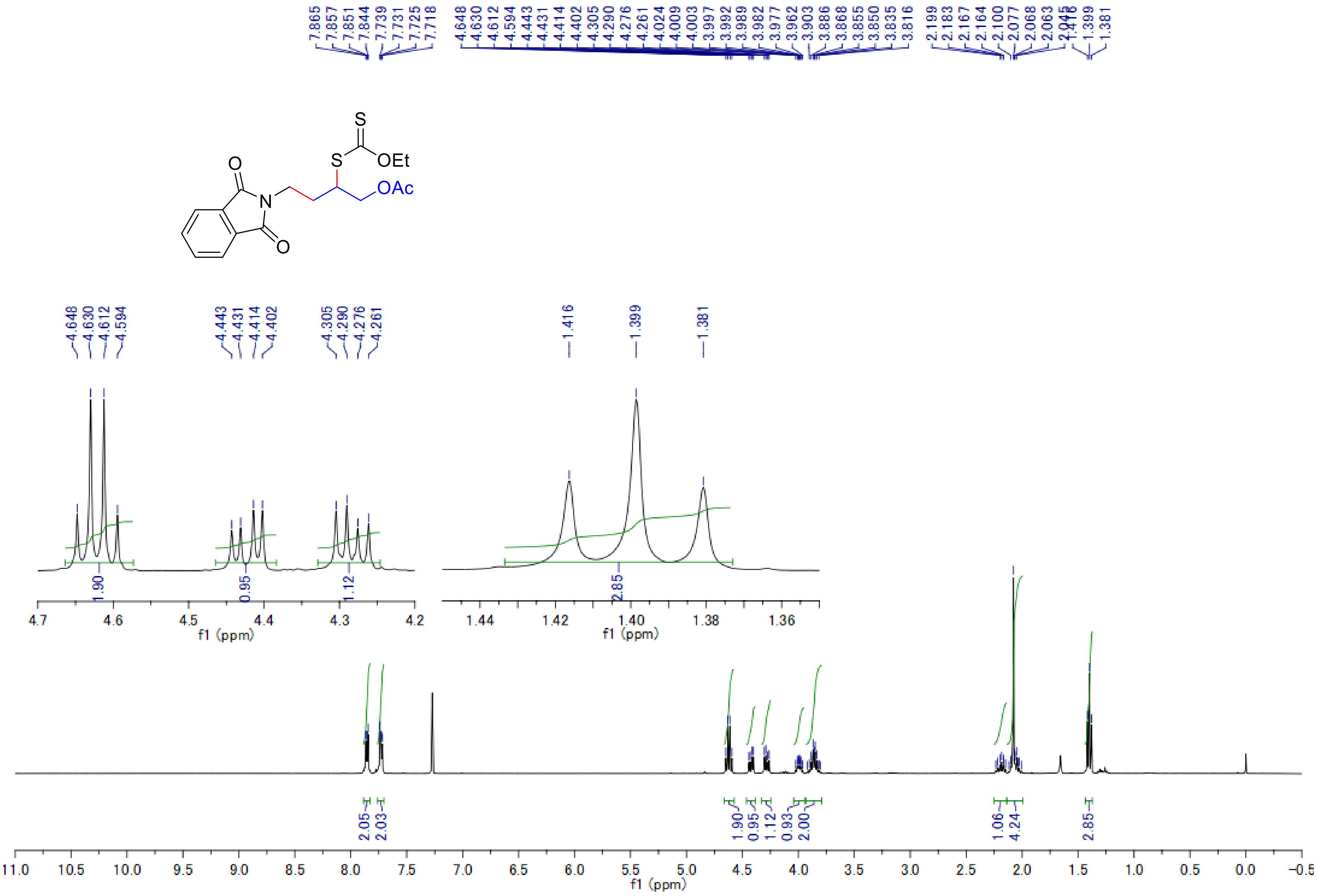

$^{13}$C NMR Spectrum of **3ib** (100 MHz, $CDCl_3$)

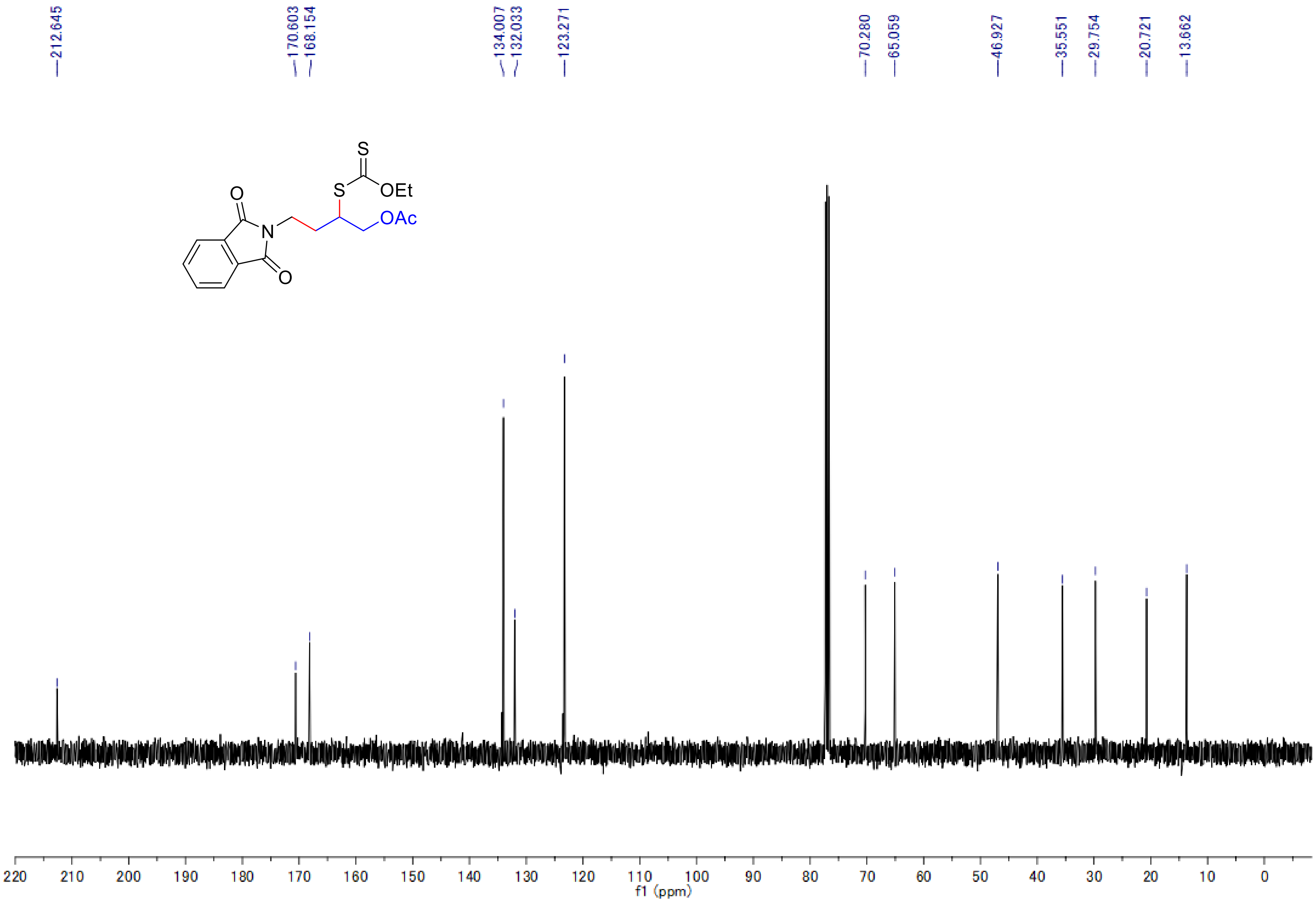

$^{1}$H NMR Spectrum of **3jb** (400 MHz, $CDCl_3$)

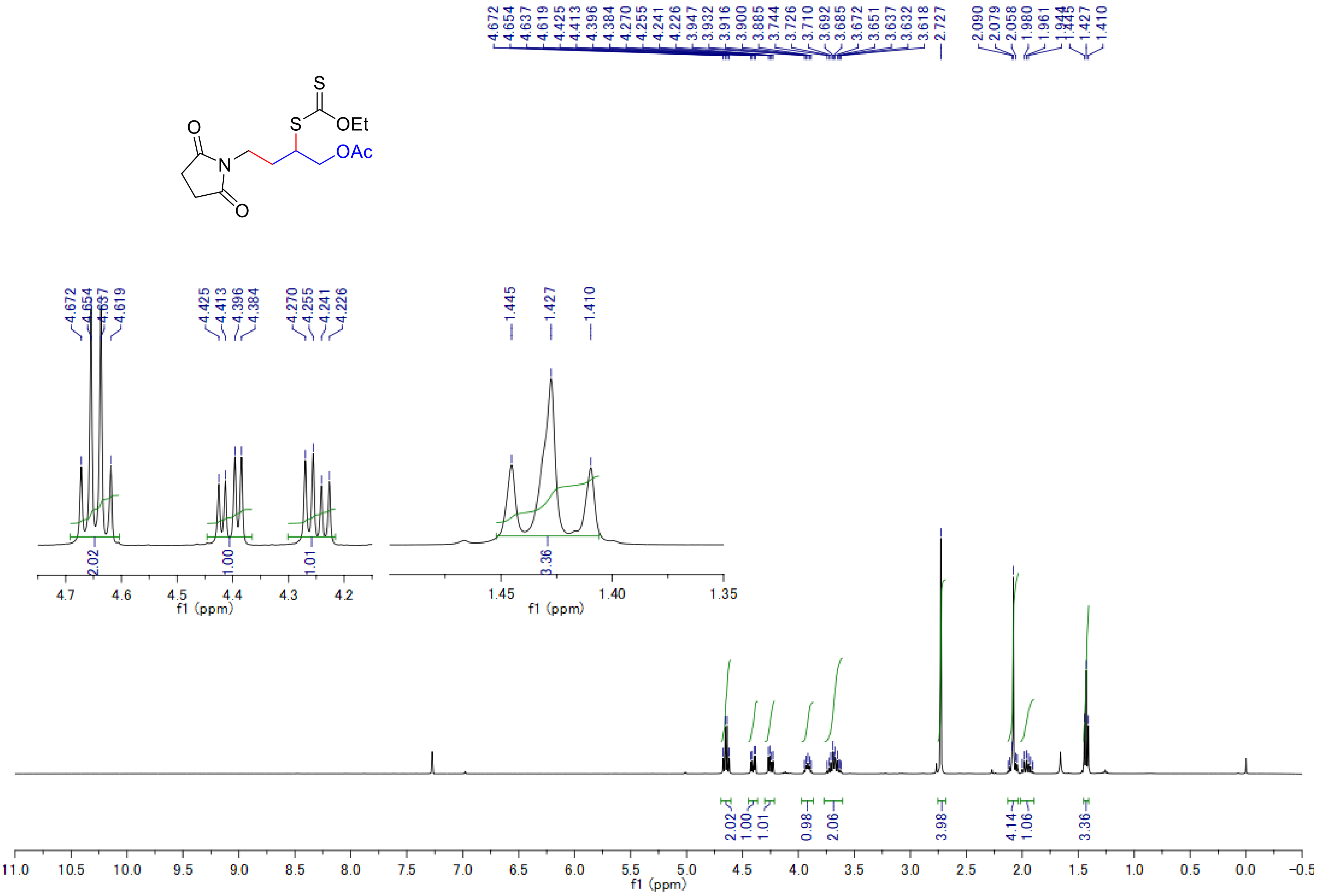

$^{13}$C NMR Spectrum of **3jb** (100 MHz, $CDCl_3$)

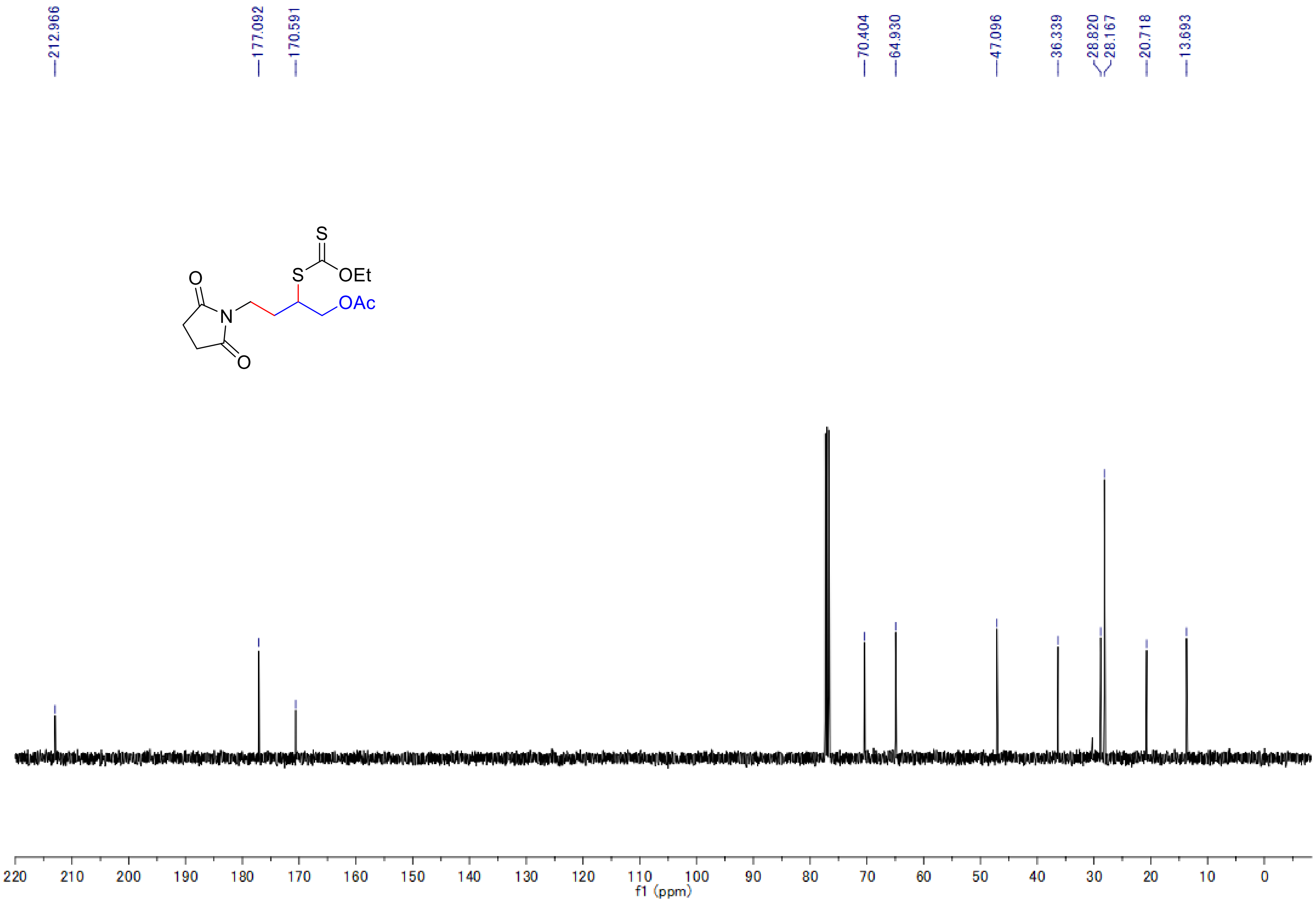

$^{1}H$ NMR Spectrum of **3kb** (400 MHz, $CDCl_3$)

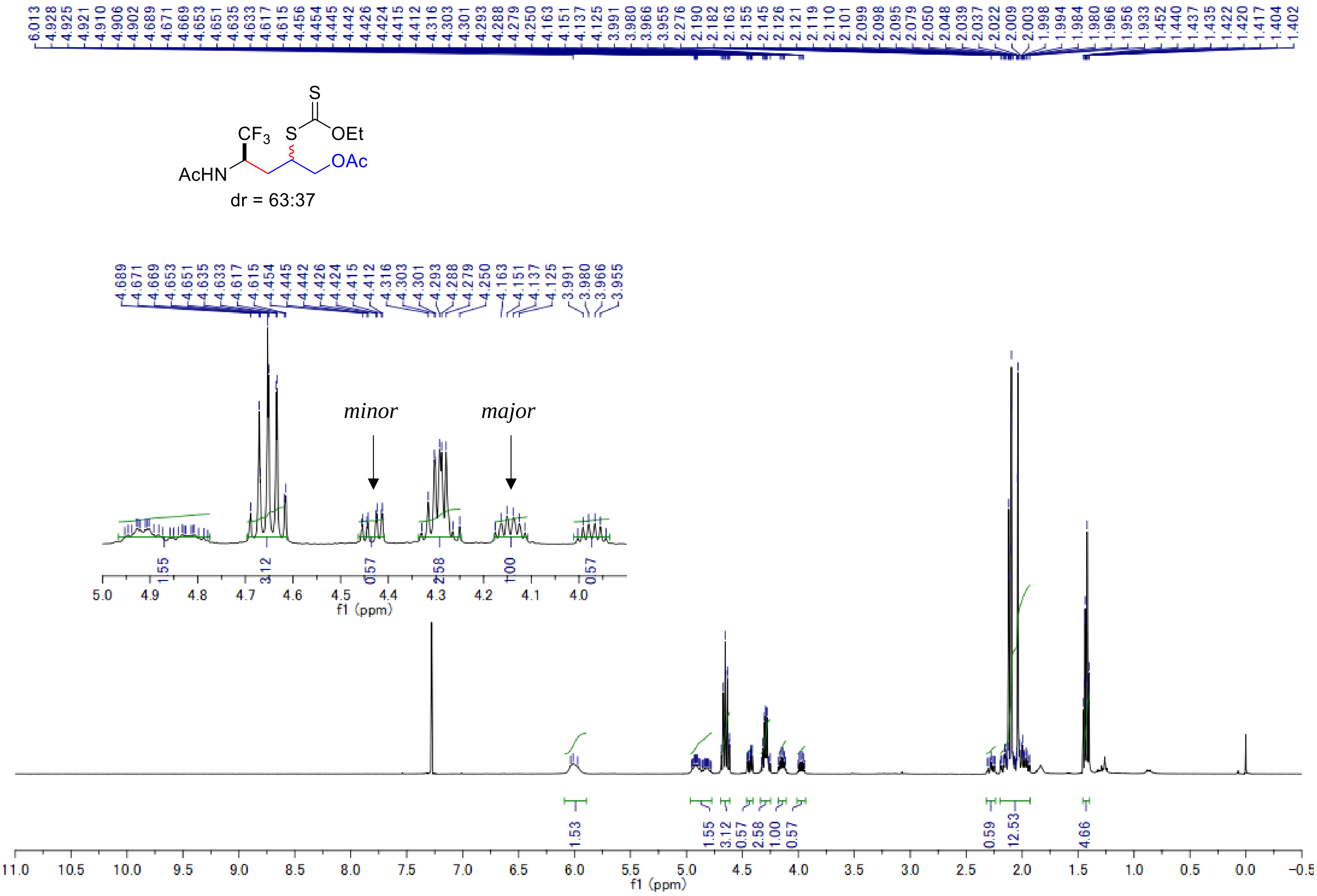

$^{19}$F NMR Spectrum of **3kb** (377 MHz, $CDCl_3$)

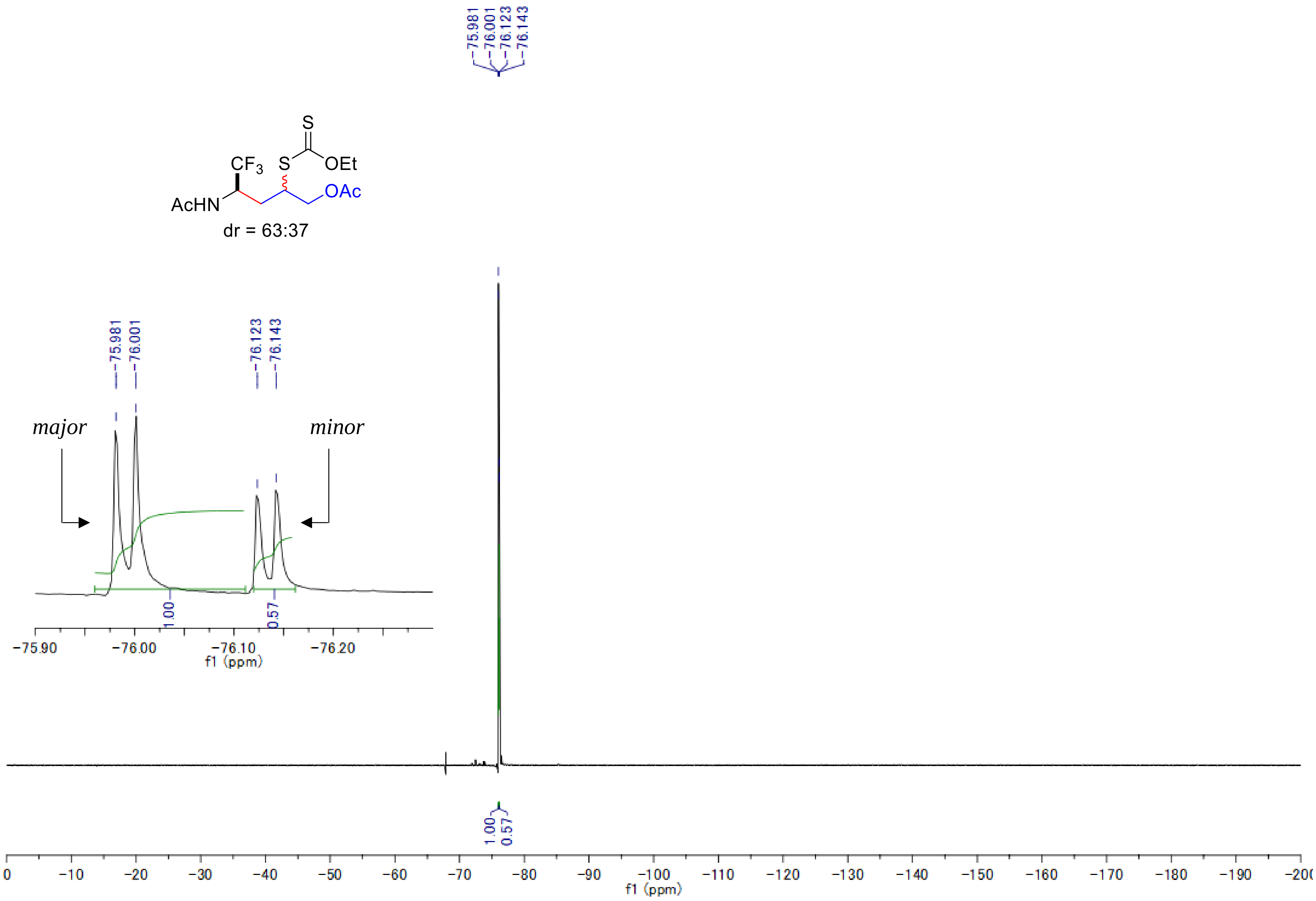

$^{1}$H NMR Spectrum of major-**3kb** (400 MHz, $CDCl_3$)

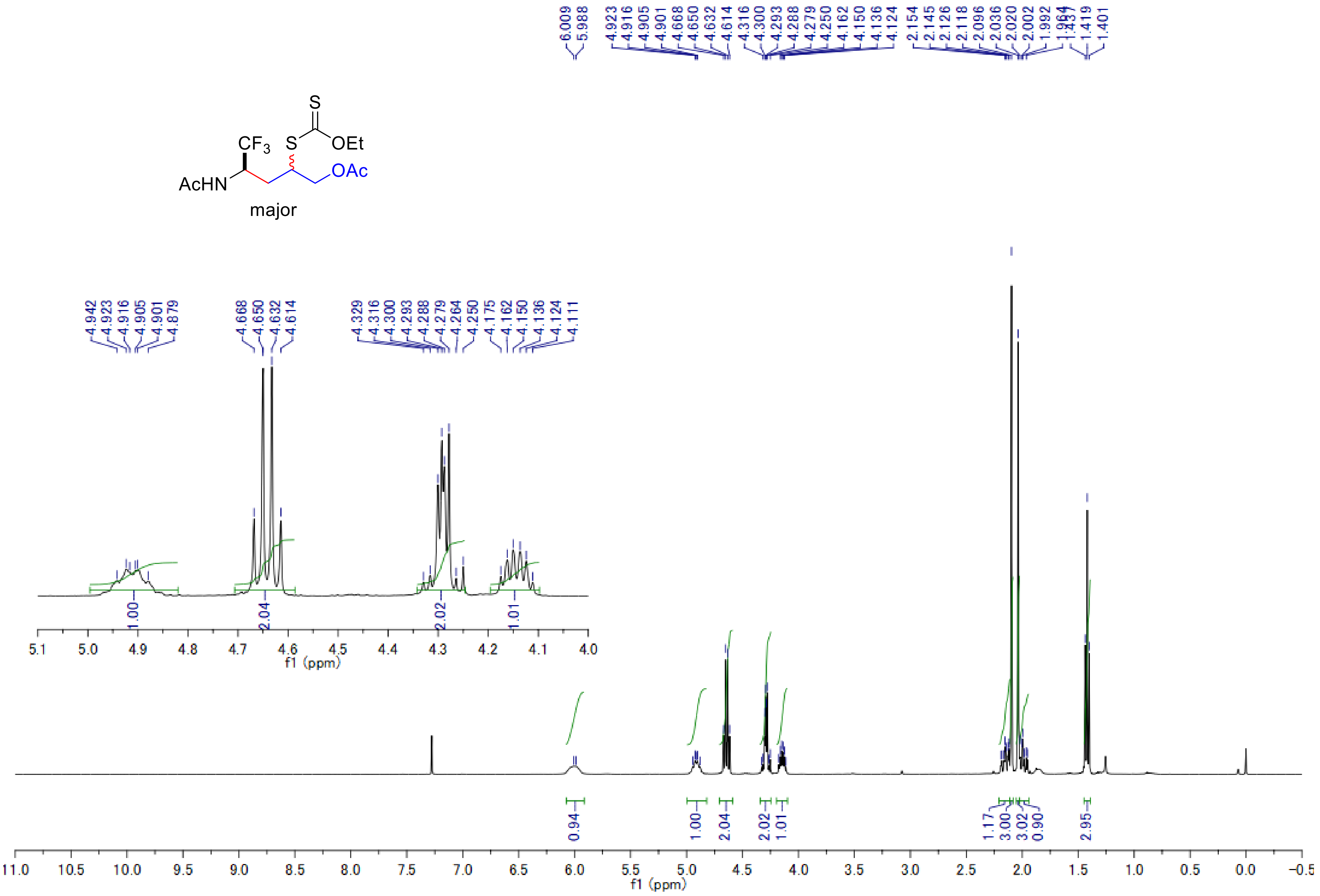

$^{13}$C NMR Spectrum of major-**3kb** (100 MHz, $CDCl_3$)

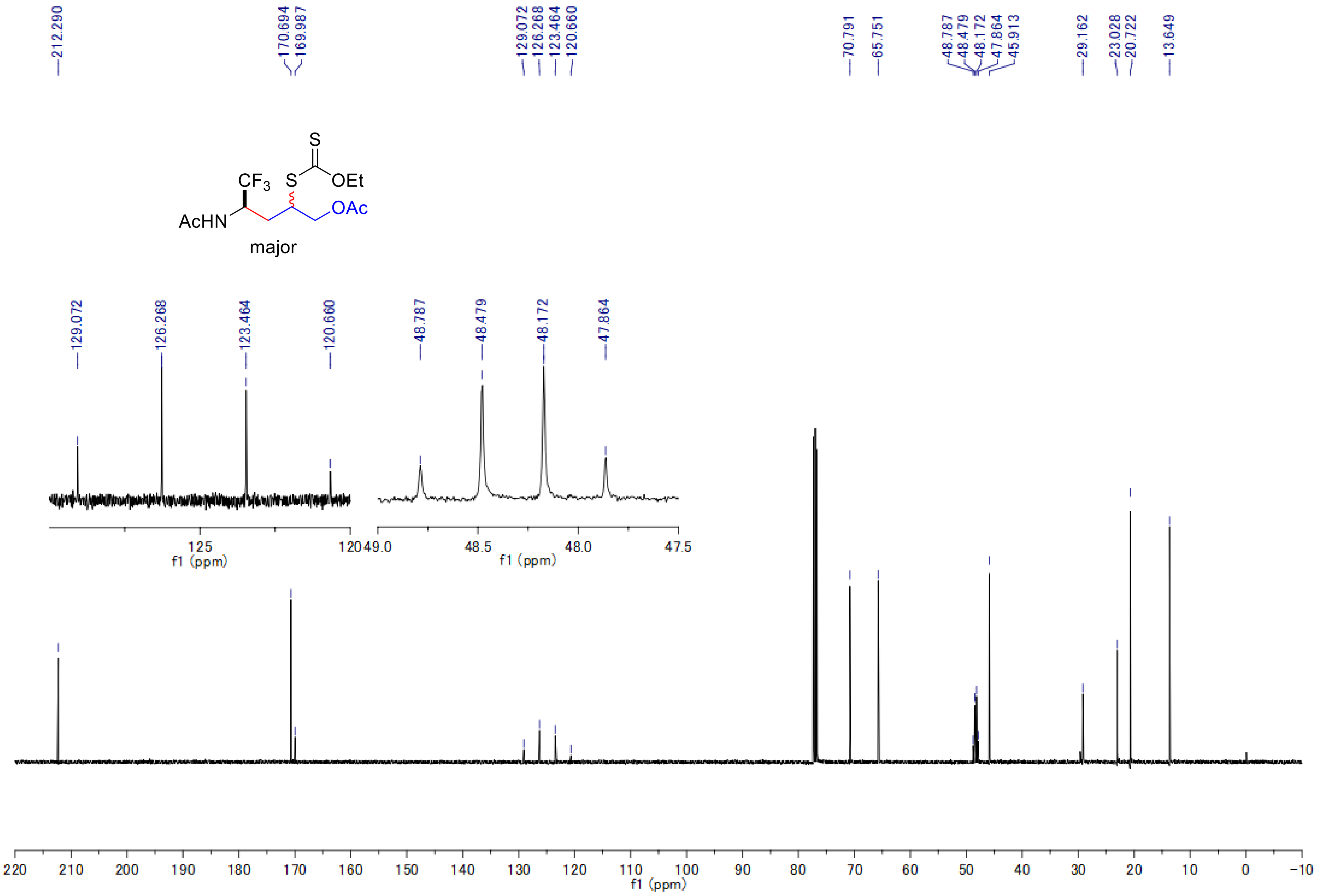

$^{19}$F NMR Spectrum of major-**3kb** (377 MHz, $CDCl_3$)

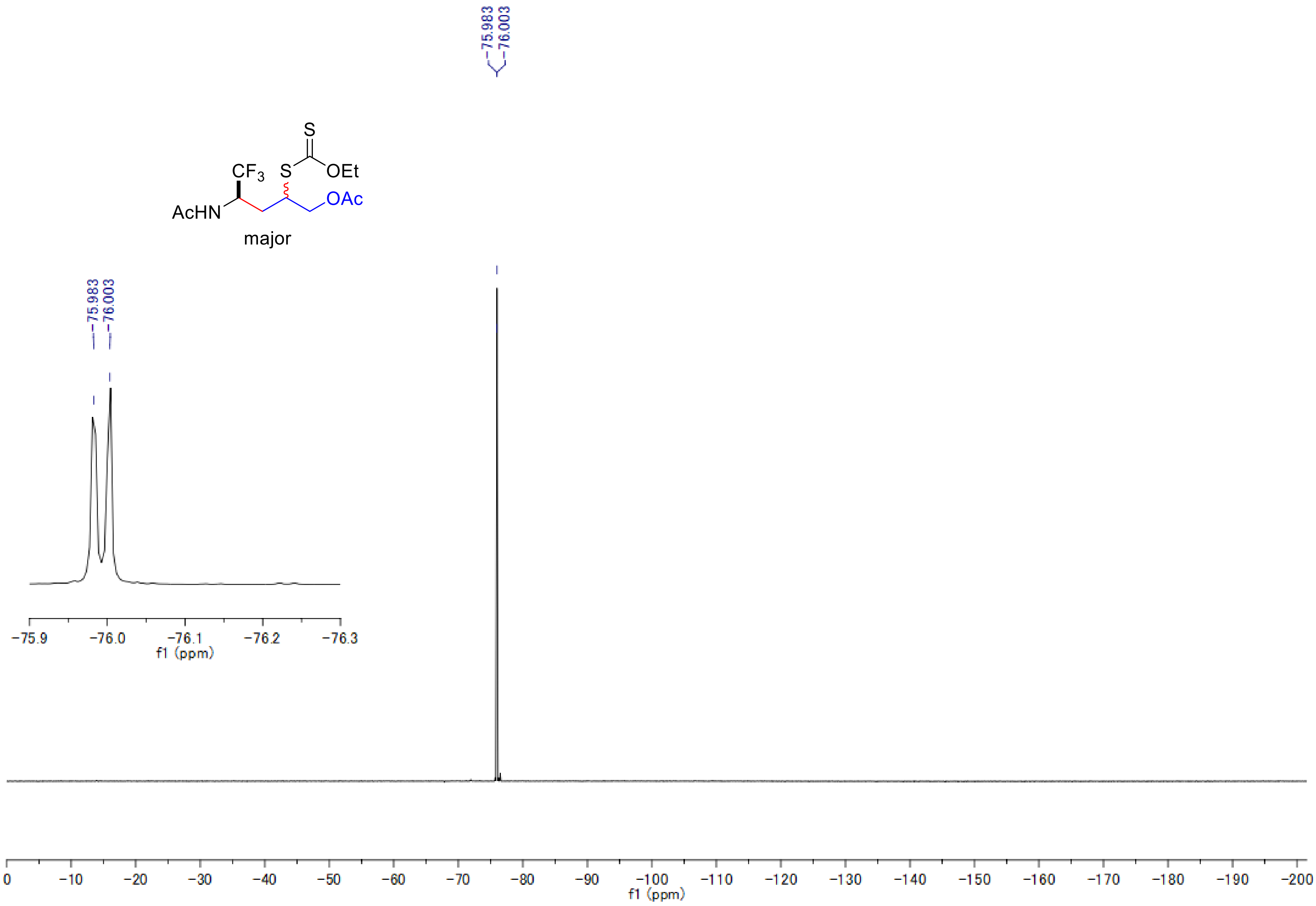

$^{1}$H NMR Spectrum of minor-**3kb** (400 MHz, $CDCl_3$)

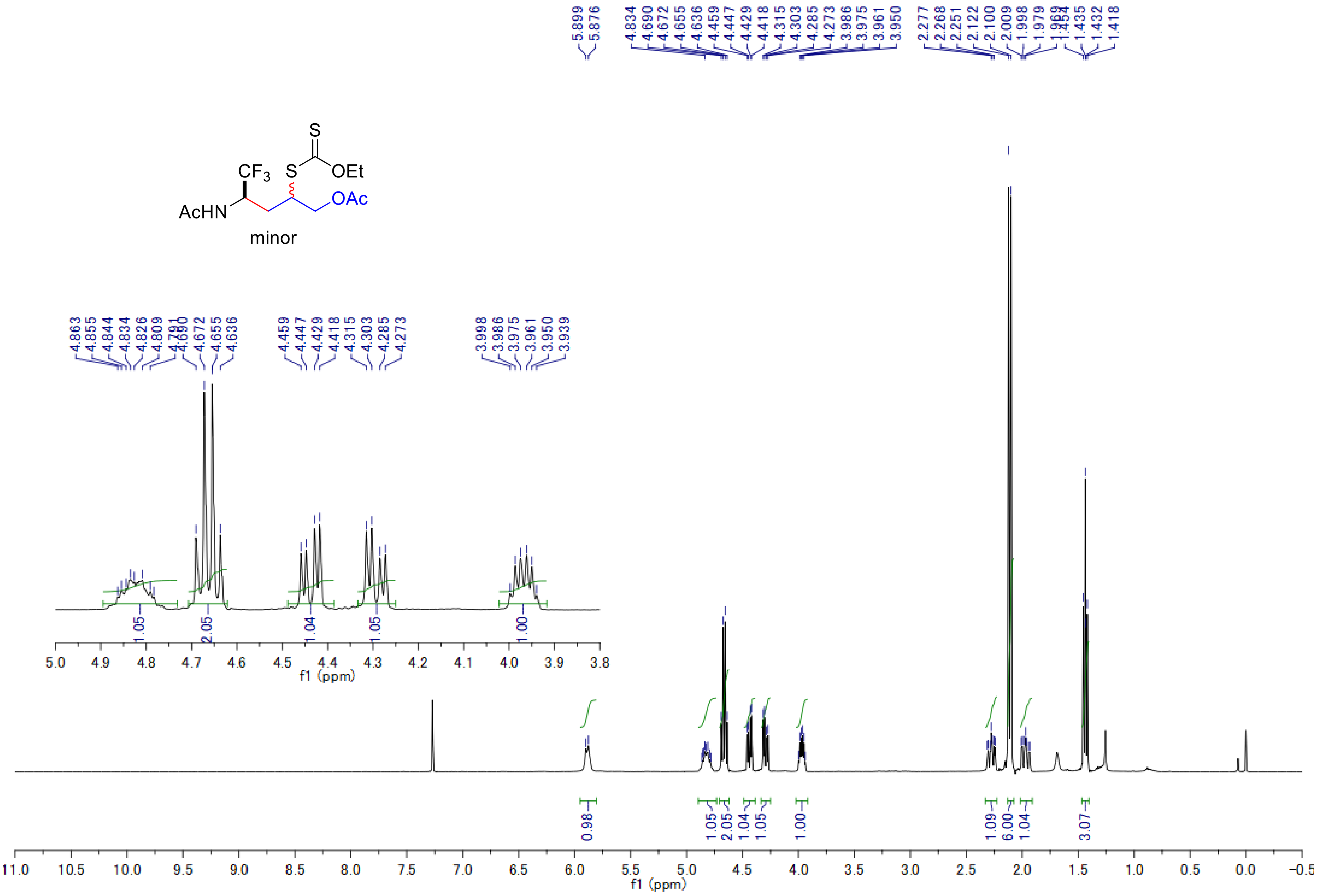

$^{13}$C NMR Spectrum of minor-**3kb** (100 MHz, $CDCl_3$)

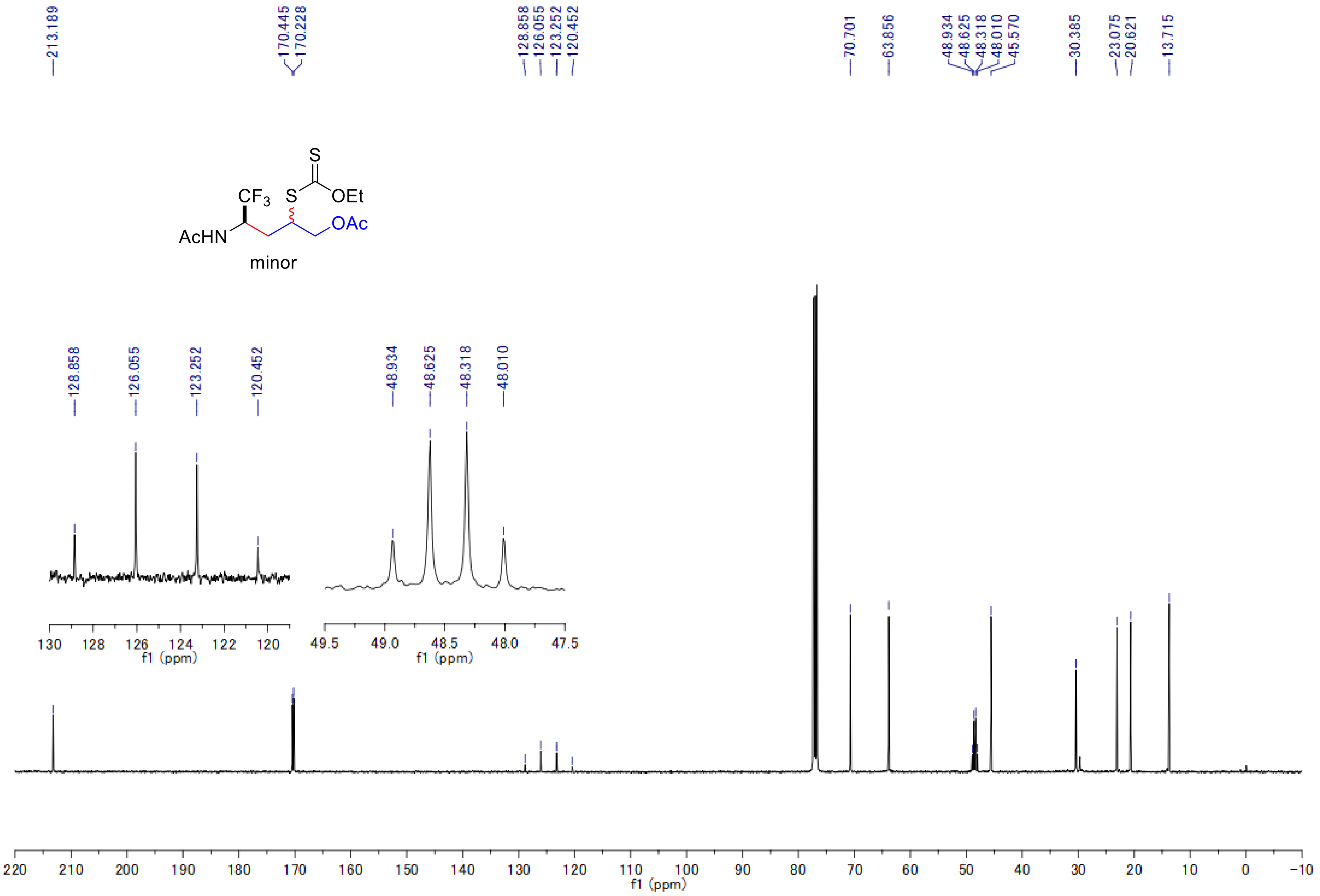

$^{19}F$ NMR Spectrum of minor-**3kb** (377 MHz, $CDCl_3$)

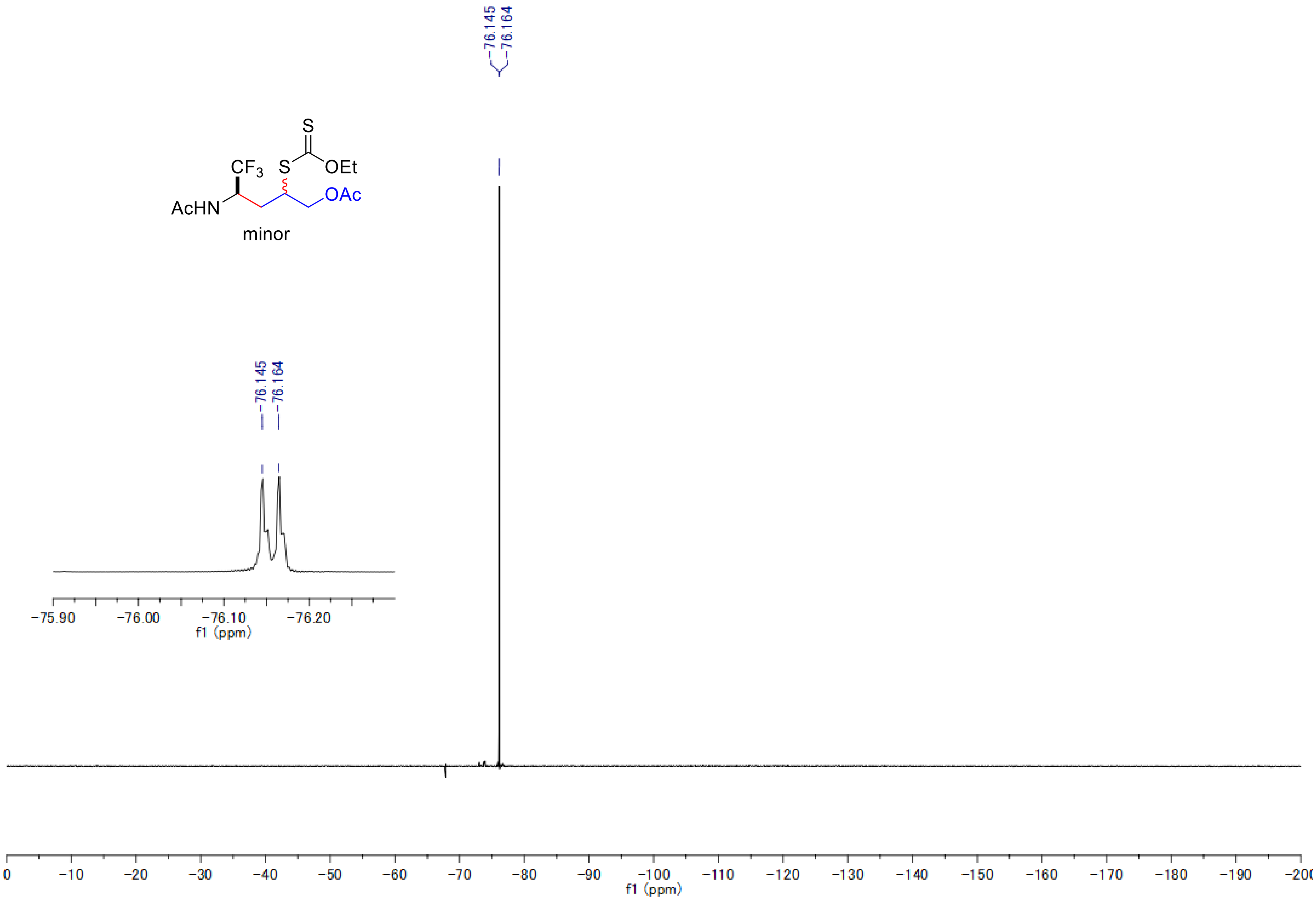

Supplement: File 1 — Full experimental details and analytical data. [file Beilstein_J_Org_Chem-14-3047-s001.pdf]
